# The Interaction between the Tyrosine Kinase Receptor EphA2 and RNF5: Structural Insights from an *In Silico* Approach

**DOI:** 10.34133/csbj.0166

**Published:** 2026-07-17

**Authors:** Marian Vincenzi, Flavia Anna Mercurio, Pasqualina Liana Scognamiglio, Luciano Pirone, Emilia Maria Pedone, Marilisa Leone

**Affiliations:** ^1^ Institute of Biostructures and Bioimaging (CNR), 80131 Naples, Italy.; ^2^ Department of Basic and Applied Sciences, University of Basilicata, 85100 Potenza, Italy.

## Abstract

EphA2 is a tyrosine kinase receptor from the Eph (erythropoietin-producing hepatocellular) family that is involved in several processes associated with regular cellular functions, as well as in different pathological conditions including cancer. EphA2 is up-regulated in many types of tumors, and the process of ligand-triggered receptor endocytosis and the subsequent degradation attract a lot of attention as a means of reducing receptor levels and its pro-oncogenic outcomes. In this context, the intracellular Sam (sterile alpha motif) domain of EphA2 (EphA2-Sam) plays an important role, being the region able to engage protein regulators of receptor endocytosis and stability. Recently, a novel cancer-related interaction involving the E3 ubiquitin ligase RNF5 and EphA2-Sam has been identified. Intriguingly, RNF5 is responsible for lowering receptor stability by targeting it for ubiquitination and consequent degradation. In certain types of breast cancer cells, down-regulation of EphA2 induced by RNF5 overexpression fosters pro-tumorigenic effects while RNF5 silencing and consequent EphA2 amplified regulation induces anticancer outcomes. Immunoprecipitation experiments point out that the membrane-binding domain of RNF5 interacts with EphA2-Sam, but detailed structural information on this protein–protein association is currently missing. To obtain fast insights into structural features governing the molecular recognition between RNF5 and EphA2, we set up an *in silico* approach combining structure predictions by the artificial intelligence tool AlphaFold and docking techniques. Such computational strategy, coupled with preliminary experimental validation with RNF5 peptide fragments, provides pilot information that can be exploited to develop original structure-based drug discovery approaches to identify novel anticancer compounds able to modulate the EphA2/RNF5 axis.

## Highlights

• A computational strategy to obtain structural insights into the poorly known interaction between EphA2 and the RNF5 proteins was set up
• *In silico* studies suggest the RNF5 RING and TM2 domains as the best candidates for the interaction with EphA2-Sam
• Preliminary experimental validation with short synthetic peptides indicates that the RNF5 TM2 N-terminal side provides clear but unspecific weak binding
• RNF5 TM2 but not the TM1 domain assumes a perfect helical transmembrane structure topology according to computational resources
• The EphA2-Sam End Helix region is a “universal” binding site able to recruit multiple partners depending on the specific cancer cell context

## Graphical Abstract

**Figure:**
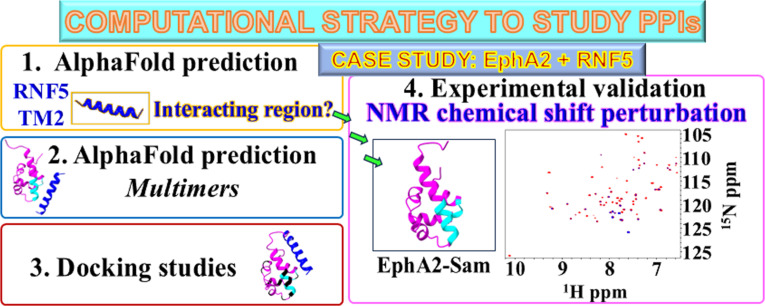

## Introduction

Eph (erythropoietin-producing hepatocellular) receptors [1–11] are the biggest subclass of receptor tyrosine kinases (RTKs).

**Fig. 1.**
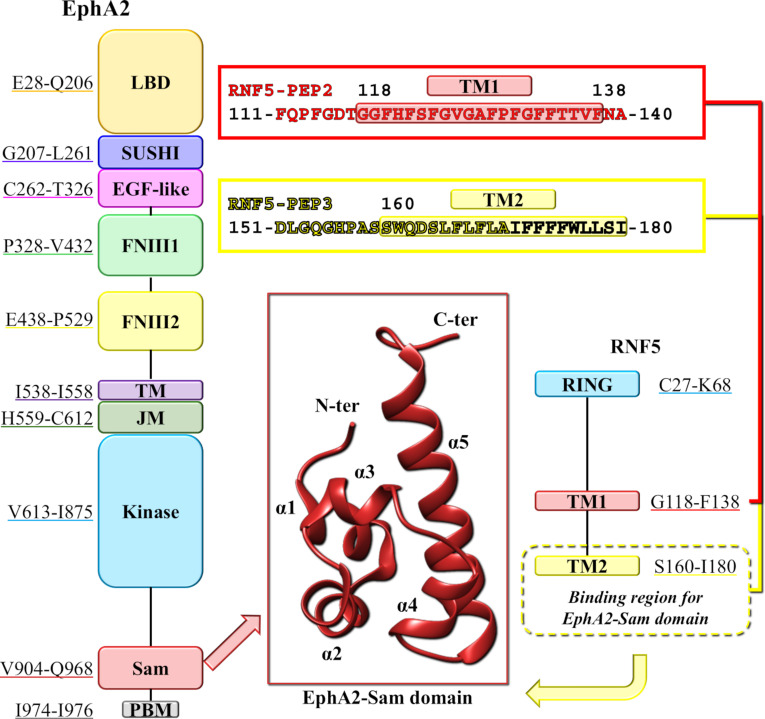
Protein domain organization of EphA2 and RNF5. The EphA2 receptor is composed of a ligand-binding domain (LBD), a SUSHI domain, an epidermal growth factor (EGF)-like domain, 2 fibronectin domains (FNIII1 and FNIII2), a transmembrane region (TM), a juxtamembrane domain (JM), a kinase domain, a sterile alpha motif (Sam) domain, and a C-terminal 3-residue stretch composing the PDZ (PSD-95 [postsynaptic density protein 95], Dlg [Drosophila disc large], and ZO-1 [zonula occludens-1]) binding motif (PBM). The sequence numbers are consistent with those in the UniProt [57] entry P29317 for human EphA2 (see our recent review article [15] for domain ranges). RNF5 includes a zinc-finger RING (really interesting new gene)-type domain followed by 2 transmembrane regions (TM1 and TM2). The sequence numbers and domain limits refer to the UniProt entry Q99942 for human RNF5. The structure of the EphA2-Sam domain (first conformer of the NMR ensemble, PDB ID 2E8N), is shown in the middle. The sequences of the RNF5 C-terminal stretch F111-A140 and D151-I180 are reported, with the portions representing TM1 (G118-F138) and TM2 (S160-I180) regions included in a red and yellow rectangle, respectively, and the sequences of RNF5-PEP2 (residues from F111 to A140) and RNF5-PEP3 (D151-A170) peptides have been underlined.

Among Eph family members, EphA2 represents one of the best characterized receptors due to its multifaceted roles in normal cell physiology and pathological conditions [9,10,12]. In fact, following ligand binding, EphA2 activation generates cytoskeleton remodeling and affects distinct cellular features including growth, differentiation, adhesion, and migration [13]. However, through an articulated signaling network, EphA2 has also been widely associated with a variety of diseases such as cancer, atherosclerosis, viral infections, and cataracts [14–16]. The role of EphA2 in cancer onset and progression is complex and controversial as it can play tumor-promoting or tumor-suppressive functions by fine-tuning a ligand-dependent, principally antioncogenic pathway (“conventional signaling route”) and a ligand-independent and mainly pro-tumorigenic pathway (“unconventional signaling route”) [17]. EphA2 possesses in the extracellular portion (Fig. 1) an ephrin ligand-binding domain [18,19]. Ligand stimulation produces an EphA2 conformational change inducing dimerization [19]. Formation of dimers in turn produces the activation of the kinase domain and consequently cytoplasmic tyrosine residues get phosphorylated and a signaling cascade is initiated [13].

On the other side, the ligand-independent signaling route is associated with phosphorylation of EphA2 receptor by S (serine)/T (threonine) kinases and involves residues positioned in the receptor region T883-E902 that links the kinase and the Sam (sterile alpha motif) domains (Fig. 1). S897 phosphorylation has been well characterized and associated with cancer malignancy promotion.

EphA2 is overexpressed in many tumor types like gastric, esophageal, colorectal, cervical, breast, ovary, prostate, pancreas, neck, renal, lung, bladder cancers, melanoma, and glioblastoma [20–24]. Indeed, the largest EphA2 levels have been faced in tumor cells with the widest invasiveness grade [25]. Up-regulation of EphA2 in cancer is often accompanied by down-regulation of ephrin ligands, thus further supporting the pro-oncogenic nature of the ligand-independent EphA2 signaling pathway [26]. In this context, great attention has been received by the process of ligand-induced receptor endocytosis (i.e., internalization) in cancer cells that is followed by EphA2 degradation [27]. EphA2 endocytosis and subsequent degradation represent a possible route to decrease tumor malignancy by lowering EphA2 up-regulation and its pro-oncogenic effects in cancer cells.

Because of the positive correlation in many cancers between EphA2 silencing and antitumor effects, different molecular tools including specific EphA2 monoclonal antibodies, ephrin-mimetic peptides, or adenovirus-expressing particular ephrin ligands have been generated to foster receptor endocytosis and lower EphA2 levels and its pro-tumor functions [27–33].

Intriguingly, EphA2, like other Ephs, contains a Sam domain at the C-terminus in the intracellular region (Fig. 1). Sam domains are protein interaction modules made up of about 60 to 70 residues with a 5-helix bundle fold (Fig. 1) [12,34]. The Sam domain of EphA2 (EphA2-Sam) is the site where protein regulators of receptor endocytosis and stability are enrolled [27,35].

EphA2-Sam binds the Sam domains of the lipid phosphatase Ship2 (Ship2-Sam) and the first Sam domain of the adaptor protein Odin (Odin-Sam1), which possesses 2 Sam domains in tandem [9,12,36–38]. Sam–Sam homotypic or heterotypic interactions mostly occur through the “Mid Loop (ML)/End Helix (EH)” or “Head to Tail” structure topology [9,12,38,39] in which the middle part of a Sam domain, generally including residues from the α2, α4, and the shorter α3 helices and the related interhelical loop, forms the ML (= “Head”) region, whereas amino acid residues from the C-terminal α5 helix and close loops contribute to the EH (= “Tail”) interaction surface (Fig. 1 and Fig. S1) [9,12,38–40].

The association between EphA2-Sam and Ship2-Sam should correlate mainly with pro-oncogenic effects in cancer cells due to Ship2 capacity to decrease the tumor suppressor EphA2 functions, which rely on the ephrin ligands, whereas it favors a ligand-independent pro-migratory action of EphA2 [41]. Regarding the interaction between Odin and EphA2, a clear connection to cancer has not been established, but it is known that Odin through the heterotypic Sam–Sam interaction enhances receptor stability by regulating somehow the receptor ubiquitination process [35].

Another interactor of EphA2-Sam is the tumor suppressor protein SASH1 (Sam and SH3 domain containing 1) [42]. SASH1 has 2 Sam domains, and the first N-terminal Sam domain is mainly responsible for the interaction with Eph receptors [42]. The interaction between EphA2-Sam and SASH1 has not been well experimentally characterized from the structural and functional point of view.

Another interaction that was recently discovered and that regards modulation of receptor stability/degradation involves the EphA2 receptor and the E3 ubiquitin ligase RNF5 (RING finger protein 5) [43].

RNF5 is anchored to the Endoplasmic Reticulum (ER) and plays several functions by favoring ubiquitination of diverse substrates; it is first of all involved in the clearance of not properly folded proteins by ERAD (endoplasmic reticulum-associated degradation) [44], and it negatively regulates ER stress and autophagy [45].

It has been demonstrated that RNF5 is up-regulated in breast cancer specimens where it contributes to poor survival, but it also seems that increased RNF5 levels are a more general hallmark of tumor progression being for instance also encountered in ovarian and renal cancer-derived cell lines as well as melanoma, acute myeloid leukemia, and hepatocellular carcinoma [43,46,47]. However, a controversial function in cancer can be associated with RNF5 as both cancer-promoting and -inhibitory roles can be attributed to the protein in different tumors and based on the cellular context or the substrate that is ubiquitinated [43,47]. Binding between RNF5 and EphA2 further validated RNF5 relevance in cancer. RNF5 induces EphA2 ubiquitination and consequent degradation, thus lowering receptor stability and its levels at cell surface. In HER2 (human epidermal growth factor receptor 2)-negative breast cancer cells, larger amounts of EphA2 induced by RNF5 inhibition correlate with lower ERK (extracellular signal-regulated kinase) phosphorylation and larger p53 expression. Accordingly, RNF5 negative regulation enhances the adhesion and lowers the migration of HER2-negative breast cancer cells, and after RNF5 silencing, a decrease in the growth of xenograft tumors resulting from EsRe (estrogen receptor)-positive, HER2-negative breast cancer cells characterized by higher EphA2 levels is observed. Thus, in breast cancers, coexistence of EphA2 up-regulation and RNF5 down-regulation correlates with improved survival of patients affected by EsRe -positive, HER2-negative breast cancer. This work highlighted that in HER2-negative breast cancers RNF5 modulates in a negative manner EphA2 functions and inhibits its tumor-suppressive role [43]. RNF5 contains within its protein domain arrangement a C-terminal transmembrane region that serves as anchorage to the ER apart from binding with several substrates (TM2 in Fig. 1) [44,48].

Binding of RNF5 to EphA2 was first highlighted through affinity purification and mass spectrometry investigation of RNF5 protein interactors [43]. Co-immunoprecipitation studies confirmed the protein/protein interaction in 293T cells overexpressing EphA2 following overexpression of RNF5 and was further observed in MCF-7 cells. Studies with truncated protein forms revealed that the Sam domain of EphA2 was responsible for binding to the C-terminal transmembrane region of RNF5 (i.e., residues 165 to 180). Thus, it seems that the interaction between EphA2 and RNF5 necessitates the membrane anchoring ability of RNF5 but not the E3 ubiquitin ligase function.

As binding of RNF5 to EphA2 is essential to modulate receptor stability and its cancer-related pathways by influencing receptor serine and tyrosine phosphorylation status, molecules able to modulate the formation of the RNF5/EphA2 complex might work as anticancer therapeutics. The design of such compounds requires detailed knowledge of the topology of the interaction between EphA2 and RNF5 and the related structural features that are necessary to set up ad hoc structure-based drug discovery strategies. To date, experimental high-resolution 3-dimensional (3D) structures of the EphA2/RNF5 complex are missing; thus, to get structural insights into this cancer-related novel protein–protein interaction (PPI) involving EphA2 receptor, herein we establish an *in silico*-based approach that employs the artificial intelligence structure prediction tools AlphaFold2 (AF2) [49,50], and its more powerful recent version AlphaFold3 (AF3) [51,52], along with canonical docking techniques with the Haddock webserver [53,54] to analyze, from the structure point of view, diverse RNF5 regions in the apo form and bound to EphA2-Sam. The *in silico* generated data were preliminary validated by experimental conformational and interaction studies with a peptide fragment from the TM2 region revealing that the very C-terminal part of TM2 encompassing residues from 171 to 180 (Fig. 1) might be important for high affinity and specific interactions between the transmembrane region and EphA2-Sam. Nevertheless, the *in silico* studies also pointed out that the EH surface of EphA2-Sam that is involved into interactions with other Sam domains and/or close adjacent receptor regions might be involved also into binding to RNF5. Finally, our work indicates that the interaction between the RNF5 TM2 and EphA2-Sam might require a relevant RNF5 structure rearrangement that, by changing the orientation of the diverse RNF5 protein regions, makes engagement of TM2 by EphA2-Sam more favored with respect to other protein sites while keeping the RNF5 RING domain exposed and able to be engaged in its ubiquitin transfer activities. On the other side, based on the poor amount of experimental data on the interaction in between EphA2-Sam and other RNF5 domains that have been collected thus far, a direct interaction between the RNF5 RING domain and EphA2-Sam cannot be completely excluded. Such interaction is supported by our computational results and might occur in the presence or absence of a simultaneous TM2 binding, but of course, this scenario requires proper experimental validation.

## Materials and Methods

### Generation of AF2 models

AF2 [49,55] was first run through the ColabFold [56] server (https://colab.research.google.com/github/sokrypton/ColabFold/blob/main/AlphaFold2.ipynb#scrollTo=kOblAo-xetgx, accessed 2025 July 8) to predict models of the entire RNF5 protein (residues M1-I180 from UniProt [57] entry Q99942 for human RNF5) and its isolated fragments including the RING domain (residues C27-K68), transmembrane region 1 (TM1) (residues G118-F138), transmembrane region 2 (TM2) (residues S160-I180), and the “TM1+TM2” region (residues G118-I180). AF2 [49,55] predictions were achieved by employing homologous structure templates (i.e., the “PDB100” option was selected for “template_mode” value) and by setting “alphafold2_ptm”, 48, 0.0, 0 (i.e., unlimited), “512:1024” and “1” values for “model_type”, “num_recycles”, “recycle_early_stop_tolerance”, “relax_max_iterations”, “max_msa”, and “num_seeds” parameters, respectively [50,56]. Default values were used for the remaining settings. For each protein region, 5 structures were predicted, and all of them were exposed to post-prediction relaxation by means of gradient descent in the Amber force field [56,58]. The resultant 5 monomeric models obtained for the diverse protein regions were ranked according to the pLDDT (predicted local distance difference test) score [59].

Then, to predict the model of EphA2-Sam (residues V904-I976 from UniProt [57] entry P29317 for human EphA2) in complex with the different RNF5 regions, i.e., the RING domain (residues C27-K68 from human RNF5), the isolated TM1 and TM2 segments (residues G118-F138 and S160-I180, respectively), the combined “TM1+TM2” segment (residues from G118 to I180), and the entire protein (residues M1-I180), AF2 [49,55] was again run through the ColabFold (v1.5.5) server [56] (accessed 2025 June 10), by employing identical parameters to those used for single domain predictions but setting “alphafold2_multimer_v3” as “model_type” [59].

To analyze aggregation phenomena and predict the homodimeric models of the entire RNF5 protein (residues M1-I180 from human RNF5) as well as the RNF5-PEP3 peptide (residues D151-A170), AF2 [49,55] was again run through the ColabFold [56] server (accessed 2026 January 21). AF2 [49,55] predictions were achieved by employing the same parameters implemented to get models of the EphA2-Sam in complex with diverse RNF5 domains [50,56].

The models of each complex were ranked based on the predicted template modeling (pTM) and interface predicted template modeling (ipTM) values following the formula 0.2 × pTM + 0.8 × ipTM [59].

### Generation of AF3 models

The AlphaFold3 server [51,52] (https://alphafoldserver.com/, accessed 2025 June 10) was also employed to predict models of the entire RNF5 protein, its RING domain, and the TM1, TM2, and TM1+TM2 regions (residues M1-I180, C27-K68, G118-F138, S160-I180, and G118-I180, respectively, from UniProt [57] entry Q99942 for human RNF5). The entire RNF5 protein and its RING domain were predicted in the presence of 2 Zn^2+^ ions. Concerning the parameters established for structure predictions in the presence of Zn^2+^ ions, the “Protein” and “1” values were chosen for the “Entity type” and “Copies” options in the entity type 1 section, and “Ion” and “2” values for the “Entity type” and “Copies” options in the entity type 2 section. Moreover, in all AF3 runs, the “Seed” parameter was set to the default value.

AF3 with default settings was also employed to predict models of EphA2-Sam (residues V904-I976 from UniProt [57] entry P29317 for human EphA2) in complex with the different RNF5 regions. The models are ranked starting from their pTM, ipTM, disorder (i.e., scalar value in the range 0 to 1 quantifying the disorder grade of the predicted structure), and has_clash (i.e., Boolean value pointing out if a relevant number of clashing atoms occur in the predicted structure) values and according to the formula 0.8 × ipTM + 0.2 × pTM + 0.5 × disorder − 100 × has_clash [51,52].

### Docking studies: The TM2/EphA2-Sam SHIFTENTER complex

#### EphA2-Sam structure editing

3D coordinates of the Sam domain of human EphA2 receptor, which were employed for docking studies to predict protein–protein/protein–peptide interactions, derived from those of the first conformer of the NMR (nuclear magnetic resonance spectroscopy) ensemble of structures with Protein Data Bank (PDB) entry code 2E8N, after the removal of the flexible N- and C-terminal tails (i.e., residues 1 to 9 and 83 to 88, respectively, following the 2E8N entry sequence numbering). Consequently, the final EphA2 amino acid sequence included in our studies consisted of residues from V904 to I976 of human EphA2 (UniProt entry code P29317). Flexible tails removal was achieved with the “structure editing” tool of UCSF ChimeraX [60] (version 1.5). The NMR structure corresponding to the 2E8N PDB entry includes within the EphA2-Sam amino acid sequence the non-natural mutation V944I; consequently, residue 944 was changed to the wild-type residue I944 by editing the PDB file where the 3-letter amino acid code referring to the specified amino acid was moved -to ILE prior to submitting the file to Haddock [61].

#### Docking protocol

Docking studies to predict binding poses for EphA2-Sam in complex with the RNF5 TM2 region were performed with the Haddock 2.4 webserver (https://rascar.science.uu.nl/haddock2.4/, accessed 2025 July 26) [53,54], starting from the NMR structure of EphA2-Sam (first conformer of the NMR ensemble, PDB code 2E8N, edited as specified in the “EphA2-Sam structure editing” section) and that of the RNF5 TM2 peptide (best AF2 model). The protonation states of the 2 histidine residues H924 and H954 from the input EphA2-Sam structure were retained by selecting “HISD” in the “Input parameters” section of Haddock 2.4 webserver [54]. Active residues for EphA2-Sam corresponded to those having a solvent exposure larger than 40%, as evaluated with the software MolMol 2K.2 [62]: R907, K917, E923, A927, E934, Q938, R946, R950, P952, K956, R957, Y960, N970, V972, I974, P975, and I976, whereas for the short TM2 peptide region, all residues were considered to be active.

Given the presence of several exposed C-terminal and PBM residues (i.e., N970, V972, I974, P975, and I976) among the active ones, noticing that many docking solutions were biased toward those residues, a second docking run was conducted using an identical protocol but by excluding them from the active residue list.

The docking protocol included a rigid-body energy minimization during which 1,000 structures were calculated, followed by semiflexible simulated annealing of the best 200 solutions, and by a final refinement in water. The 10 best solutions in terms of Haddock scores were visually inspected, and EphA2-Sam/peptide poses representative of diverse binding modes were more deeply analyzed after collecting them in groups of similar solutions (see Results for additional information).

### LigPlot+ analyses

Two-dimensional diagrams of intermolecular interactions in representative Haddock models and in the 5 AF2 predicted structures of the EphA2-Sam/RNF5 TM2 complex were generated with LigPlot+ (version 2.2.8) [63]. H-bonds were identified using the following thresholds: 2.5 Å (H–acceptor distance) and 3.35 Å (donor–acceptor distance). Regarding non-bonded protein–peptide interactions, these were selected by choosing cutoff values equal to 2.9 and 3.9 Å for the minimum and maximum distances between any atoms in any residues, respectively [63,64]. Salt bridges between negatively and positively charged residues were identified following the criteria indicated in Ref. [65].

Similar analyses were also conducted on the 5 AF2 predicted structures of the EphA2-Sam/RNF5 complex and on the AF2 homodimeric models of the RNF5-PEP3 peptide and RNF5 protein.

### Peptides

The RNF5 peptides RNF5-PEP3 and RNF5-PEP2 in the N-terminal acetylated and C-terminal amidated forms were purchased from GenScript Biotech (Rijswijk, Netherlands) provided by DBA Italia, s.r.l. (Milan Italy). RNF5-PEP2 encompassing region 111 to 140 from entry Q99942 for human RNF5 was produced only as a crude peptide, while for RNF5-PEP3, encompassing a.a. sequence from 151 to 170, a purity of 94.4% was achieved (see analytical data in Figs. S2 and S3). Both peptides were subjected to 3 lyophilization cycles to remove as much TFA (trifluoroacetic acid) as possible.

### CD spectroscopy

Far-ultraviolet (UV) circular dichroism (CD) spectra (190 to 280 nm) were recorded at 25 °C on a Jasco J-1500 spectropolarimeter. Spectra were recorded using a 0.2-cm path length cuvette with a scan speed of 50 nm/min, a response time of 1 s, and a bandwidth of 1 nm.

Both RNF5-derived peptides, RNF5-PEP2 and RNF5-PEP3, were analyzed by CD spectroscopy. Peptides were initially dissolved in ultrapure water to obtain concentrated stock solutions and subsequently diluted to a final concentration of 100 μM in 10 mM phosphate buffer (pH 7.4), immediately prior to data acquisition. Duplicate measurements were collected to ensure reproducibility. Corresponding buffer spectra (blanks) were acquired under identical conditions and subtracted from the peptide spectra to remove background contributions.

The CD spectra were plotted as a mean ellipticity [*θ*] (degree × cm^2^ × dmol^−1^) versus wavelength *λ* (nm). Owing to its adequate purity and solubility, RNF5-PEP3 was subjected to detailed structural analysis. Prediction of structural states of RNF5-PEP3 peptide was performed by BeStSel software (https://bestsel.elte.hu/index.php) [66].

Additional CD experiments were carried out exclusively on RNF5-PEP3 to investigate its conformational behavior in the presence of increasing concentrations of 2,2,2-trifluoroethanol (TFE). The peptide was initially dissolved in aqueous buffer and diluted to the desired final concentration. TFE was added stepwise to the peptide solution to obtain final TFE concentrations of 0%, 10%, 30%, 50%, and 60% (v/v), while keeping the peptide concentration constant. Far-UV CD spectra were recorded in the 190 to 260 nm wavelength range using a quartz cuvette with a 0.2-cm path length at room temperature. Each spectrum represents the average of 2 accumulations. Buffer spectra containing the corresponding percentage of TFE were recorded separately and subtracted from the peptide spectra. CD data were converted to mean residue molar ellipticity prior to analysis. Secondary structure estimations were obtained by deconvolution of the CD spectra using the BeStSel algorithm, employing reference datasets appropriate for peptide and protein secondary structures.

### Protein expression

^15^N-labeled EphA2-Sam and Ship2-Sam were produced as recombinant proteins in *Escherichia coli* as previously reported [36,67].

### NMR spectroscopy

NMR studies were performed with a Bruker Avance 500-MHz spectrometer equipped with a cryoprobe at 298 K. A stock solution of the RNF5-PEP3 55 mM in dimethyl sulfoxide (DMSO)-d6 (99.9% D, Cambridge Isotope Laboratories) was used to obtain NMR samples with the following solvent conditions: 100% DMSO and PBS (phosphate buffer saline: 10 mM phosphates, 137 mM NaCl, and 2.7 mM KCl, from Sigma-Aldrich, Milan, Italy) pH 7.4 with 10% D_2_O (98% D, Sigma-Aldrich, Milan, Italy) (v/v). In addition, an RNF5-PEP3 sample (450 μM concentration) was prepared in PBS with 60% TFE (2,2,2-trifluoroethanol-d3, 99.5% isotopic purity, Sigma-Aldrich, Milan, Italy). All samples consisted of a total volume of 600 μl. Peptide concentrations were estimated based on weighted peptide amounts. NMR spectra registered for conformational studies of RNF5-PEP3 sample in 100% DMSO and in PBS/TFE 40/60 v/v consisted of 2D [^1^H, ^1^H] TOCSY (total correlation spectroscopy) [68] (70 ms mixing time), NOESY (nuclear Overhauser enhancement spectroscopy) [69] (300 ms mixing time), [^1^H, ^1^H] ROESY (rotating frame Overhauser enhancement spectroscopy) (250 ms mixing time) [70], and DQFCOSY [71] (double quantum-filtered correlated spectroscopy). For the peptide sample in PBS/TFE 40/60 v/v, 2D [^1^H,^15^N] and [^1^H,^13^C] HSQC (heteronuclear single quantum coherence) spectra were also acquired. Suppression of the water signal was obtained through excitation sculpting [72]. Chemical shifts were referenced to the water signal (4.7 parts per million [ppm]) for the RNF5-PEP3 samples in PBS/D_2_O/DMSO; for the RNF5-PEP3 peptide spectra acquired in 100% DMSO, the solvent peak at 2.5 ppm was used as reference; TSP (trimethylsilyl-3-propionic acid sodium salt-D4, 99% D, Armar Scientific, Switzerland) (0.0 ppm) was employed as internal standard for chemical shift referencing for RNF5-PEP3 experiments in PBS/TFE 40/60 v/v. The software to process and analyze the spectra included TopSpin 4.2 (Bruker, Milan, Italy) and NEASY [73] comprised in CARA (http://cara.nmr.ch/doku.php/). Chemical shift assignments were obtained through a standard protocol by comparison of TOCSY and NOESY spectra [74]. Hα proton chemical shift deviations with respect to predicted random coil values [Δδ(Hα) = δ(Hα)observed – δ(Ηα)random coil] were obtained through the method by Kjaergaard and Poulsen [75] applicable to assess random coil chemical shifts (https://www1.bio.ku.dk/english/research/bms/sbinlab/randomchemicalshifts1, accessed 2025 December 19). Δδ(Hα) values were used to evaluate the percentage of helical content with the equation: [ΔδHα_ave_/(−0.39)] × 100] where ΔδHα_ave_ represents the mean value calculated over residues associated with negative Δδ_Hα_ [76].

### NMR interaction studies

NMR binding experiments consisted of chemical shift perturbation assays through 1D [^1^H] and 2D [^1^H,^15^N] HSQC spectra. HSQC experiments were recorded for ^15^N-labeled EphA2-Sam (14.8 μM concentration) alone or in the presence of RNF5-PEP3 (45, 160, 250, and 330 μM final peptide concentrations obtained by diluting in PBS a 55 mM concentrated RNF5-PEP3 DMSO stock solution). A displacement experiment was similarly performed by registering a [^1^H,^15^N] HSQC spectrum after addition of unlabeled Ship2-Sam (45 and 108 μM concentrations) to the EphA2-Sam/RNF5-PEP3 complex. NMR interaction studies were also conducted for Ship2-Sam (14.8 μM concentration) in the absence and presence of RNF5-PEP3 (330 μM concentration). Reference spectra were acquired with ^15^N-labeled EphA2-Sam and ^15^N-labeled Ship2-Sam samples including proper amounts of DMSO. The pH of the protein and protein plus peptide samples was kept constant during binding studies and consequently adjusted with a NaOH solution when necessary. Reported peptide concentration values can be considered rough estimates due to poor peptide solubility.

### Microscale thermophoresis

Microscale thermophoresis (MST) experiments were carried out using a Monolith NT115 system (Nano Temper Technologies) with LED 100% and IR-laser 80% power. The EphA2-Sam and Ship2-Sam proteins were labeled with NT-647 reactive dyes (Nano Temper Technologies) by *N*-hydroxysuccinimide (NHS) ester chemistry. The protein concentration was adjusted to 15 μM with labeling buffer (Nano Temper Technologies), and the labeling reactions were performed by adding the dye in a molar ratio of 1:3 (45 μM). The labeling reaction was incubated for 60 min at room temperature in the dark. The peptide RNF5-PEP3 was analyzed in the range of the concentration 500 to 0.0076 μM by a 16-step serial dilution 1:2. Measurements were carried out at 25 °C, in PBS, DMSO 2%, and 0.05% Tween (pH 7.5) buffer using standard capillaries. The fit of the fraction bound values versus the peptide concentration was performed by using the software MO-S002 MO Affinity Analysis provided by the manufacturer.

### Aggregation propensity analysis

The 5 monomeric AF2 models of both RNF5 protein and RNF5-PEP3 peptide were used as input for the A3D (Aggregscan3D) 2.0 webserver (https://biocomp.chem.uw.edu.pl/A3D2/, accessed 2026 February 5) [77,78]. Predictions through the A3D 2.0 webserver were achieved by using default values for all parameters (i.e., “Stability calculations = Yes, “Dynamic mode” = No, “Mutate residues” = No, “Distance of aggregation analysis”= 10 Å, and “Enhance protein solubility” = No) [78].

The AggreProt webserver (https://loschmidt.chemi.muni.cz/aggreprot/, accessed 2026 February 5) was used for a further analysis on the aggregation propensity of RNF5 protein and RNF5-PEP3 peptide starting from the same AF2 models used as input for the A3D 2.0 webserver [79].

## Results and Discussion

### Preliminary RNF5 analyses: Looking for available experimental structures through the PDB databank

RNF5, also known as RMA1, is an ER-associated E3 ubiquitin ligase that, by adding ubiquitin chains on lysine residues, marks proteins for degradation. Ubiquitination and degradation taking place at the right time frame are essential for many regular cellular events. “RMA1” stands for “RING finger protein with a membrane anchor”; in fact, the corresponding cDNA was isolated for the first time in the *Arabidopsis thaliana* plant during a screening to discover molecules involved in the plant secretory pathway and codified for a protein containing a RING finger motif and a membrane binding region [48]. RMA1 is maintained from the plant *Arabidopsis* to human [48]. The RING domain (see the “The RING domain” section) in RMA1 is necessary for the E3 ubiquitin ligase activity. Three enzymes are crucial in the ubiquitin–proteasome pathway and need to work sequentially to achieve protein ubiquitination: first, the E1 ubiquitin-activating enzyme activates the ubiquitin and next transfers it to the E2 ubiquitin-conjugating enzyme; finally, through the support of the E3 ubiquitin-ligase enzyme that is involved in target protein recognition, E2 can transfer ubiquitin to specific substrates [80]. When the maltose-binding protein (MBP) is conjugated to RMA1 in both human and plant, the RING domain is able to auto polyubiquitinate the MBP, but poly-ubiquitination does not take place if free MBP and RMA1 are both present in solution, thus showing that the ubiquitin ligase action requires a substrate oriented in a proper manner and placed close to the RING domain. The Ubc (Ubiquitin-conjugating) 4/5 subgroup of E2 works with RNF5 E3 [48].

**Fig. 2.**
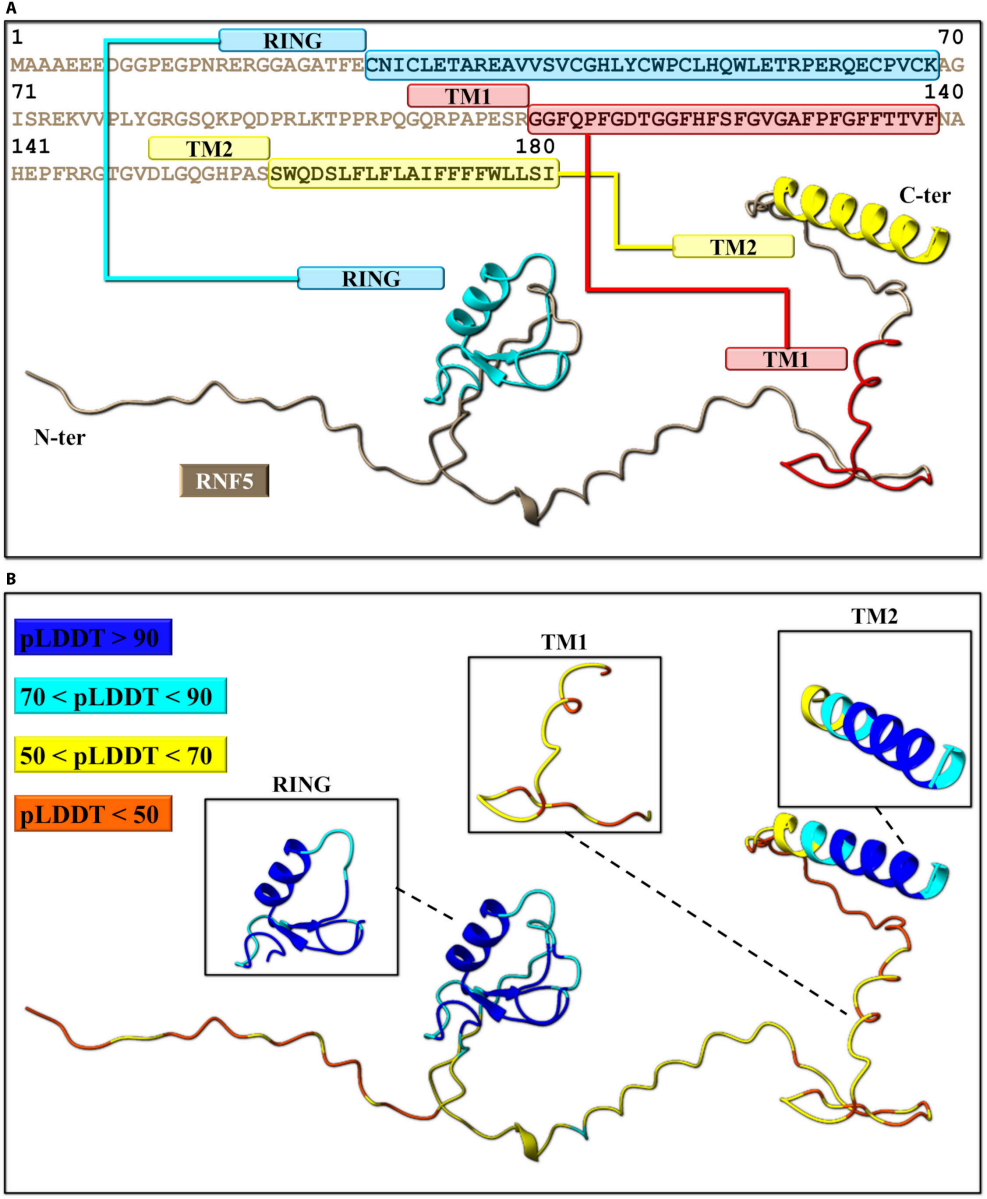
(A) The primary sequence of the human RNF5 protein (UniProt entry Q99942) is shown on top and the corresponding AlphaFold Model (ID: AF-Q99942-F1-v6) is shown at the bottom. The RING domain and TM1 and TM2 regions are colored cyan, red, and yellow, respectively. (B) AF model AF-Q99942-F1-v6. The atomic coordinates are represented in ribbon drawing with each amino acid colored according to the confidence level given by the pLDDT score (see the color legend on the top left side: blue stands for highly confident prediction, cyan denotes confident prediction, yellow indicates low accuracy prediction, and orange represents very low confident prediction [102]).

The human RNF5 protein is made up of 180 amino acid residues (Fig. 2). Diverse studies indicate within the human protein domain arrangement just the RING domain and a C-terminal transmembrane region [44,81,82] (encompassing residues from 160 to 180). However, based on the subcellular localization section of UniProt [57] (https://www.UniProt.org/UniProt/Q99942/entry#subcellular_location [83], accessed 2026 January 21), another transmembrane region might cover residues 118 to 138: we thus decided to indicate within the protein domain organization both TM regions (designed as TM1 and TM2 in Figs. 1 and 2) apart from the RING domain. The TM2 domain has been better characterized; it consists of a single transmembrane crossing domain that is crucial to engage RNF5 at the ER membrane while maintaining the RING domain exposed in the cell cytoplasm so that it can engage unfolded proteins or other specific substrates that need to be tagged with ubiquitin to be degraded [44,48,84].

A TM1 region and a TM2 region are also indicated within the domain organization of the black carp RNF5 [85].

We next manually inspected the PDB database looking for experimentally determined structures of human RNF5 focusing on the entire protein and single domains/functional regions; our search through the PDB included the following keywords: “RNF5”, “E3 ubiquitin-protein ligase RNF5”, and “RING finger protein 5”, but we could not retrieve any result. Thus, next, we used the amino acid sequence of different human RNF5 regions as a query for a BLAST(Basic Local Alignment Search Tool) search against the PDB database in order to conduct a more accurate analysis [86]. When using the primary sequence of the RING domain (Figs. 1 and 2, residues 27 to 68) as query, highest homology was gained by the E3 ubiquitin-protein ligase CBL (Casitas B-lineage lymphoma) with a 42.7% sequence identity (PDB entry: 5J3X [87]); for the TM1 region (residues 118 to 138, Figs. 1 and 2), the highest homology is provided by the membrane copper amine oxidase with a query coverage equal to 52% and a related identity equal to 87.5% (PDB entry: 2C10 [88]); finally, for TM2 (residues 160 to 180, Figs. 1 and 2), a good alignment is produced with the Carboxypeptidase M with a query coverage equal to 48% and an identity equal to 64.29% (PDB entry: 1UWY[89]). Our investigation demonstrates that the structure information on RNF5 is poor and no experimental structure of the diverse protein domains is available thus far.

A Computed Structure Model (CSM) of the full-length RNF5 protein could be retrieved from the AlphaFold Protein Structure Database (AlphaFold DB) (https://alphafold.ebi.ac.uk/) that has also been integrated into the PDB databank (Fig. 2) [90–93]. However, this model has an average pLDDT score [94], related to the confidence in the local structure, equal to 67.56, which points out the nonoptimal quality of the prediction. This is not surprising if one considers the elevated degree of disorder in certain protein regions that can be seen within the AlphaFold (AF) predicted RNF5 model (Fig. 2B).

### AF studies: Predicted structure models for isolated RNF5 regions

Our PDB analysis and Blastp [86] search point out that there are no available experimental structures of full-length RNF5 or its diverse domains/functional regions, so we decided to conduct more deep structural studies with AF to generate structure models of isolated protein regions. Indeed, lately, the artificial intelligence (AI) relying on tool AF has revolutionized the structure biology field by allowing to predict the structure of biological macromolecules in their free forms or even bound to diverse substrates with unprecedented accuracy [51,52,90,95]. We first employed AF to generate 3D coordinates for the isolated RNF5 RING domain and the putative transmembrane regions TM1 and TM2 (Fig. 1). Moreover, although as previously specified, an AF model of full-length RNF5 is available through the public repository AF database (Fig. 2), in order to be able to conduct accurate structure comparisons by using sets of 3D coordinates generated by employing the same computer resources and identical protocols (see Materials and Methods for details) without introducing methodological biases, we generated our own AF coordinates for the whole RNF5 protein. In addition, to get the highest level of information, structure predictions were carried out with both AF2 [96] and the newly released version AF3 [52]. Practically, AF2 relies on deep-learning and template-based methods [49,96]; an innovative algorithm was introduced in AF3 relying on a diffusion-based model [51,52] that improves its capacity not only to forecast isolated protein structures but also to handle complex interactions involving proteins, nucleic acids, small molecules, ions, and modified residues.

Regarding protein monomers, AF3 shows enhanced local structural accuracy with respect to AF2, although global accuracy benefits are restricted as both AF models have already reached a very high accuracy level. This means that no large differences are encountered in terms of TM (template modeling) scores but AF3 comes with a statistically significant advance with respect to AF2 concerning LDDT (local distance difference test) scores, especially when dealing with multidomain proteins. Nevertheless, AF2 and AF3 present diverse accuracy especially when handling easy targets with an improvement from ∼20% (AF2) to 30% (AF3) .

In general, AF3 [95,97] tends to simplify MSA (multisequence alignment) processing and presents a reduced dependence on MSA information, thus reaching better accuracy than AF2 when both running in the single-sequence mode [98]. In the case of orphan proteins that have reduced MSAs, a small and statistically meaningful gain in terms of local accuracy of the structure prediction can be reached with AF3. Guessing diverse conformational states, when dealing with multi-conformation proteins, is still a great challenge for AF3 and, although it performs better than AF2 for this specific class of biomacromolecules, still necessitates a lot of improvements to generate highly reliable results when handling systems characterized by conformational variability.

#### The RING domain

**Fig. 3.**
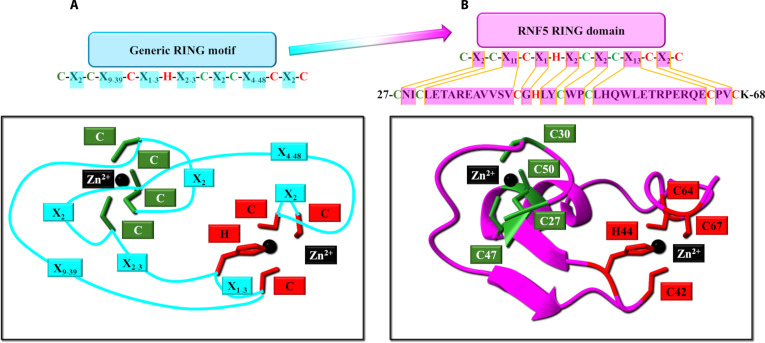
(A) Amino acid motif characteristic of a RING domain including HC3 and C4 Zn^2+^ coordination clusters; X and N stand for any amino acid residue and for the relative number, respectively. Residues of the HC3 cluster are shown in red while the green color refers to those of the C4 cluster. The amino acids positioned in between the 2 clusters are highlighted in cyan. A graphical representation of the tetrahedral coordination characterizing the RING domain motif is reported on the bottom side. (B) The RING domain motif from the human RNF5 protein (residues C27-K68 from the UniProt entry Q99942). The HC3 cluster (i.e., a.a. H44, C42, C64, and C67) is colored red while green is used for the C4 cluster (i.e., a.a. C27, C30, C47, and C50). In the sequence on the top, X indicates a generic residue while N denotes the number of generic residues. In the lower panel, the AF3 best predicted model of the human RNF5 RING domain (residues C27-K68 from the UniProt entry Q99942) is reported with highlighted HC3 (red) and C4 (green) Zn^2+^ coordination clusters.

The RNF5 RING domain contains a RING finger motif (Fig. 3) that is responsible for the E3 ubiquitin ligase activity, as clearly indicated by mutagenesis studies [48]. The RING finger motif of RNF5 includes 2 zinc ions in tetrahedral coordination with one metal coordinating cluster of the type C4 (i.e., involving 4 cysteines) and a second one of the HC3 type (i.e., involving 1 histidine and 3 cysteine residues) (Fig. 3).

**Fig. 4.**
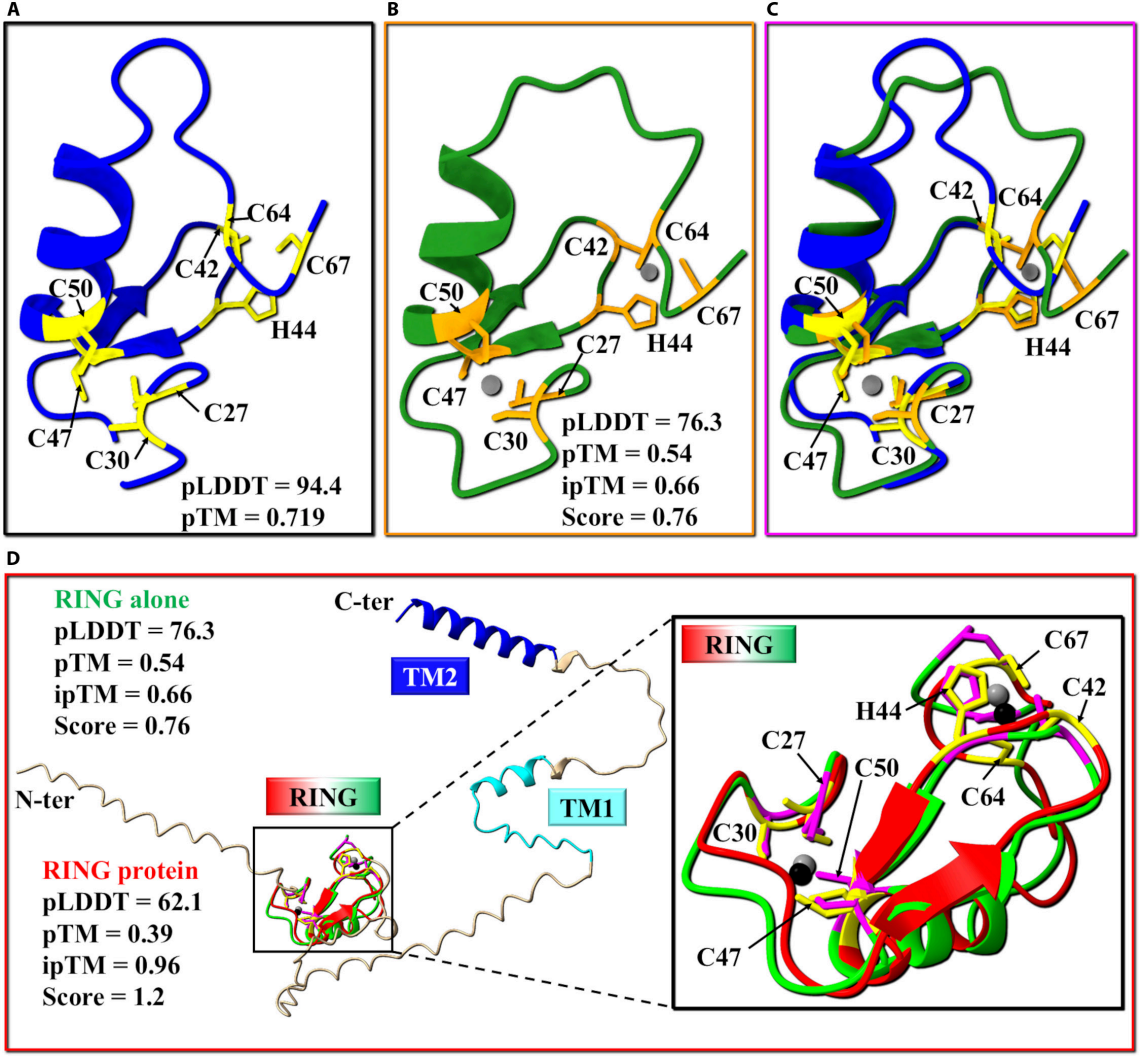
(A) AF2 [49,55] best model of the RING domain region of the RNF5 protein (residues C27-K68 from UniProt entry Q99942). The RING domain is shown in a ribbon representation colored blue with residues participating in the 2 Zn^2+^ ion coordination clusters HC3 (i.e., H44, C42, C64, and C67) and C4 (i.e., C27, C30, C47, and C50) highlighted in yellow with the side chains in neon representation (heavy atoms only). (B) AF3 [51,52] best model of the RNF5 RING domain (residues C27-K68 from UniProt entry Q99942) including the 2 Zn^2+^ ions (gray). The RING domain is shown in green with the residues contributing to the 2 Zn^2+^ coordination clusters colored gold. The structures in (A) and (B) are overlaid on the Cα atoms in (C); the superposition was generated with Chimera X version 1.5 [60]. (D) Comparison of best AF3 [51,52] models predicted in the presence of 2 Zn^2+^ ions for the entire RNF5 protein and the isolated RING domain. Cα atoms of residues from C27 to K68 have been superimposed with Chimera X version 1.5 [60]. The black rectangle includes the zoomed-in view of the superimposed RING domain regions; the green domain was extrapolated from the entire protein model while the red one derives from the isolated domain prediction. The residues contributing to the 2 Zn^2+^ coordination clusters of the RING domain alone and within the entire protein are shown in magenta and yellow, respectively. Zn^2+^ ions are colored black when inserted in the entire protein, and gray when in the isolated RING domain. The TM2 region (a.a. S160-I180) is colored blue and the TM1 one (a.a. G118-F138) is colored cyan. The pLDDT, pTM, and ipTM scores, along with the AF3 ranking scores, used to sort the 5 best models [51,52,107], are indicated.

As mentioned before, we predicted the structure (i.e., 5 best models) of the RING domain of RNF5 with both AF2 [49,55] and AF3 [51,52] (Fig. 4 and Fig. S4). In addition, the AF3 model of the whole RNF5 protein (residues 1 to 180) was generated and the atomic coordinates corresponding to the RING domain (i.e., residues 27 to 68) extracted from there and compared with those generated for the isolated domain (residues 27 to 68) (Fig. 4D and Fig. S5). Interestingly, differently from AF2, AF3 allows incorporating within predicted models the 2 zinc ions. In the case of AF3, the 5 best models were ranked based on the AF3 ranking score, which is calculated according to the equation reported in Materials and Methods [51,52], whereas ranking of the 5 best AF2 predictions follows the pLDDT values (Fig. 4A and B and Fig. S4). AF predictions (Fig. 4 and Figs. S4 and S5) closely resemble the structures of other RING domains contained in the PDB (i.e., PDB entry 2ECJ corresponding to the RING domain of the human tripartite motif-containing protein 39 [99]; PDB entry 6FGA referring to TRIM21 E3 ligase, RING domain in complex with its cognate E2 conjugating enzyme UBE2E1 [100]; PDB entry 4AP4 referring to the dimeric RING domain of rat RNF4 in complex with E2 [UbcH5A] [101]).

The folded region of each model includes an α-helix and a β-hairpin made up of 2 antiparallel β-strands (Fig. 4 and Figs. S4 and S5); regarding the first “C4” Zn coordinating cluster, C27 and C30 are positioned on the N-terminal tail that tends to form a noncanonical turn-like (“Bend”) structure similar to a β-hairpin, allowing them to face in the proper orientation the other 2 coordinating residues C47 and C50, which are instead positioned at the N-terminal region of the α-helix. Similarly, the second “HC3” cluster includes C42 and H44 that encompass the β-hairpin and C64 and C67 that are located instead within the C-terminal side within a structural arrangement resembling a turn (Fig. 4A to C and Figs. S4A and B and S5). In the AF2 models (Fig. 4A and Fig. S4A), the β-strands encompass residues A37-V39 and L45-C47 while the α-helix includes, in 2 out of 5 models, residues from W48 to E55 or from W48 to T56 (in 3/5 models). Similarly, in the AF3 predictions (Fig. 4B and Figs. S4B and S5), the β-strands encompass residues A37-V39 and L45-C47 (3/5 models) or V38-V39 and L45-Y46 (2/5 models), and the α-helix covers the W48-E56 (3/5 models) or W48-T57 (2/5 models) segments.

However, a detailed comparison of AF2 and AF3 [51,52,59] models was carried out, and as can be clearly seen in Fig. 4A to C and Fig. S4, no relevant differences can be evidenced as the overall RING domain fold is practically identical. The same can be further evidenced by the more accurate root mean square deviation (RMSD) analyses reported in Table S1 that pointed out the largest differences between corresponding AF2 and AF3 models within the most disordered regions of the domain that do not contain regular secondary structure elements.

To see if prediction of the isolated domain could somehow be different from the prediction AF could generate when using as input query the amino acid sequence of the entire RNF5 protein (i.e., residues 1 to 180, UniProt Entry Q99942), we compared the AF3 models predicted for the isolated domains (i.e., residues 27 to 68, UniProt Entry Q99942) with the corresponding region extrapolated from the AF3 prediction of the entire RNF5 protein (Fig. 4D, Fig. S5, and Table S2). This analysis was meant to see if considering the RING domain within the larger protein structural arrangement major differences in the fold, including the amount of ordered secondary structure elements, could be predicted by AF3 following, for example, interactions of the RING domain with other protein regions, but again, no major differences could be observed (Fig. 4D and Fig. S5) when the predicted models of the isolated RING domains were superimposed with those extrapolated from the entire protein. Indeed, even RMSD evaluations (Table S2) clearly demonstrate that the differences in the 2 sets of predicted models are effectively not drastic and approximatively of the same order of magnitude with respect to what was observed for comparison of AF2 and AF3 predictions of isolated domains (Table S1) and that differences can be mainly attributed to the most disordered loop regions connecting the ordered α- and β-secondary structure elements.

Thus, for the RNF5 RING domain, if we focus on the best predicted models (Fig. 4A and B), AF2 and AF3 pLDDT values are 94.4 (Fig. 4A) and 76.3 (Fig. 4B), respectively, indicating rather accurate predictions; the same occurs in the 4 other predicted models (Fig. S4). In fact, AF2 pLDDT values fall within the range 91 to 94 (Fig. S4A). Regarding AF3 models, as the pLDDT values are lower than 90 but still in the 70 to 90 range (Fig. S4B), the confidence level associated with the RNF5 RING domain models can still be considered rather good [102].

It can be noticed that despite a very similar fold, there is a difference in the pLDDT values when comparing models obtained with AF2 with those obtained with AF3; AF3 pLDDT scores tend to be lower. This trend has been observed in previous studies on different proteins [103,104] and is due of course to the different way exploited by AF3 to calculate the pLDDT values [51,52]. It is worth noting [51,52] that because the precision of AF3 is superior to that of AF2, lower scores are important to avoid misinterpretation, allowing the recognition of an incorrect fold. For example, based on differences in pLDDT values between AF2- and AF3-generated structure models, an interesting work described how AF2 could predict unrealistic highly confident β-solenoid structures for certain repeat proteins characterized by an intrinsic disordered nature [104].

Regarding AF3, ipTM values being lower than 0.8 might point out a certain ambiguity in the interaction with zinc ions and, thus, not a completely optimal positioning of the zinc ions within the 2 coordination clusters (Fig. 4B and Figs. S4B and S5) [102].

It is interesting also to note that the AF3 pLDDT values become lower when considering models predicted for the whole protein (Fig. 4D and Fig. S5) as there is clearly a contribution from the most disordered regions, outside the RING domain, that produces a decrease in the accuracy of predictions for the affected protein segments (Fig. S6). Indeed, inspection of UniProt entry Q99942 for human RNF5 reveals that the 79 to 110 protein segment could be considered a disordered region (https://www.UniProt.org/UniProtkb/Q99942/entry#family_and_domains, accessed 2026 February 25).

#### TM1 region

We next focused on the C-terminal transmembrane domain of the RNF5 protein. According to the data reported for the UniProt entry code Q99942, there are 2 putative transmembrane regions within the protein that should be provided with a helical arrangement (i.e., designed by us as TM1 and TM2 in Fig. 1) (https://www.UniProt.org/UniProtkb/Q99942/entry#subcellular_location, accessed 2026 February 25). To obtain further structure insights, we used AF to predict the structures not only of the isolated TM1 (residues 118 to 138) and TM2 (residues 160 to 180) segments but also of the extended region encompassing both TM1 and TM2 (residues 118 to 180). To the best of our knowledge, while the functional features related to the TM2 domain are better characterized, very little is reported in literature on this TM1 region and how it can be related to the RNF5 ubiquitin E3 ligase activity. Indeed, the TM1 region is often not even indicated within the domain organization of the protein.

However, in other ubiquitin E3 ligases containing TM1 and TM2 regions such as the MARCH (the Membrane-Associated RING-CH) group [105] and the mitochondrial ubiquitin ligases like RNF185 [106], the transmembrane regions play a crucial role for protein localization and substrate recognition and are thus important for the ubiquitination and down-regulation of protein substrates.

A study focused on bcRNF5 (i.e., black carpet RNF5) stressed out how the amino acid sequences of the RING domain and TM1 and TM2 regions in the fish are similar to those of mammals, birds, and zebrafish, pointing to function conservation of RNF5 in vertebrates [85].

**Fig. 5.**
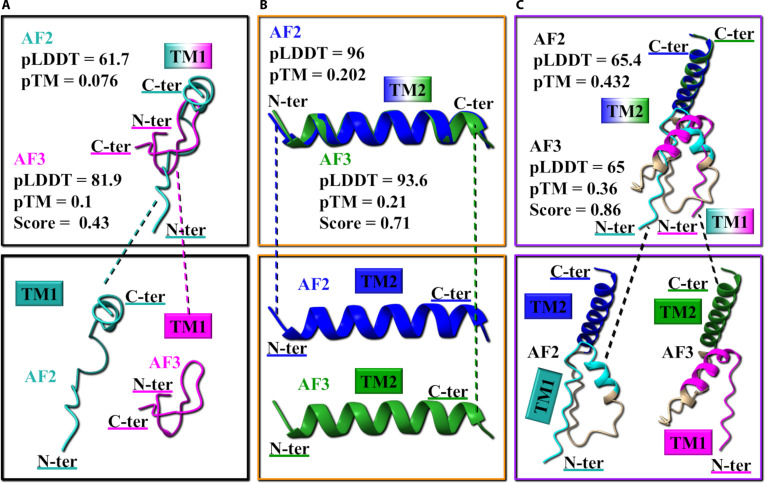
Comparison of best AF2 [49,55] and AF3 [51,52] models predicted for (A) the first transmembrane (TM1) region of the RNF5 protein (G118-F138, UniProt code Q99942), (B) the second transmembrane (TM2) region of the RNF5 protein (S160-I180, UniProt code Q99942), and (C) the G118-I180 region of RNF5 including the TM1 and the TM2 regions of RNF5. TM1 is colored light sea green in AF2 models and magenta in AF3 models (A and C), while TM2 is colored blue in AF2 models and green in AF3 models (B and C). The models have been overlaid by Chimera X version 1.5 [60] on the Cα atoms of all residues from the TM1 region (A), TM2 region (B), and TM1+TM2 region (C).

The 5 best AF2-predicted models of the RNF5 TM1 region are shown in Fig. 5A and Fig. S7A. Transmembrane regions in proteins have a generally helical organization; the AF2 models are characterized by a rather disordered structural arrangement that includes distorted helical turns. In fact, inspection of each model with the software MolMol [62] shows in the protein region encompassing residues G125-T136 a variable content of turn/bend, whereas only in the AF2 third- and fifth-ranked structural models could more regular short alpha helical segments be revealed (i.e., residues F121-V137 in Model 3 and F131-F134 in Model 5). AF2 prediction is characterized by low accuracy as indicated by the confidence scores (Fig. S7A and Fig. 5A) [49,59]. AF3 models (Fig. 5A and Fig. S7B) [51,52] of RNF5 TM1 appear very different from the AF2 structures (Fig. 5A, Fig. S7A, and Table S1). Analysis with MolMol [62] of AF3 best TM1 models indicates a reduced bend content and even the presence in one structure of a parallel 2-stranded β-sheet (fourth-ranked model). The local accuracy indicated by the pLDDT scores (Fig. 5A and Fig. S7B) [59,107,108] point toward a low confident prediction. For low-molecular-weight proteins and peptides, looking at pTM scores instead might generate confusion as such a parameter tends to be tiny for small structures and short chains; for example, values below 0.05 are assigned to systems made up of less than 20 amino acids, and cannot be considered to judge the quality of a structure prediction [102]. For these cases, predicted aligned error (PAE) and/or pLDDT may be more indicative of prediction accuracy.

Higher pLDDT scores are related to AF3 models of TM1 and point out a decent local confidence level, whereas the global accuracy related to pTM is still very low. Differences in AF2 and AF3 confidence scores, as mentioned in the “The RING domain” section, are not surprising as it has already been noticed in other studies that in between the 2 AF releases, scores and structure predictions can be different [103,104], and this might be due to the different ways the 2 AF predictors work and calculate the scores. As mentioned before, AF2 models are very different from the AF3 ones, and this generally should also be expected for intrinsically disordered protein regions that are still too challenging to be properly predicted by AF [109]. We thus employed the IUPred3 (Intrinsically Unstructured Prediction3) [110] webserver for disorder prediction (see Fig. S8). However, the amino acid sequence corresponding to the TM1 domain in RNF5 (Fig. 1) was predicted to be completely ordered by IUPred3 (Fig. S8). In the end, it can be realistically supposed that both AF2 and AF3 models of RNF5 TM1 domain are wrong as this region is particularly flexible and can assume the proper helical fold just following interaction with membranes or upon formation of homo- or heterodimers/oligomers that represent scenarios that cannot at this time be contemplated by AF predictions [109]. Another possibility is that AF2 and AF3 are failing to produce a proper helical conformation of the TM1 region as this is not a transmembrane domain. As specified before, the role of TM1 within RNF5 signaling has not been clarified thus far, to the best of our knowledge, and more experimental studies should be conducted to prove its membrane anchoring activity.

#### TM2 region

The C-terminal TM2 transmembrane domain supports many diverse cellular outcomes mediated by RNF5. For instance, it is important for RNF5 ER targeting and is involved in binding to diverse substrates such as MAVS (mitochondrial antiviral signaling protein), thus modulating antiviral innate immune answer [85], or paxillin, thus regulating cell migration [111]. It has been demonstrated that, *in vivo*, this membrane anchor site should be very important for proper subcellular localization and for substrate recognition [48].

The 5 best AF2 models representing the predicted structure of the RNF5 TM2 domain are presented in Fig. 5B and Fig. S9A. Prediction of disordered sequence stretches with the IUPred3 [110] webserver for disorder prediction (see Fig. S8) indicated a disordered score practically equal to zero and thus the global folding of the corresponding primary sequence (i.e., a.a. 160 to 180; see Fig. 1). The AF2 5 best models consist of an α-helix covering most of the region (i.e., from Trp161/Gln162 to Leu178/Ser179) as indicated by deep analyses with the software MolMol [62]. AF3 models appear very similar to the AF2 ones (Fig. 5B, Fig. S9B, and Table S1). The local pLDDT confidence scores for AF2 and AF3 models are high, around 90, and consequently indicate an accurate prediction; as specified before, pTM scores tend instead to be small for relatively short peptides and, consequently, cannot be considered hallmarks of low precision structure predictions. Interestingly, differently from the TM1 region here, similar structures and confidence scores by both AF2 and AF3 were obtained.

The TM2 AF structure appears in perfect agreement with results from bioinformatic tools for membrane protein topology prediction.

The webserver MembraneFold [112] was employed to predict the transmembrane protein topology (by DeepTMHMM [113]) of the entire RNF5 protein (Fig. S10) and the RNF5 TM2 region (Fig. S11). The input consists in the primary sequence of RNF5 protein (residues M1-I180) and RNF5 TM2 (residues S160-I180). The “Run OmegaFold” was selected in both cases if there is not an exact match in the “Alphafold Database” option.

The server MembraneFold identified the region L165-L178 as located within the membrane (Fig. S10A and B) with a transmembrane (TM) helical topology (Fig. S10B).

In agreement with these data obtained for the whole RNF5 sequence, a MembraneFold run conducted for just the TM2 region pointed out the segment L165-S179 as a helical TM region (Fig. S11).

Similarly, analysis of the RNF5 primary sequence with the Δ*G* prediction server [114] indicated 2 regions as putative TM helices: the F120-F138 stretch, included in the TM1 region, with Δ*G*_app_ (apparent) =1.973 kcal/mol and the D164-I180 portion, included in the RNF5 TM2 region, with Δ*G*_app_ = −2.571 kcal/mol. As also evidenced in Fig. S12, the stretch included in the TM2 region is that associated with the most negative Δ*G*_app_ and thus is the RNF5 portion with the highest chance to be a TM helix. Results of these predictors related to the TM1 corresponding region (i.e., a.a. from G118 to F138) along with AF structure predictions (see Fig. 5A and C) suggest that this segment might not indeed be related to a transmembrane helical topology at difference from TM2 that instead is always predicted, even in a nonmembrane-like environment, to assume a helical structuration similar to a canonical transmembrane domain.

#### Extended TM1 plus TM2 amino acid stretch

AF2 [49,55] and AF3 [51,52] models were also predicted for the a.a. region including both the TM1 and TM2 domains (Fig. 1). Through AF studies of this longer amino acid sequence, i.e., residues 118 to 180, we wanted to verify if the protein region corresponding to TM1 could assume a more organized helical fold, resembling that of the transmembrane regions, following possible interactions with other close protein segments including the TM2, with respect to what was observed with the isolated 118 to 138 TM1 amino acid sequence.

The best AF2 and AF3 predicted models are reported in Fig. 5C and Fig. S13. The best AF2 model includes, as indicated by secondary structure analyses with the software MolMol [62], an α-helix covering residues from G132 to A140 and thus only including the C-terminal region of TM1 and a second α-helix spanning residues from S160 to S179 corresponding to the TM2 domain (Fig. 5C). The AF3 best model is made up instead of an α-helix encompassing residues from V126 to H141 in the TM1 region while the TM2 region includes an α-helix covering residues from F159 to A179 (Fig. 5C). To better compare AF2 and AF3 predictions for the TM1+TM2 region, the best AF2 and AF3 models were superimposed on the backbone atoms, as indicated in the caption of Table S1. Visual inspection of the resultant overlay along with the high RMSD value (i.e., 10.788 Å) (Fig. 5C and Table S1) indicate major differences. Differences are mainly evident in the regions corresponding to the TM1 domain and correlate with the different amounts of secondary structure elements contained within these domains in the AF2 and AF3 models (Fig. 5C). Moreover, additional differences among the two models are due to the dissimilar orientations of the 2 TM1 and TM2 regions with respect to each other (Fig. 5C). The observed differences between best AF2 and AF3 models are caused by the AF intrinsic limitation of being unable, like most of the other structure prediction tools, to precisely model disordered regions like the loops in between the 2 major helical segments.

Comparative analyses of all the other best structures generated by AF2 and AF3 (Fig. S13) for the RNF5 118 to 180 region again indicate differences mainly positioned within the 118 to 138 region including the TM1 domain and in the disordered linker region (i.e., a.a. 139 to 159) while the TM2 160 to 180 site always appears as an α-helical domain in both AF2 and AF3 models (Fig. S13). AF confidence scores and mainly the pLDDT values are similar for AF2- and AF3-generated models (Fig. S13 and Fig. 5C) and scores around 60 are associated to a low confident prediction. In agreement with what was observed during our studies with the isolated TM1 and TM2 domains, the prediction precision is lowered by the TM1 region that appears very challenging to be modeled by AF. If these difficulties are related to a certain intrinsically disordered nature, which common bioinformatic tools like IuPred3 [110] fail to recognize, or to a conformational variability and a certain dynamism, this needs to be determined by experimental studies.

### AF structure predictions to investigate the interaction between EphA2 and RNF5

As mentioned in the Introduction, experimental *in vitro* studies (=co-immunoprecipitation coupled to mutagenesis analyses with truncated protein constructs) indicate that the interaction between EphA2 and RNF5 exploits the Sam domain of EphA2 and the TM2 domain of RNF5 [43]. However, to get deeper insights into structural features governing molecular recognition processes regarding EphA2 and RNF5, rather than focus just on modeling the EphA2-Sam/RNF5 TM2 complex, we explored the probability of occurrence of interactions in between EphA2-Sam and all diverse RNF5 regions (i.e., TM1, TM2, and RING) firstly by employing structure predictions by AF2 [49,50] and AF3 [51,52]. Moreover, we also modeled the interaction of the whole RNF5 protein with EphA2-Sam. Understanding indeed if additional RNF5 domains/regions exhibit a tendency to interact with EphA2-Sam might help unveil possible structural rearrangements that might accompany the formation of the EphA2-Sam/RNF5 TM2 complex. Our approach is also intended to better understand how AF confidence scores should be interpreted to get results as much as possible in agreement with experimental data and eventually build, based on that, a general *in silico* protocol applicable to any PPI to reliably guess which protein regions should directly contact each other.

#### Modeling the entire RNF5 protein in complex with EphA2-Sam

**Fig. 6.**
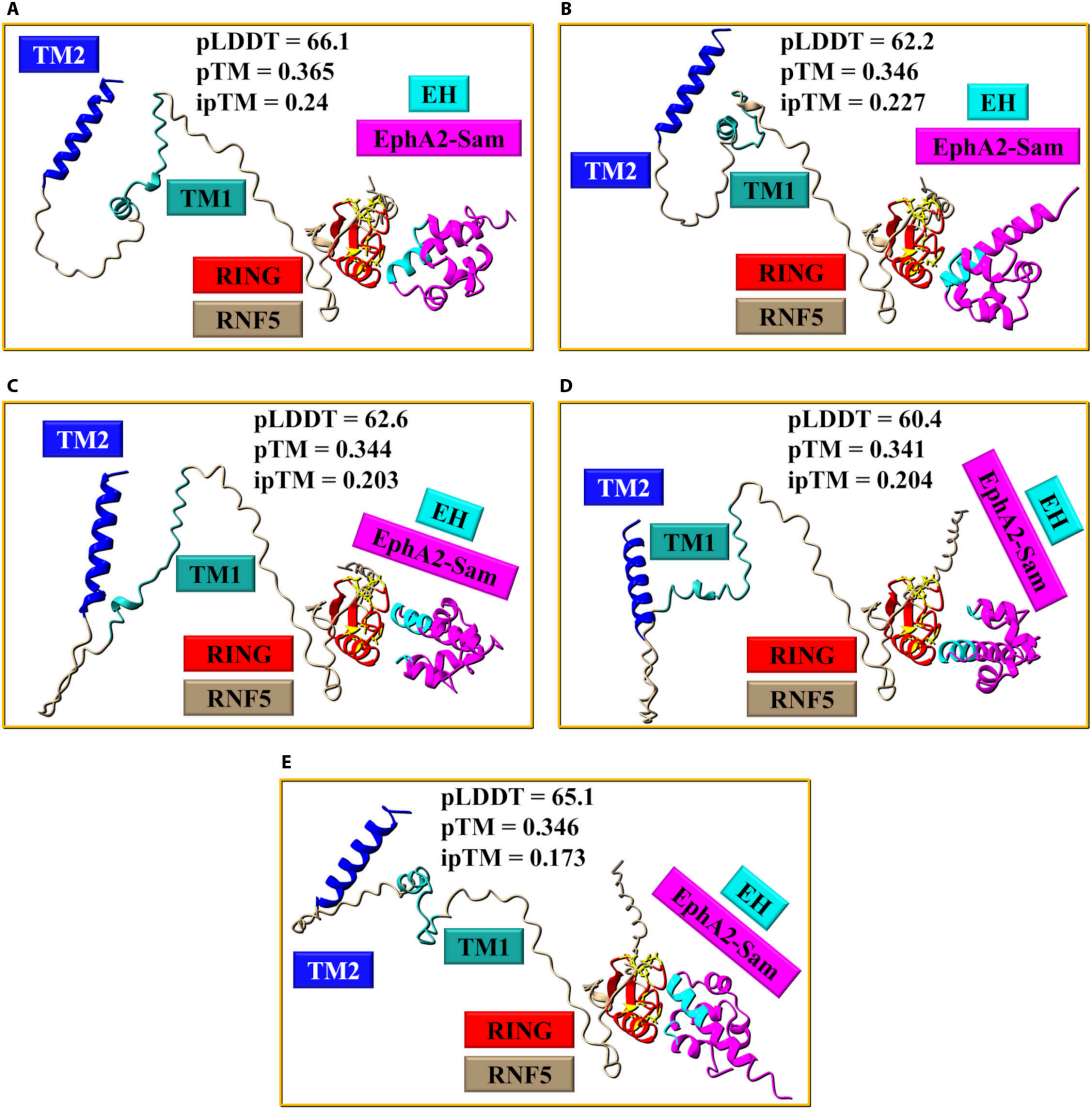
(A to E) Best AF2 [49,55] models of EphA2-Sam (residues V904-I976 including the PBM from the UniProt entry P29317) in complex with the entire RNF5 protein (UniProt entry Q99942). In each panel, EphA2-Sam is shown in magenta, with the EH region (residues I916-M918 and P952-Y960) highlighted in cyan. The RING domain (residue range C27-K68 from the UniProt entry Q99942) is colored red and yellow. The yellow color represents the residues contributing to the 2 Zn^2+^ coordinating clusters HC3 (i.e., H44, C42, C64, and C67) and C4 (i.e., C27, C30, C47, and C50). The TM1 (residues G118-F138 from the UniProt entry Q99942) and TM2 (residues S160-I180 from UniProt entry Q99942) of the RNF5 protein are shown in light sea green and blue, respectively. The AF2 confidence scores (i.e., pLDDT, pTM, and ipTM) are indicated for each model in the associated panel [59].

The amino acid sequence of the entire human RNF5 protein (residues M1-I180 UniProt entry Q99942) was submitted as input query along with the primary sequence of EphA2-Sam (residues 904 to 976 from UniProt entry P29317 for human EphA2, encompassing the Sam domain residues from V904 to Q968) to the ColabFold server multimer module [50,56] to get AF2 structure prediction of the EphA2-Sam/RNF5 complex. Interestingly, in all the 5 best AF2 models, EphA2-Sam is engaged by RNF5 through the RING domain (Fig. 6 and Table S3) and indeed analyses of intermolecular contacts (Table S3 and Fig. S14) clearly indicate that just a few residues from the RING domain, encompassing the RNF5 region from a.a. 27 to 68 (Fig. 1), provide in all 5 AF2 models interactions with EphA2-Sam (Fig. S14 and Table S3). Remarkably, the region of EphA2-Sam involved in binding to the RING domain of RNF5 includes the EH interface (Fig. S1, Table S3, and Fig. S14) that is positioned between the α5 C-terminal helix and the α1α2 loop and represents an important receptor site as involved into binding to partner Sam domains [36,37,41,115]. Analysis of diagrams of intermolecular contacts, generated with the software LigPlot+ [63,64] (Fig. S14), indicates that several EphA2-Sam residues, which, according to experimental structural and mutagenesis studies [37,41,115,116], are known to be involved in key interactions with partner Sam domains, might contribute contacts with the RNF5 RING domain in the complex between the whole RNF5 protein and EphA2-Sam (Table S3 and Fig. S14). These EphA2-Sam residues include K917, G953, K956, and R957 (Fig. S14). It is worth highlighting the involvement of G953 (Fig. S1) that is known to be essential to engage the ML site of EphA2-Sam interactors, which bind to the receptor through the ML/EH structural interaction topology. That said, it is normal that a putative engagement of RNF5 RING to EphA2-Sam would hamper the formation of EphA2-Sam-mediated Sam–Sam interactions, like the Ship2-Sam/EphA2-Sam complex [27,36]. Both RNF5 and Ship2 work as regulators of receptor stability, but in different opposite ways. Ship2 by inhibiting EphA2 endocytosis and degradation likely up-regulates receptor levels and the pro-oncogenic signaling [41], whereas RNF5, through ubiquitination [43], should down-regulate receptor levels but generating in this case in certain breast cancer cells pro-oncogenic outcomes witnessed by dysregulation of tyrosine/serine EphA2 phosphorylation levels. Thus, it can be speculated that, depending on the cancer cell type, a fine-tuning of Ship2 and/or RNF5 levels might induce a switch in EphA2 signaling, changing EphA2-Sam’s role from suppressing tumors to promoting them.

For multimers, the ipTM is another important ranking score related to the accuracy of the predicted relative positions of the entities involved in a complex (ipTM values > 0.8 indicate confident accurate predictions, values lower than 0.6 point to inaccurate predictions, and ipTMs in the 0.6 to 0.8 range are ambiguous and cannot be considered to draw any conclusion) [97]. IpTM and pTM scores for all models are low, and this again seems to support the low accuracy of structure predictions, although the disorder of certain RNF5 protein regions might negatively impact pLDDT values or even ipTM scores [102].

PAE is however another AlphaFold Confidence score that should be kept into account especially when dealing with very low ipTM values. PAE can be exploited to evaluate the accuracy in the domain packaging and relative positioning of single entities forming a complex. Low PAE scores indicate low errors and, consequently, high accuracy in predicting the relative positions of each entity, whereas high PAE scores are associated with high errors and, therefore, low accuracy [117,118]. In the case of the AF2 models of the EphA2-Sam/RNF5 protein complex, the PAE values in the on-diagonal regions suggest a well-folded structure for EphA2-Sam and an accurate prediction mainly for the fold of the RING domain of the RNF5 protein. Focusing on the off-diagonal regions, PAE values indicate a certain flexibility grade of the complex although the RING region appears characterized by better scores, thus indicating a largest accuracy of the AF prediction regarding the orientation of the RING domain with respect to the Sam domain.

In the end, considering AF results, it might be as well pointed out that the RING domain of RNF5 might indeed first interact with EphA2-Sam, creating a sort of inactive state; afterwards, upon a conformational transition, changes in the interaction networks might occur and EphA2-Sam could be engaged by the TM2 for ER targeting. Consequently, the RING domain is exposed in a sort of “active” form and correctly oriented for efficient ubiquitin transfer. This scenario, in the absence of experimental validation, remains just a plausible fascinating speculation that however seems well supported by AF results.

**Fig. 7.**
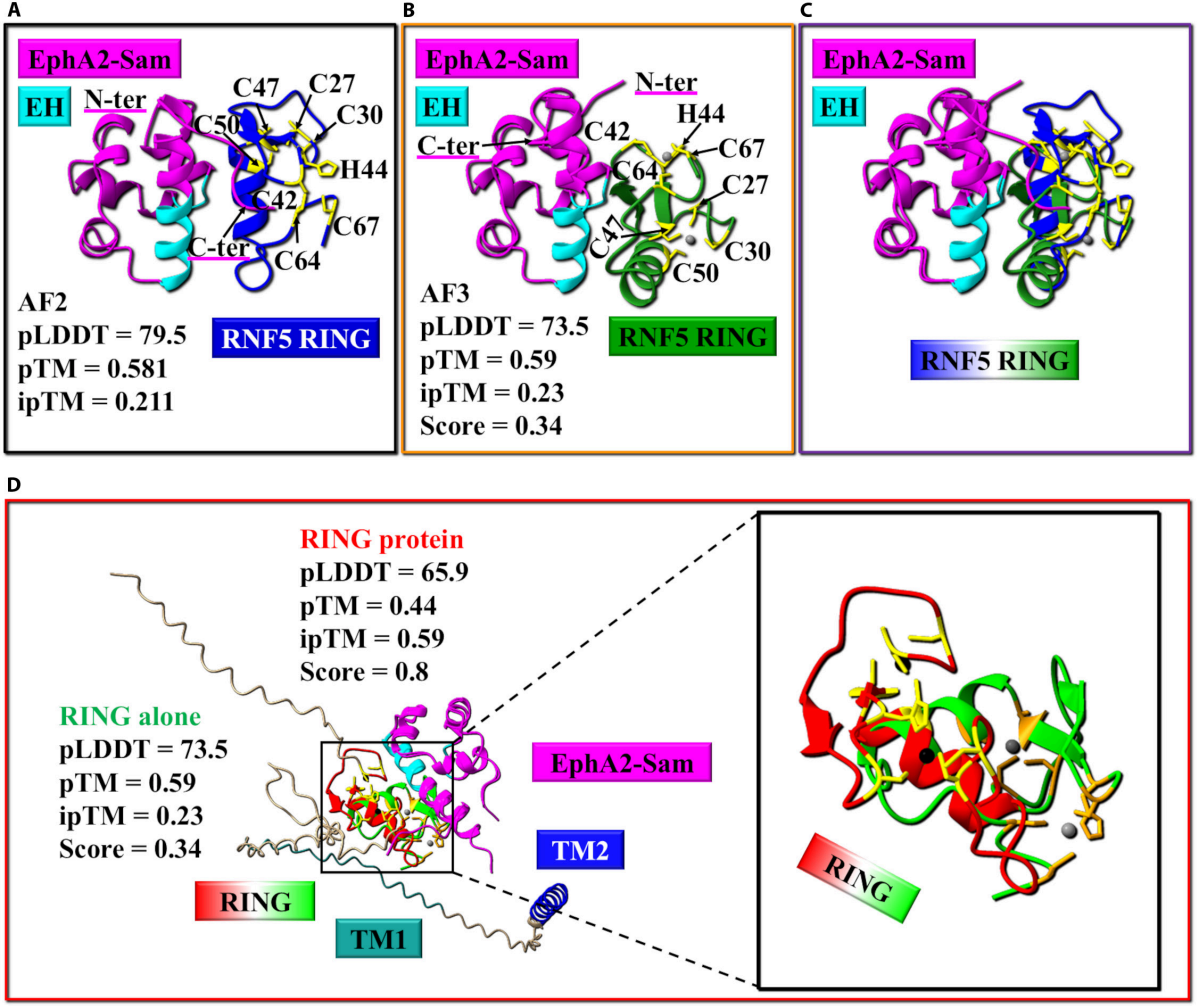
(A) AF2 [49,55] best model for EphA2-Sam (residues V904-I976 from the UniProt entry P29317) in complex with the RING region of the RNF5 protein (residues C27-K68, UniProt entry Q99942). EphA2-Sam is shown in a ribbon representation (magenta) with the EH region colored cyan (residues I916-M918 and P952-Y960); the RNF5 RING domain is presented in blue. (B) AF3 [51,52] best model for EphA2-Sam in complex with the RING region (green) of the RNF5 protein containing the 2 coordinating Zn^2+^ ions. Yellow is employed in panels (A) and (B) to highlight the residues contributing to the HC3 (i.e., H44, C42, C64, and C67) and C4 (i.e., C27, C30, C47, and C50) Zn^2+^ coordination clusters. (C) Superpositions on the Cα atoms of EphA2-Sam of the 2 complexes shown in (A) and (B), respectively. (D) Comparison (i.e., overlay on the Cα atoms of EphA2-Sam) of the best models predicted by AF3 [51,52] for EphA2-Sam in complex with either the isolated RING region of the RNF5 protein (residues C27-K68 from UniProt entry code Q99942) (light green) or the entire RNF5 protein (red). The residues contributing to the Zn^2+^ coordination clusters of the RING as the isolated region are colored orange with Zn^2+^ ions in gray and yellow with Zn^2+^ ions in black in the RING included in the whole RNF5 protein. In panels (B) to (D), EphA2-Sam is colored as in (A). The first (TM1 residues G118-F138 from UniProt entry Q99942) and the second transmembrane (TM2 residues S160-I180 from UniProt entry Q99942) regions of the RNF5 protein are presented in light sea green and blue, respectively. The zoomed-in view of the superimposed RING regions is reported in the black rectangle in panel (D). The superpositions in (C) and (D) were obtained with Chimera X version 1.5 [60]. In all panels pLDDT, pTM and ipTM scores of the best models are indicated along with the AF3 ranking scores [52,59,107,108].

Similar results can be obtained by AF3 [51,52] studies of the whole RNF5 protein in complex with EphA2-Sam showing again a predicted engagement of the EH interface in EphA2-Sam by the RING domain (see Fig. 7 and the “Predicting the structure of the isolated RNF5 RING domain in complex with EphA2-Sam” section) of RNF5.

#### Predicting the structure of the isolated RNF5 RING domain in complex with EphA2-Sam

To get further insights into a possible engagement of EphA2-Sam by the RING domain of RNF5, AF2 [49,55] and AF3 [52] analyses were conducted to predict the structure of the EphA2-Sam/RNF5 RING complex (Fig. 7 and Fig. S16).

Overlay on the backbone atoms of all residues of the EphA2-Sam/RNF5 RING best structures shows remarkable differences (Fig. 7C) further witnessed by a rather high RMSD value (Table S4). This outcome seems possibly related to the low ipTM scores, indicating the poor accuracy of AF2 and AF3 predictions in correctly positioning EphA2-Sam and RNF5 RING one with respect to the other in the predicted complex structure (Fig. 7 and Fig. S16). The AF2 PAE scores were evaluated as well (see Fig. S15) and indicate that the fold of isolated EphA2-Sam and the RNF5 RING domain are rather accurate (see on-diagonal squares in the second row of Fig. S15) in all predicted models, but the orientational variability in between the 2 chains is again evidenced by the nonoptimal and diverse PAE values characterizing the off-diagonal rectangles in each one of the 5 best models (Fig. S15).

The same “orientational” differences are evident when comparing the predicted structure of the EphA2-Sam/RNF5 and EphA2-Sam/RNF5 RING complexes (Fig. 7D, Fig. S17, and Table S5).

It is worth noting that in spite of the orientational variability, the EH interface of EphA2-Sam is mostly involved into interactions with RNF5 RING in all AF generated models presented in Fig. 7 and Figs. S16 and S17; this appears to highlight how the EH site could be reputed as a key binding interface generally employed by EphA2-Sam for molecular recognition.

There is a clear trend of AF, when modeling the interaction between the whole RNF5 protein and EphA2-Sam, to favor the RNF5 RING as an EphA2-Sam binding partner rather than the TM2 region, which, according instead to the experimental evidence, should represent the true interaction site. To better address this point, we analyzed by AF structural predictions also the interaction between EphA2-Sam and the transmembrane regions (TM1 and TM2).

#### Investigating by AF the interaction in between EphA2-Sam and the RNF5 transmembrane domain

To achieve this analysis, we adopted the same protocol employed for the isolated protein regions, so we focused first on the TM1 RNF5 site (residues G118-F138 Fig. 1), which could represent another transmembrane or more generally membrane anchoring domain [85]. Surprisingly, rather than a helical structuration that more often characterizes a membrane anchoring domain, this RNF5 protein region is predicted mainly as disordered by AF2 and AF3 that generate among the group of 5 best models very diverse and not converging conformers (Fig. 5A, Fig. S7, and Table S1). Given the AF results, we believe that more functional and structural/activity relationship data should be collected for the RNF5 segment corresponding to TM1 to validate a membrane anchoring activity and its meaning in association with the RNF5 ubiquitin ligase activity. TM2 is instead an established membrane anchoring domain [48,85,111], encompassing the more C-terminal RNF5 region (residues S160-I180) whose structure is predicted by both AF2 and AF3 as a canonical helical transmembrane domain (Fig. 5B, Fig. S9, and Table S1). Finally, we analyzed the TM1+TM2 region comprehending the RNF5 G118-I180 C-terminal tail. Within this longer protein fragment, AF2 and AF3 are able to predict a structural organization alternating helical segments to disordered interhelical loops with an increase of helical content in the region encompassing TM1 (i.e., a.a. 118 to 140) with respect to the isolated TM1 region, while the RNF5 fragment corresponding to TM2 remain in a rather order helical organization with high convergence among generated AF2 and AF3 best models (Fig. 5C and Fig. S13). In contrast, regarding the TM1 segment when joint to the TM2 site, large differences and poor convergence are observed among the 5 best AF2 and AF3 models in the segment between G118 and S159 regarding both the amount of helical content and the orientation of the diverse interhelical loops (Table S1, Fig. 5C, and Fig. S13).

These predicted structural features characterizing the TM1 and TM2 site in isolation or as a joint segment are maintained when complexes with EphA2-Sam are generated.

##### AF predicted model of the RNF5 TM1/EphA2-Sam complex

**Fig. 8.**
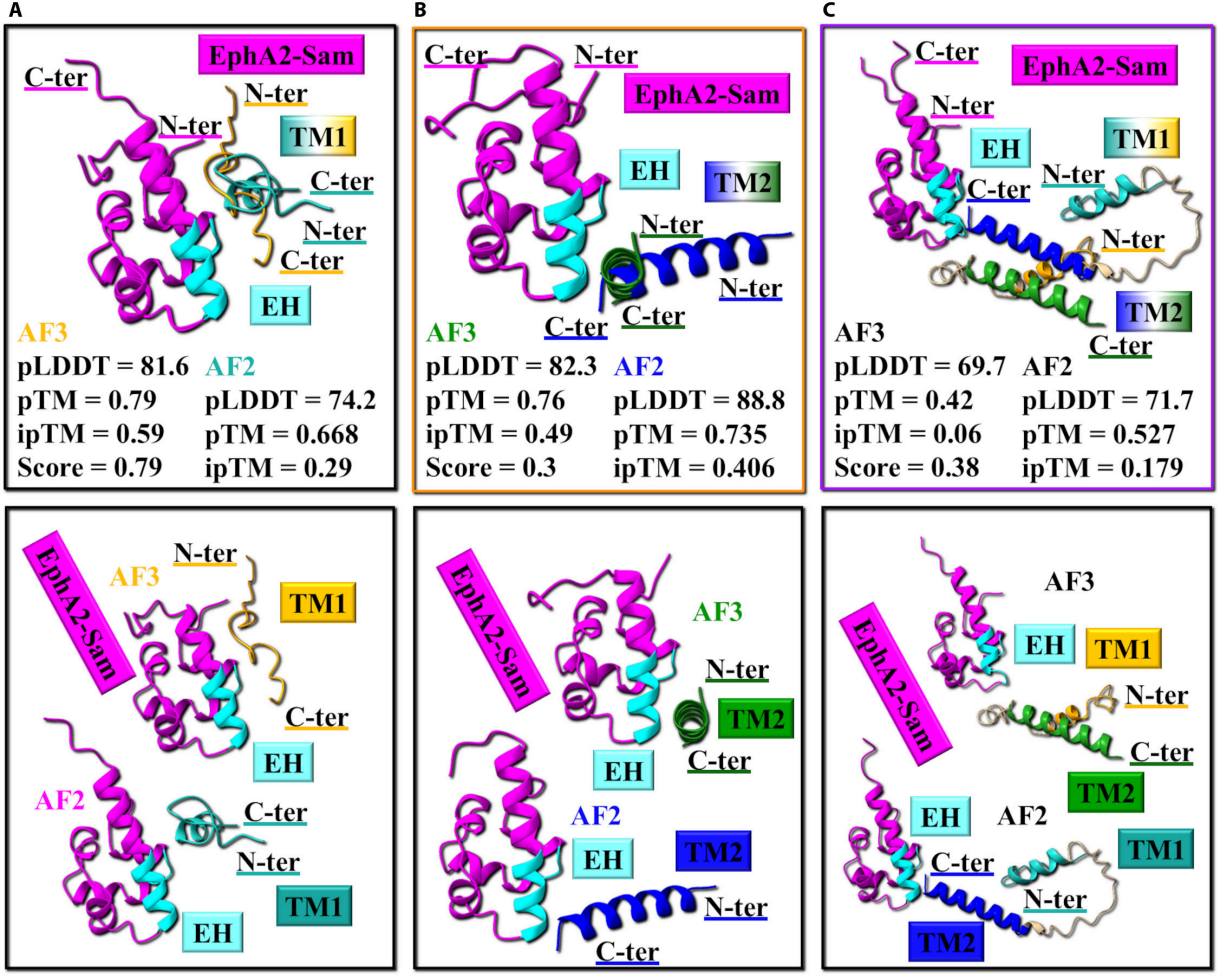
Comparison of AF2 [49,55] and AF3 [51,52] best models for EphA2-Sam (residues V904-I976 from the UniProt code P29317) in complex with (A) the first transmembrane (TM1) region (residues G118-F138 from UniProt entry Q99942) of the RNF5 protein, (B) the second transmembrane (TM2) region of the RNF5 protein (residues S160-I180, UniProt entry Q99942), and (C) the G118-I180 region of RNF5 (UniProt entry Q99942) including both the TM1 and the TM2 regions of the protein. In all panels, magenta is used for EphA2-Sam and cyan is used for the EH region (residues I916-M918 and P952-Y960). TM1 is colored light sea green in AF2 models and orange in AF3 models (A and C), while the TM2 region is colored blue in AF2 and green in AF3 models (B and C). pLDDT, pTM, and ipTM scores of the best models are indicated along with the AF3 ranking scores [52,59,107,108].

Again, the structural prediction of the RNF5 TM1/EphA2-Sam complex was first achieved by AF2 [49,55] (Fig. 8A and Fig. S18A). In agreement with studies on isolated domains, the 5 best AF2 models appear rather different from each other as diverging in both secondary structure content and orientation of the TM1 ligand with respect to EphA2-Sam (Fig. 8A and Fig. S18A). Indeed, also within the complex, TM1 is predicted as mostly disordered while the perfect Sam domain fold is foreseen for EphA2-Sam with large accuracy and remains consistent among all 5 AF2 produced models.

Analyses of AF confidence scores give further insights. The pLDDT global values are relatively high, indicating good local accuracy, but this outcome might be heavily influenced by the high quality and accurate prediction of the EphA2-Sam domain. Indeed, the AF2 pLDDT scores of the isolated TM1 region (Fig. 5A and Fig. S7A) are lower than those obtained when TM1 is in complex with EphA2-Sam that, with its larger dimension, tends to increase the pLDDT values due to the high accuracy related to its structure prediction. The ipTM score instead is low, reflecting the difficulties encountered by AF2 in correctly positioning the TM1 peptide relative to EphA2-Sam, but again, the ipTM score might be influenced by the small TM1 peptide dimensions and its high disordered state.

The AF2 PAE scores (Fig. S15) point out, in agreement with the other AF confidence scores, that the accuracy of TM1 structure prediction is low (see on-diagonal rectangles relative to the B chain of the 5 predicted models on the third row of Fig. S15), differently from the EphA2-Sam structure whose prediction is always very accurate. The orientational variability of TM1 with respect to EphA2-Sam is evidenced instead by the high PAE values in the off-diagonal rectangles (Fig. S15) [117].

Similar results are obtained with AF3 [51,52] (Fig. 8A and Fig. S18B) that still predicts a disordered TM1 peptide with variable conformations changing from one model to the other within the 5 best predictions of its complex with EphA2-Sam. The same models do not preserve a unique orientation of the peptide with respect to the EphA2 chain (Fig. 8A and Fig. S18B). Moreover, the AF2 models of the TM1/EphA2-Sam complex appear rather diverse with respect to those generated by AF3 (Fig. 8A, Fig. S18A and B, and Table S4). The high RMSD values reported in Table S4 reflect the differences observed among best AF2 and AF3 complex predictions that correlate with the large disorder state dominating the TM1 peptide and its orientational variability with respect to EphA2-Sam in the 5 best predicted models. Although the accuracy of these predicted models is rather limited, it is interesting to note that both AF2 and AF3 tend to exploit as TM1 binding site the EphA2-Sam region that includes the EH interface (Fig. 8A and Fig. S18A and B), which also represents the binding site of other Sam domains like that from the lipid phosphatase Ship2 [27,36] or the adaptor protein Odin [35,37].

##### AF predicted model of the RNF5 TM2/EphA2-Sam complex

The TM2 domain represents the experimentally validated RNF5 interaction site with EphA2-Sam [43]; this is a membrane anchoring domain (see the “TM2 region” section) that accordingly is predicted by both AF2 and AF3 (Fig. 5B and Fig. S9A and B) to assume a stable helical conformation.

All structure features described in the “TM2 region” section regarding the isolated TM2 domain are preserved in the context of its complex with EphA2-Sam. The 5 best AF2 (Fig. 8B and Fig. S19A) and AF3 (Fig. 8B and Fig. S19B) models of EphA2-Sam in complex with the TM2 domain consist of a helical TM2 peptide mainly interacting with the EphA2-Sam interface close to its EH binding site.

The pLDDT AF2 and AF3 scores are high, thus highlighting accurate structure predictions for both EphA2-Sam and the TM2 RNF5 region; in contrast, low ipTM scores witness the inability of AF2 and AF3 (Fig. 8B and Fig. S19A and B) to unequivocally position the TM2 domain inside EphA2-Sam binding sites due likely to the small TM2 peptide dimensions that favor dynamic complexes. Consequently, a large orientational variability can be observed among both the 5 best AF2 models and the 5 best AF3 models (Fig. 8B and Fig. S19A and B), where a helical TM2 peptide interacts mostly with the EH site and adjacent regions of EphA2-Sam by assuming an ensemble of diverse orientations.

In line with the other confidence scores, AF2 PAE diagrams (Fig. S15) indicate an accurate structure prediction for the isolated EphA2-Sam and TM2 chains in all 5 AF2 models but better interchain PAE scores in the first and second models with respect to the other 3 predicted structures, thus indicating again the failure of AF to guess a unique interaction mode in between EphA2-Sam and the short TM2 peptide region (Fig. S15, fourth row).

Analyses with UCSF Chimera X [60] (version 1.5) of the 5 best AF2 predicted models of TM2 in complex with EphA2-Sam point out that the C-terminal TM2 peptide edge, encompassing approximatively residues 175 to 180 (i.e., FWLLSI), is mainly responsible for engagement of EphA2-Sam (Fig. 8B and Fig. S19A). Interestingly, residues important for binding of EphA2-Sam to other Sam domains, including Ship2-Sam, like K917, G953, R957, and K956 [36,41], appear indeed to be as well involved in providing intermolecular contacts at the EphA2-Sam/TM2 interface (Fig. 8B and Fig. S19A) as shown by the LigPlot+ [63,64] diagrams reported in Fig. S20.

Regarding the AF3 models of the EphA2-Sam/TM2 complex, inspection with UCSF Chimera X [60] demonstrates a slightly different structural interaction topology with respect to what was seen in the AF2 models, as in a few of the 5 best AF3 predicted structures, a higher number of TM2 residues seem to participate to the complex interface, consequently providing a larger and possibly more stable interaction interface (Fig. 8B and Fig. S19B). Overall, by considering all 5 AF3 models, TM2 a.a. residues that might be in contact with EphA2-Sam are positioned within the RNF5 162 to 175 a.a. segment (i.e., 162-QDSLFLFLAIFFFF-175) (Fig. 8B and Fig. S19B). In the AF3 models, the EphA2-Sam EH interface appears again involved in TM2 binding also through a few residues known to be crucial for Sam–Sam associations involving EphA2 [36,37]. However, as the 162 to 175 TM2 primary sequence is more hydrophobic than polar, we cannot envision the formation of electrostatic interactions with the positively charged side chains of residues K956, R957, and K917 that instead dominate the formation of Sam–Sam ML/EH complexes mediated by EphA2-Sam.

Because of the different orientations of TM2 inside the EphA2-Sam binding site, AF2 and AF3 predictions appear different from each other, although they share a common good fold of the isolated EphA2-Sam and TM2 chains and the similar location of the TM2 binding site on the EphA2-Sam surface surrounding the EH region (Fig. 8B, Fig. S19, and Table S4).

As also pointed out in the “Modeling the entire RNF5 protein in complex with EphA2-Sam” section when discussing the interaction between EphA2-Sam and the entire RNF5 protein, given the involvement of the EH interface, it seems that formation of a complex between EphA2-Sam and the RNF5 TM2 domain should inhibit other Sam–Sam associations mediated by EphA2. In the context of cancer cells, this might indicate that depending on the cell type and relative levels of RNF5 and different EphA2 binding partners, the receptor could switch from one interaction to the other to differently modulate pro- and antioncogenic routes through differential regulation of tyrosine and/or serine phosphorylation.

##### AF predicted model of the RNF5 TM1+TM2 region in complex with EphA2-Sam

Finally, we modeled through AF2 [49] and AF3 [51,52] predictions the interaction of an extended TM region to EphA2-Sam. Again, everything already discussed in the “Extended TM1 plus TM2 amino acid stretch” section related to the isolated TM1+TM2 peptide applies also to the peptide when in complex with EphA2-Sam as the global fold approximatively remains the same when placed in the longer amino acid segment including also the TM2 and the TM1–TM2 linker region, and TM1 tends to assume a more ordered conformation with increased helical content while the TM2 region maintains a stable helical fold. The pLDDT confidence score for both the AF2 and AF3 models of EphA2-Sam in complex with TM1+TM2 (Fig. 8C and Fig. S21) appears slightly higher with respect to the isolated TM1–TM2 region (Fig. 5C and Fig. S13), indicating a globally local good structure prediction. It is worth noting that this increase of pLDDT could be due to the presence of the larger EphA2-Sam for which a very accurate structure prediction can be achieved by AF (Fig. 8C and Fig. S21A and B). The ipTM confidence scores are low, thus indicating again that the orientation of TM1+TM2 in the EphA2-Sam binding site can be considered not reliable, although the ipTM scores need to be cautiously employed to draw conclusions as their values are more sensitive to the disorder that in this case is brought by TM1 but also by the linker region and the still low dimensions of the TM1+TM2 protein fragment. A more reliable parameter to check is the PAE, so we generated AF2 PAE diagrams also to complement ipTM-related data (Fig. S15). For the EphA2-Sam in complex with the TM2+TM1 region, the AF2 PAE scores (Fig. S15) in agreement with the other AF confidence scores highlight higher accuracy for the EphA2-Sam and TM2 structure predictions as evidenced in the on-diagonal rectangles referring to the related a.a. sequences with respect to the TM1 prediction that is instead rather poor and consequently enhances the inaccuracy of the prediction of the relative orientation of the global TM2+TM1 region with respect to EphA2-Sam (Fig. S15, fifth row). Analysis of the EphA2-Sam complexes with the TM1+TM2 peptide with the molecular graphics software UCSF Chimera X [60] indicates a scenario very similar to what was observed for the TM2 peptide/EphA2-Sam complex where the TM2 region also represents the engagement point of EphA2-Sam, in agreement with experimental data [43].

In detail, in the AF2-generated 5 best models of EphA2-Sam in complex with TM1+TM2, the TM2 C-terminal region encompassing the segment from 172 to 180 should provide crucial intermolecular interactions with an EphA2-Sam region close to the EH interface of EphA2-Sam. Regarding the AF3 models, as already pointed out in the case of the TM2 peptide alone, there is a larger variability among the 5 generated AF3 models regarding the TM2 segment contacting EphA2-Sam that overall seems to globally include residues from 162 to 175. In both AF2- and AF3-generated models, involvement of EphA2-Sam residues important for binding to other Sam domain partners (i.e., 953, 956, 957, and 917) seems possible (Fig. 8C and Fig. S21).

In the end, despite the nonideal confidence scores, AF seems to correctly predict the TM2 region as responsible for interacting with EphA2-Sam. Thus, AF2 and AF3 studies provide for the first time some structural insights to be further experimentally validated, which, however, represents pilot data that can be employed to set up anticancer drug discovery approaches relying on inhibition of the RNF5/EphA2 complex and related pro-tumorigenic effects.

#### Docking studies: The TM2/EphA2-Sam complex

**Fig. 9.**
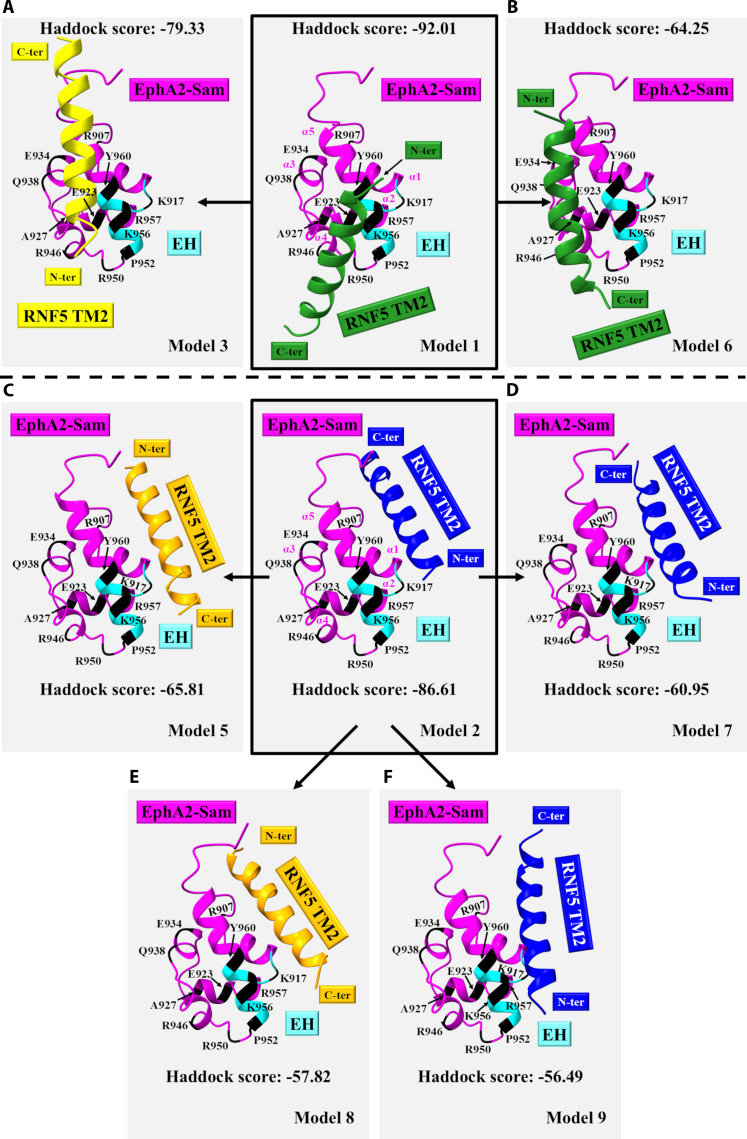
The best docking pose in terms of Haddock scores [54] for the EphA2-Sam/TM2 peptide complex is shown in the upper middle panel, while the third and sixth best models, having similar orientations with respect to the best model, are shown in panels (A) and (B), respectively. Each structure is reported in a ribbon representation: EphA2-Sam is colored magenta with a cyan EH region (residues I916-M918 and P952-Y960). The backbone of EphA2-Sam domain residues (from V904 to Q968) with solvent exposure higher than 40% and set as active during the docking calculations (i.e., R907, K917, E923, A927, E934, Q938, R946, R950, P952, K956, R957, and Y960) is highlighted in black on the EphA2-Sam surface. The TM2 peptide is shown in 2 different colors (i.e., green and yellow) to highlight the different orientation assumed by peptide with respect to the EH region of EphA2-Sam. The second-best docking pose in terms of Haddock scores [54] for the EphA2-Sam/TM2 peptide complex is shown in the lower middle panel, while the fifth-, seventh-, eighth, and ninth-best models, having similar orientations with respect to the second-best model, are shown in panels (C), (D), (E) and (F), respectively. The TM2 peptide is shown in 2 different colors (i.e., blue and orange) to highlight the different orientation assumed by peptide with respect to the EH region of EphA2-Sam.

As AF analyses, although providing some interesting insights, failed to generate a unique more reliable model of the EphA2-Sam/TM2 complex, especially regarding the mutual orientation of the 2 entities with respect to each other, we decided to conduct docking studies and verify if docking solutions resembling AF2 [49] and AF3 [51,52] best predicted models could be obtained (Fig. 9, Figs. S22 and S23, and Table S6). Docking runs were carried out through the Haddock webserver [54] using as input the NMR structure of EphA2-Sam and the best predicted AF2 model of the TM2 domain. A sort of blind docking was conducted, letting the peptide explore the whole EphA2-Sam surface to find the preferred binding site. This kind of docking is very appropriate for this study where we would like to also understand if the EH site is the best interaction interface for TM2 as it is pointed out by our AF studies. The 10 best Haddock solutions (i.e., the ones with the lowest Haddock scores) were analyzed (Fig. S22) and subdivided into 2 main subgroups: one in which the helical TM2 peptide is positioned in between the α4–α5 interface and the other featuring instead the peptide located between the α5 C-terminal helix and the α1α2 loop (Fig. 9 and Fig. S22). Model 4 shows instead the peptide positioned on a completely different side facing the α2α3 and α4α5 loops (Fig. S22D); in Model 10, the peptide is partially contacting the C-terminal tail of EphA2-Sam (i.e., the PBM region) and the most C-terminal helical turns of α5 (Fig. S22J). Model 10 seems to be a wrong docking pose as it shows a largely solvent-exposed peptide having just a few contact points with the protein, so we excluded it from further analysis. Regarding Model 4, we did not consider it further as, among the 10 best solutions, it seems to be less represented. Thus, the remaining solutions were subdivided into 2 groups: one associated with the protein/peptide structural topology of interaction represented by Model 1 (i.e., Haddock poses 1, 3, and 6 in Fig. 9A and B) and the other represented by Model 2 (i.e., Haddock poses 2, 5, 7, 8, and 9 in Fig. 9C to F).

Interestingly, among solutions belonging to the first group (Fig. 9A and B), Model 6 is even more close to Model 1 as the TM2 domain is presented facing the EphA2 binding site, made up by the α5, α4, and α3α4 loop interface, with the same “from N- to C-terminal” orientation (Fig. 9B); Model 3 shows instead a TM2 peptide with the opposite “from C- to N-ter orientation” (Fig. 9A). After noticing that, to get an idea of the most interesting intermolecular interactions that could characterize the complex between EphA2-Sam and the TM2 domain of RNF5, we generated LigPlot+ [63,64] diagrams of intermolecular contacts (Fig. S23) just for Models 1 and 3. Similarly, in the second group (Fig. 9C to F), Models 7 (Fig. 9D) and 9 (Fig. 9F) have the peptide in the same “from N- to C-ter” orientation of Model 2, differently from Models 5 (Fig. 9C) and 8 (Fig. 9E).

Thus, regarding the second group featuring solutions similar to Model 2, LigPlot+ [63,64] diagrams were generated for Models 2 (Fig. S23C) and 5 (Fig. S23D).

Analysis of LigPlot+ results and further inspection of the list of EphA2 residues providing intermolecular contacts, generated by Haddock (Table S6), pointed out that a few a.a. belonging to the EH interface (i.e., K917, G953, K956, and R957), which is responsible in EphA2-Sam for interactions with other Sam domains, are predicted also by these docking studies as potential providers of intermolecular interactions (mainly non-bonded contacts ) with the RNF5 TM2 domain (Fig. S23 and Table S6).

Finally, comparison of docking solutions with AF2 and AF3 structures predicted for the EphA2-Sam/TM2 complex revealed that AF2-generated models were rather diverse from the docking solutions while AF3-derived best models number 3 and 4 (Fig. S9B) presented some resemblance with docking solutions belonging to the second group (Fig. S22 and Fig. 9C to F), thus letting us speculate that these docking poses could at least partially include some interesting structural features crucial for the complex formation.

### Experimental investigation

Owing to the relevance of the TM2 region (Fig. 1) in RNF5 for the interaction with EphA2-Sam, conformational analyses by both CD and NMR spectroscopies were conducted with model peptides to get insights into structural features characterizing such protein segment and also experimentally validate results from AF predictions. Attempts to obtain by canonical solid-phase peptide synthesis routes protein fragments encompassing the whole TM2 segment (i.e., a.a. 160 to 180 from UniProt entry Q99942 for human RNF5; Fig. 1) were unsuccessful due to the hydrophobicity and consequent poor solubility of this aggregation-prone amino acid sequence that includes several aromatic residues along with leucine and isoleucine residues. This hydrophobicity is even more relevant at the very C-terminal part (residues from 170 to 180) that even includes a tetra-phenylalanine motif. The “FFFF” motif is known to self-assemble forming, depending on the employed experimental conditions, or particular chemical entities they are coupled to, a variety of supramolecular aggregates including nanotubes, fibrils, and even cross-beta amyloid-like structures [119–121]. In contrast, structure investigation could be carried out with the RNF5-PEP3 peptide including RNF5 residues from 151 to 170 (Fig. 1) and thus encompassing the first 10 N-terminal amino acids of the RNF5 TM2 domain. Conformational analyses were conducted by CD and NMR spectroscopies in an aqueous environment and in the presence of TFE. TFE appears a really interesting co-solvent to be employed in this kind of analysis as, being provided by a certain hydrophobicity, it may give rise in water to ordered aggregates such as micelle-like assemblies that can resemble the interior of biological membrane and consequently be very useful to study the bioactive conformation the RNF5 TM2 region might assume upon insertion in the lipid membrane [122,123].

As another putative transmembrane region (i.e., TM1) is predicted to be present in RNF5, although initially not contemplated, as mentioned above, in RNF5 protein domain organization, the model peptide RNF5-PEP2 was also generated to gain further structural insights into the conformational preferences of this fragment and to check if TM1 could somehow support TM2 in binding to EphA2-Sam. The peptide RNF5-PEP2 was designed to cover the entire TM1 domain and was a bit elongated at the N- and C-terminal sides to possibly enhance a folding process and favor observation of secondary structure elements. However, it was not possible to produce this peptide in a highly pure form due to the hydrophobicity of its sequence that appears abundant. Another issue that was encountered while attempting to employ RNF5-PEP2 in experimental studies was related to its reduced solubility especially at the concentration needed to conduct detailed NMR studies.

#### CD investigation

**Fig. 10.**
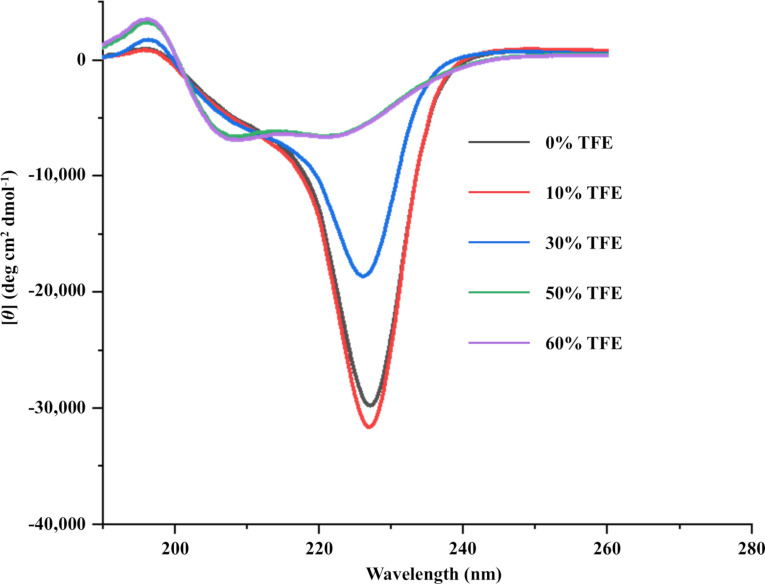
Far-UV circular dichroism spectrum of RNF5-PEP3 recorded at 100 μM concentration in 10 mM phosphate buffer (pH 7.4) and with increasing TFE amounts.

The far-UV CD spectrum of RNF5-PEP3 in phosphate buffer displayed a well-defined negative band centered at 227.6 nm, accompanied by a weaker negative shoulder near 205 to 207 nm (Fig. 10). The absence of the characteristic α-helical minima at 208 and 222 nm, together with the lack of a β-sheet-type maximum near 195 nm, indicates that RNF5-PEP3 does not adopt canonical secondary-structure elements under these conditions. The overall profile is therefore consistent with a predominantly nonregular conformation or mixed states, in agreement with its sequence composition enriched in flexible and hydrophobic residues. To further investigate the conformational contributions, the spectrum was analyzed using the BeStSel deconvolution algorithm [66]. The resulting secondary-structure distribution indicated not only measurable contributions from helix (12.7%) and antiparallel β-structure (37.0%), but also a substantial proportion of turn (26.3%) and others (24.1%) (Table S7). These results support the qualitative interpretation of the CD spectrum, indicating that RNF5-PEP3 predominantly samples flexible and mixed conformations rather than stable α-helical or β-sheet structures. In contrast, RNF5-PEP2 yielded a very weak and noisy CD signal, with only broad and low-intensity features across the 190 to 280 nm range (Fig. S24). This limited signal intensity is attributable to pronounced solubility issues, which prevented achieving a sufficiently high and homogeneous peptide concentration for an interpretable CD profile. As a result, no reliable structural information could be extracted from this spectrum, and RNF5-PEP2 could not be analyzed further under the tested experimental conditions.

The far-UV CD spectra of RNF5-PEP3 recorded in the presence of increasing amounts of the membrane mimetic cosolvent TFE are also shown in Fig. 10.

Upon the addition of TFE, progressive changes in the CD shape and intensity were observed. At low TFE content (10% to 30% v/v), the negative band becomes more pronounced and slightly shifts, indicating a solvent-induced stabilization of more ordered conformational states. At higher TFE concentrations (50% to 60% v/v), the spectra show a clearer definition of the negative band near ~222 nm. In this solvent regime, the appearance of the 2 characteristic minima at approximately 208 and 222 nm becomes evident, indicating a relevant stabilization of α-helical secondary structure. In agreement with results from the BeStSel deconvolution algorithm [66] (Fig. S25 and Table S7), overall, the titration reveals a gradual and concentration-dependent conformational response of RNF5-PEP3 to TFE, highlighting the sensitivity of the peptide to its solvent environment and its propensity to populate more structured states under a membrane mimetic milieu like that induced by TFE [123].

#### NMR characterization

NMR techniques were employed to investigate the conformational properties of the RNF5-PEP3 peptide.

This peptide is soluble in 100% DMSO, giving rise to a 1D [^1^H] spectrum (Fig. S26A) characterized by sharp signals typical of a disaggregated entity. Instead, the 1D [^1^H] NMR spectrum of an RNF5-PEP3 sample in PBS containing just 0.6% DMSO (330 μM concentration, pH 7.25), was characterized by large and poorly intense peaks (Fig. S6B), likely indicating the occurrence of aggregation phenomena. In presence of 0.6% DMSO, the RNF5-PEP3 peptide presents indeed reduced solubility as also demonstrated by the cloudiness of the NMR sample. A serial dilution of this RNF5-PEP3 sample was achieved, and 1D [^1^H] spectra at a peptide concentration of 100 and 50 μM were registered (Fig. S27); the spectra appeared again characterized by large peaks, indicating that the peptide remains aggregated even at the lower employed concentrations. Then, based on CD data, which suggested an increase in peptide helical structuration following the addition of increasing TFE amounts (Fig. 10), we registered NMR spectra in the presence of 60% TFE [123,124]. The 1D [^1^H] spectrum (Fig. S28A) showed for the peptide under this solvent condition (PBS/TFE 40/60 v/v) a good solubility and low aggregation propensity (i.e., rather sharp NMR signals). Analyses of 2D [^1^H, ^1^H] NMR spectra of RNF5-PEP3 in TFE (Fig. S28B and C) allowed us to almost completely achieve unambiguous proton resonance assignments (Table S8). In the 2D [^1^H,^1^H] NOESY spectrum, the presence of sequential H_N_i-H_N_i+1 contacts (Fig. S28C, right panel) likely indicated some peptide structuration. We also registered a natural abundance 2D [^1^H,^15^N] HSQC spectrum (Fig. S29A). Although the signal dispersion is limited in this spectrum, as occurring for flexible and largely disordered species, we can clearly detect all backbone and side-chain H_N_ (Fig. S29A), suggesting, in agreement with results from CD, that they were not completely solvent exposed possibly because residual secondary-structure elements are present in solution under the more hydrophobic membrane mimetic environment related to TFE. The analysis of peptide Hα chemical shift deviations with respect to predicted random coil values [75,125] (Fig. S29B) evidenced the occurrence of negative values, pointing out helical structuration in the C-terminal portion of the peptide going from W161 to A170. Interestingly, this peptide fragment mainly encompasses the region of the RNF5 TM2 domain spanned by RNF5-PEP3 (Fig. 1), thus suggesting a larger helical propensity of this part of the sequence. Intriguingly, these outcomes also appear in some agreement with the AF2 and AF3 structure predictions for RNF5 TM2 revealing helical conformations (Fig. 5B and Fig. S9). However, the percentage of helical content that can be estimated for RNF5-PEP3 in 60% TFE, based on Hα deviation with respect to random coil values (Fig. S29B), is equal to 36% [76], highlighting, in agreement with CD spectra deconvolution data (Fig. S25), that the peptide overall conformation can be better described by an equilibrium between a partially helical and a more disordered state. Because of the presence in the RNF5-PEP3 sequence of a proline (P157), we investigated on possible occurrence in NMR spectra of both cis and trans conformations that might increase peptide conformational variability, by also registering a 2D [^1^H,^13^C] HSQC spectrum (Fig. S30A). In this experiment, only one set of ^13^CHδ peaks related to P157 was detectable (Fig. S30B), and the chemical shift difference between ^13^CHβ and ^13^CHγ peaks (4.7 ppm) was indicative of a proline in a trans configuration (Fig. S30C) [126]. The prevalent occurrence of a proline in the trans configuration can be further proved by inspection of the NOESY spectrum where the Hδδ′ P157-Hα H156 NOE pattern characteristic of the trans form can be seen (Fig. S30D) [74,126].

#### NMR interaction studies: EphA2-Sam versus RNF5-PEP3

Immunoprecipitation data employing an RNF5 protein construct lacking almost completely the C-terminal transmembrane domain (i.e., residues 165 to 180) demonstrated that RNF5 TM2 could be responsible for binding to EphA2-Sam [43,44].

**Fig. 11.**
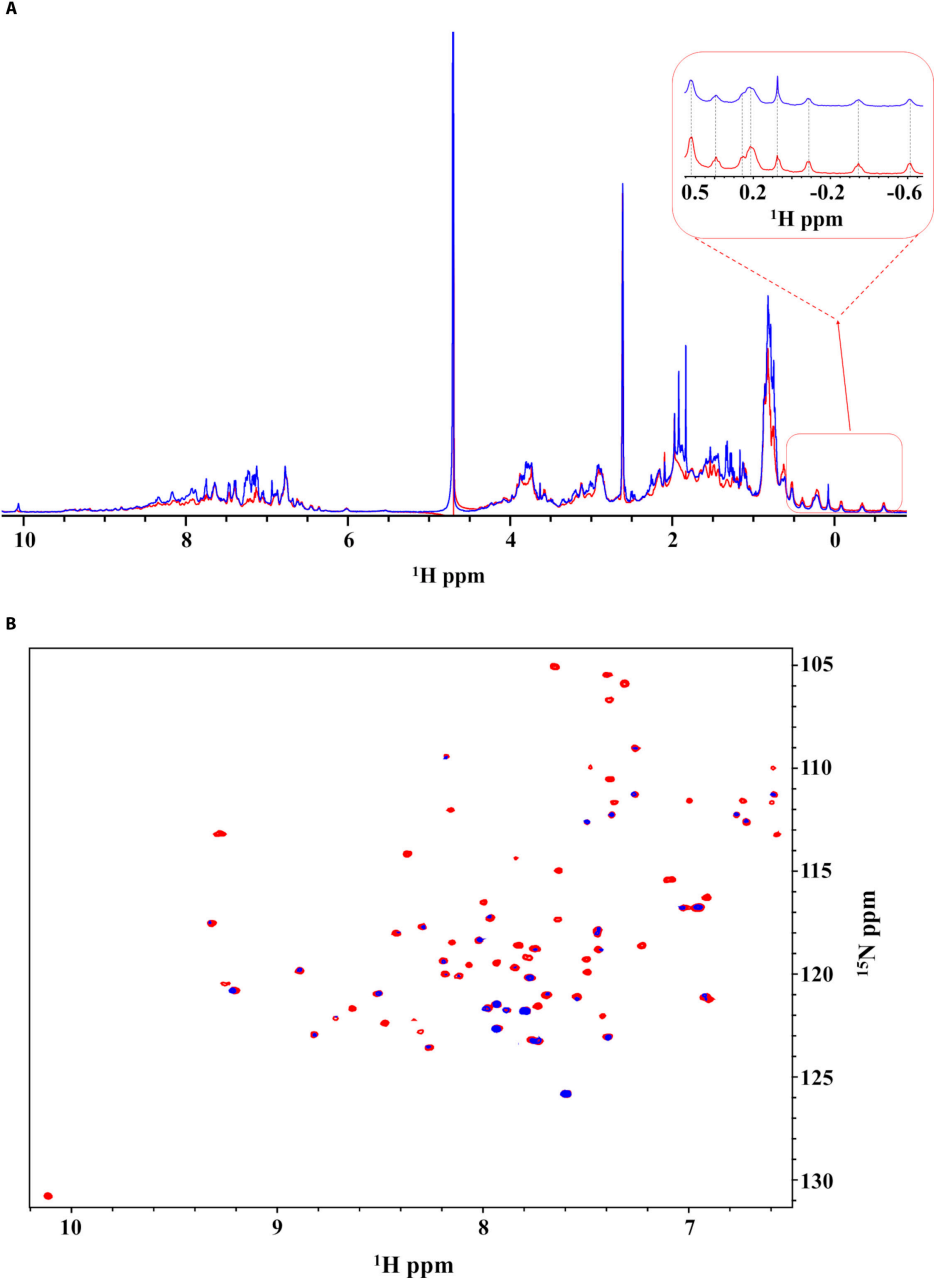
Superposition of 1D [^1^H] (A) and 2D [^1^H-^15^N] HSQC spectra (B) acquired for EphA2-Sam alone (14.8 μM—red) and after addition of RNF5-PEP3 (330 μM concentration—blue). Each NMR sample for interaction assays contains the same amount of DMSO (i.e., 3.6 μl). In (A), the red inset shows an expansion of the stacked spectra including only the protein methyl peak region.

Thus, experimental interaction studies were conducted to investigate the possible binding of the RNF5-PEP3 peptide to EphA2-Sam by NMR techniques. 1D [^1^H] and 2D [^1^H,^15^N] HSQC spectra were registered with EphA2-Sam alone (14.8 μM) and after the addition of RNF5-PEP3 (330 μM). The comparison of 1D spectra of the protein methyl region in the absence and in the presence of the peptide indicates that it does not induce chemical shift variations of the protein peaks but only a decrease in peak intensity (Fig. 11A). The HSQC spectrum of the ^15^N-labeled EphA2-Sam in the presence of RNF5-PEP3 also evidenced a decrease of almost all protein peak intensities (Fig. 11B). The large loss of protein signals is related to peak enlargement and may be due to peptide aggregation propensity and the formation of large protein/peptide aggregates, inducing unspecific interactions involving the whole protein surface [127]. To further assess this important point and exclude a specific sample-dependent effect, NMR 1D and 2D chemical shift perturbation experiments were repeated at multiple increasing peptide concentrations (i.e., 45, 160, and 250 μM) with freshly prepared NMR samples (Fig. S31A to C). At 45 μM concentration and a peptide/protein molar ratio equal to 3:1 (Fig. S31A), no changes in the NMR spectra could be detected, thus pointing out that aggregation phenomena are less relevant; when the protein is instead in the presence of peptide at 160 μM concentration (Fig. S31B), a decrease in peak intensity starts to be evident in the 2D [^1^H, ^15^N] HSQC spectrum of EphA2-Sam and becomes more relevant by additionally increasing peptide amount to 250 μM concentration (Fig. S31C). Mapping changes into the 3D structure of EphA2-Sam (Fig. S31D) clearly shows an unspecific effect involving residues positioned around the whole protein surface rather than being confined at a specific binding site (Fig. S31D). The extensive disappearance of protein peaks in the HSQC spectrum following the addition of the peptide at the highest concentrations likely indicates the formation of large aggregates including multiple peptide and protein copies.

Given the observed unspecific binding, the interaction between RNF5-PEP3 and Ship2-Sam was also tested again through chemical shift perturbation studies with [^1^H, ^15^N] HSQC spectra that were recorded with ^15^N-labeled Ship2-Sam in the presence and absence of the peptide (Fig. S32). Such interaction studies indicated that RNF5-PEP3 is unable to bind Ship2-Sam as only very minor changes could be observed in the spectrum of the protein upon the addition of the peptide (Fig. S32). It seems that this RNF5-PEP3 peptide sequence exhibits somehow a better capacity to interact with EphA2-Sam (Fig. 11 and Fig. S31) with respect to Ship2-Sam (Fig. S32). Moreover, a displacement-like experiment (Fig. S33) in which unlabeled Ship2-Sam was added to the complex formed by ^15^N-labeled EphA2-Sam and RNF5-PEP3, in a ~3- and ~7-fold sub-stoichiometric excess with respect to EphA2-Sam, failed to show recovery of the spectrum corresponding to the Ship2-Sam/EphA2-Sam complex (Fig. S33A to C). This outcome is likely related to the formation of large EphA2-Sam/RNF5-PEP3 supramolecular aggregates that hamper the access of Ship2-Sam to the EH interface, corresponding to its binding site on the EphA2-Sam surface.

Interestingly, the TM2 region includes within its primary sequence a tetra-phenylalanine motif (residues 172 to 175, Fig. 1), which is external to the RNF5-PEP3 sequence representing, as previously mentioned, an aggregation-prone amino acid pattern [119–121]. Pilot binding experiments with a tetra-phenylalanine-containing peptide available in our laboratory demonstrate that the “FFFF” sequence alone is not responsible for high-affinity binding to EphA2-Sam (data not shown).

In conclusion, these results allow us to speculate that possibly the whole RNF5 TM2 domain, including residues from 160 to 180, is needed to provide specific binding to EphA2-Sam. This larger peptide region, differently from the small fragment spanning the RNF5-PEP3 peptide, could assume a more ordered and well-organized helical structural arrangement that, by hampering aggregation partially connected to disorder, could be at the basis of an efficient molecular recognition mechanism. Of course, experimental validation, which will be achieved in a follow-up manuscript, is needed to confirm such hypotheses.

#### Interaction studies by MST: EphA2-Sam and Ship2-Sam versus RNF5-PEP3

To further support NMR data, pilot MST interaction assays were set up to validate RNF5-PEP3 binding to EphA2-Sam; MST experiments were also conducted to check unspecific binding of RNF5-PEP3 to Ship2-Sam as well. MST interaction analyses of Sam domains to peptides have been very well established in our laboratories [127,128].

In agreement with NMR data, MST revealed a clear but weak interaction of EphA2-Sam with RNF5-PEP3 with a dissociation constant (*K*_D_) in the hundred micromolar range. MST revealed a certain binding of the peptide to Ship2-Sam as well (Fig. S34). In agreement with NMR data, MST assays pointed out some preference of the peptide to interact with EphA2 rather than Ship2 as can be speculated by inspecting and comparing the dose–response interaction curves (Fig. S34). Precise quantitative estimates of the binding between EphA2-Sam and Ship2-Sam and the RNF5-PEP3 peptide were not possible because, under the experimental conditions used to run our assays, titration saturation conditions could not be reached due to peptide poor solubility at the high concentrations needed to fully complete the titration graphs (Fig. S34).

### Analysis of aggregation propensity of RNF5 and its TM2 domain

Results of NMR structural and interaction studies with the RNF5-PEP3 peptide pointed out the possible tendency of this RNF5 segment to aggregate. Interestingly, as previously mentioned, RNF5-PEP3 includes the protein amino acid segment 151 to 170 that overlaps partially (i.e., residues from 160 to 170) with the TM2 domain (i.e., a.a. 160 to 180). Given the poor solubility of the whole TM2 region and the inclusion of too many hydrophobic amino acids, it was not possible to synthesize by standard solid phase routes a peptide mimicking the whole TM2 domain. Thus, to better understand structural features at the basis of molecular recognition between EphA2-Sam and the RNF5 TM2 region, bioinformatic tools and the AF2 multimer module [95] were employed. Indeed, the aggregation propensity of the whole protein and the isolated 151 to 170 fragment, corresponding to the RNF5-PEP3 peptide, were analyzed following structure predictions of homodimeric RNF5 forms.

The AF2 model of a putative RNF5 homodimer is shown in Fig. S35 and appears characterized by a peculiar structural arrangement. The 5 best AF2 models, although not identical in between each other, possess very similar interaction interfaces stabilized by intermolecular contacts (see LigPlot+ [63,64] diagrams in Fig. S36 and Table S9) provided by the most ordered RING and TM2 domains.

Indeed, in each model (Fig. S35), 2 RING domains and 2 TM2 helices, one from each chain, at the N-terminal and C-terminal edges, respectively, contact each other, forming 2 sorts of lateral clips that generate a central channel (Fig. S35). The interaction interfaces are mainly stabilized not only by non-bonded contacts involving hydrophobic residues but also by several H-bonds (Fig. S36 and Table S9). Although residues from the RING (a.a. 27 to 68) and TM2 (a.a. 160 to 180) domains provide in each model the largest number of interactions (Fig. S36 and Table S9), the specific intermolecular interaction network is different in each model as the accuracy of the AF2 prediction is rather low and consequently the structure convergence is poor (Fig. S36). A few residues positioned in the linker region between the RING and TM1 (a.a. 69 to 117) are also participating in dimer interfaces (Fig. S36). Much care needs to be put when trying to determine a detailed structure interaction topology that should drive RNF5 aggregation based on these AF2 models as the AF2 confidence scores point to a low-precision structure prediction. The 5 best models of a putative RNF5 homodimer represent in fact predictions characterized by a rather low local and global accuracy as pointed out by pLDDT values below 60 and pTM values mainly close or below 0.5, whereas ipTM values (below 0.5) further highlight uncertainties in the reciprocal orientations of the 2 chains (Fig. S35) [129]. Next, the bioinformatic tools A3D 2.0 [78] (Fig. S37 and Table S10) and AggreProt [79] (Fig. S38 and Table S11) were employed as well to estimate the propensity of each RNF5 residue to aggregate. Regarding A3D 2.0 [78], this represents a structure-based instrument, and the 3D coordinates of the 5 best AF2 models of the isolated RNF5 protein or the RNF5-PEP3 peptide were used as input to run the aggregation prediction. On the other side, AggreProt [79] relies instead more on a sequence-based prediction but that can also be fed by PDB coordinates as input. AggreProt interestingly also provides information on the SASA (solvent accessible surface area) and protein regions possibly able to form transmembrane domains. It can be expected that low SASA might correlate with buried residues, such as those inside a protein core, which are unlikely able to contribute to aggregation phenomena. Thus, when assessing aggregation with AggreProt, aggregation hotspots are given mainly by those residues possessing both “Agg” and “SASA” scores over the thresholds (i.e., 0.25 [79] and 0.25, respectively). Thus, according to the AggreProt webserver for the RNF5 protein, the residues with the largest tendency to aggregate (i.e., those with both high ”Agg” and “SASA”) fell mainly within the RING domain and the C-terminal regions encompassing the TM1 and TM2 domains, which are also predicted correctly to form transmembrane domains [130] (Fig. S38 and Table S11). Similar results were obtained with the A3D 2.0 [78] server that predicted the TM1 and TM2 regions as pretty well aggregation prone followed by a few residues within the RING domain (Fig. S37 and Table S10).

Similar studies were conducted for the RNF5-PEP3 peptide. A model of a homodimeric peptide was built by the AF2 multimer module. The 5 best AF2 models (Fig. S39) show for the 2 subunits helical conformations in the C-terminal regions and contact in between monomeric units, forming the dimer interface, positioned inside these helical regions at the C-terminal side. In 4/5 best predicted AF2 dimeric structures (Fig. S39A, B, C, and E), the helical region runs parallel with respect to each other. In only one of the best predicted models (Fig. S39) is the contact surface in the dimer given by just a few C-terminal residues within the helical domain of the peptide. Analyses of intermolecular interactions by LigPlot+ (Fig. S40A to E and Table S12) well show that, among the amino acid residues belonging to the RNF5-PEP3 peptide, only those encompassing the region corresponding to TM2 contribute to aggregation by mainly providing non-bonded interactions (i.e., residues 161 to 170). In addition, outside the TM2 region, only residues H156 and A158 are part of the contact surface in between 2 monomeric chains in 2/5 and 1/5 best models, respectively. The predictions of aggregation-prone residues within the peptide sequence with A3D 2.0 [78] (Fig. S41 and Table S13) and with AggreProt [79] (Fig. S42 and Table S14) are in perfect agreement with the AF2 results indicating that the C-terminal stretch S164-A170 represents likely an aggregation hotspot.

## Concluding remarks

Herein, we reported on a computational approach to gain fast structural features of novel and still poorly investigated PPIs for which very little is known at the molecular recognition level. As a model system, we employed the binding of the E3 ubiquitin ligase RNF5 to the EphA2 receptor, an interaction that is important to modulate receptor stability. Overexpression of RNF5 in certain breast cancer cells induces ubiquitination and degradation of EphA2, and this down-regulation of receptor produces pro-cancer effects. It can be speculated that hampering formation of the RNF5/EphA2 complex might have a positive effect on breast cancer cells, and based on this, one could envision setting up drug discovery approaches, including structure-based design of molecular tools (like peptides or small molecules) blocking the target interaction. To the best of our knowledge, the structural features of such complex have not been elucidated yet; to fill in this gap, *in silico* strategies relying on cutting-edge AI tools like AlphAFold (AF) can be supportive. Thus, we set up a protocol relying on structural predictions by AF2 and AF3, bioinformatics tools to investigate disorder grade and aggregation propensities of proteins and docking studies. In the end, we even conducted pilot experimental validation.

*In silico* approaches represent often a convenient way to quickly gain a substantial amount of data to support drug discovery or protein design projects and suggest ways to prioritize experimental validation, thus reducing the time required to accomplish related tasks and speeding up the whole research pipeline. For instance, a variety of computational workflows have been recently employed to establish functional and structural effects of disease-relevant proteins mutations [131–133] or to establish mutations able to enhance the pharmaceutical potential of therapeutic proteins [134]; to discover novel therapeutic protein targets or PPIs [135]; to predict original ligands with therapeutic potential; or to design optimized therapeutic protocols based on specific drug combinations [136].

In this context, we believe that our approach allowed us to collect important data and 2 plausible interaction models for the binding of EphA2-Sam with the TM2 domain of RNF5 that can be exploited to set up structure-based drug discovery approaches to find novel anticancer compounds targeting the EphA2/RNF5 axis.

While co-immunoprecipitation experiments indicated that only the TM2 region is responsible for the interaction with EphA2-Sam [43], interestingly, our AF predictions of RNF5/EphA2 hetero complexes allow us to speculate that the RNF5 RING domain could indeed be involved into direct interactions with EphA2-Sam. This is a novel finding that should be soon better assessed by experimental studies as it could really open new opportunities in the anticancer drug discovery field.

Another interesting insight, gained by this study, concerns the EH interface of EphA2-Sam that appears to be a “universal” receptor binding site. Being residues of the EH site (like G953, R957, and K917 from EphA2-Sam) important for interaction with Ship2-Sam and other modulators of receptor stability, like Odin, implicated also in binding to the TM2 domain of RNF5 (Fig. S20), it is possible that, depending on the type of cancer cells and the overexpression of one binding partner rather than another, EphA2-Sam switches the interaction network to differentially modulate the pro- and antioncogenic signaling pathway and consequently fine-tuning tyrosine/serine phosphorylation levels to achieve the proper outcomes.

Our results should of course be considered as pioneering work and much more experimental studies should be conducted to better validate our findings.

## Data Availability

Additional data will be made available on reasonable request.
