# Supplementary material for: The Interaction between the Tyrosine Kinase Receptor EphA2 and RNF5: Structural Insights from an In Silico Approach
Source: Comput Struct Biotechnol J. 2026 Jul 17;35(1):0166. doi: 10.34133/csbj.0166 (PMC13376385; doi:10.34133/csbj.0166)
Supplement: Supplementary 1 — Figs. S1 to S42 Tables S1 to S14 [file csbj.0166.f1.docx]

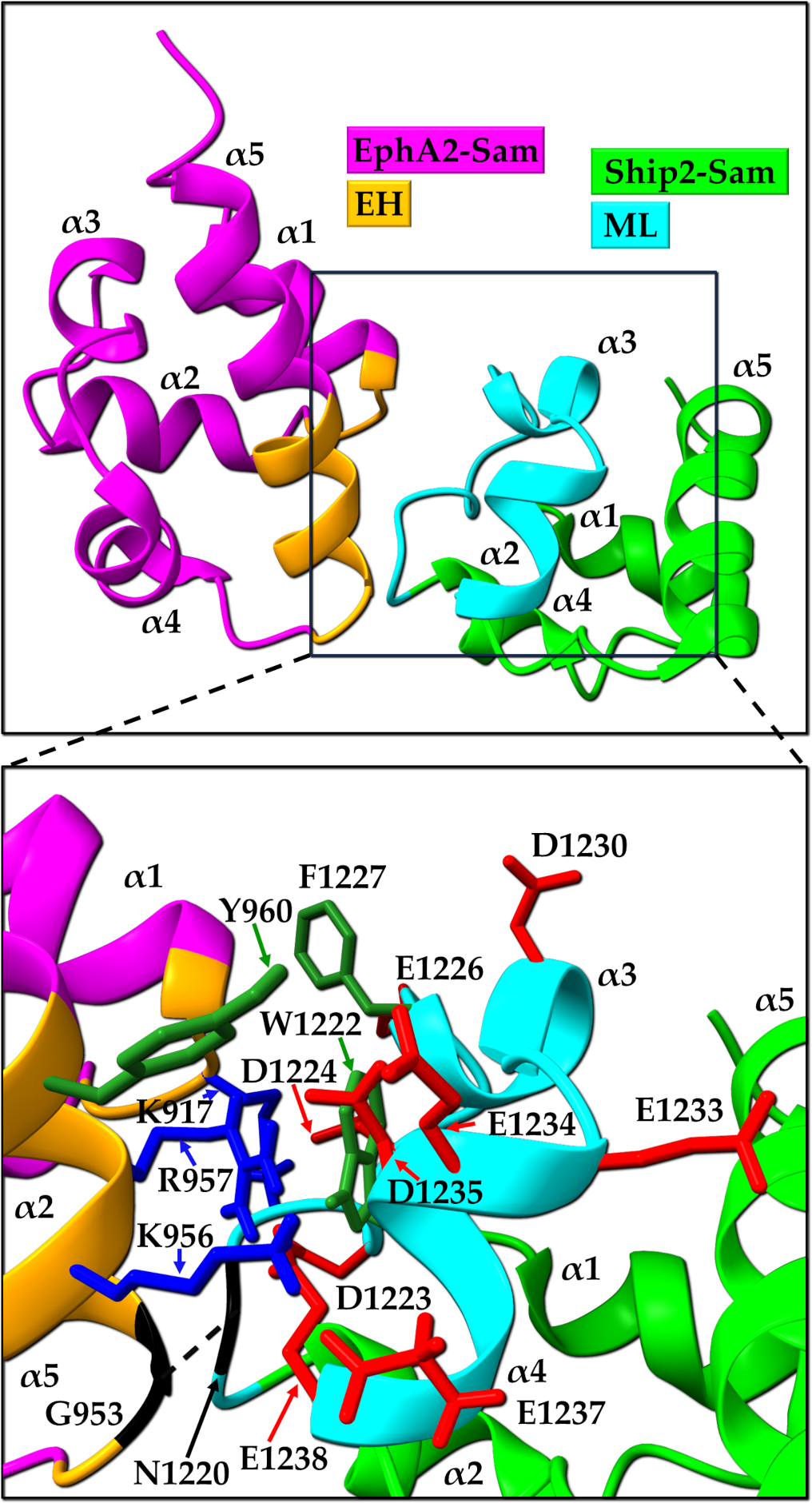
**Supplementary Information**

**Fig. S1.** Structure of the EphA2-Sam (magenta)/Ship2-Sam (light green) complex (PDB code 2KSO [41], first NMR conformer). The EH and ML interfaces in EphA2-Sam (residue range I916-M918 and P952-Y960 from UniProt [57] entry P29317) and Ship2-Sam (residues H1219-E1238 from UniProt entry O15357) are colored in orange and cyan, respectively. The lower panel reports a zoomed view of the EH/ML interacting interfaces, where green, blue and red are used for aromatic, positively charged, and negatively charged residues, respectively. The backbone of the residues involved in the characteristic Sam domain H-bond (H_N_ G953 from EphA2-Sam and cO N1220 from Ship2-Sam) is colored black on the ribbon representation.

**Fig. S2.** HPLC profile of RNF5-PEP3 (purity 94.4%) provided by DBA Italia, s.r.l. (Milan Italy).

**Fig. S3.** Mass spectrum of RNF5-PEP3 provided by DBA Italia, s.r.l. (Milan Italy).


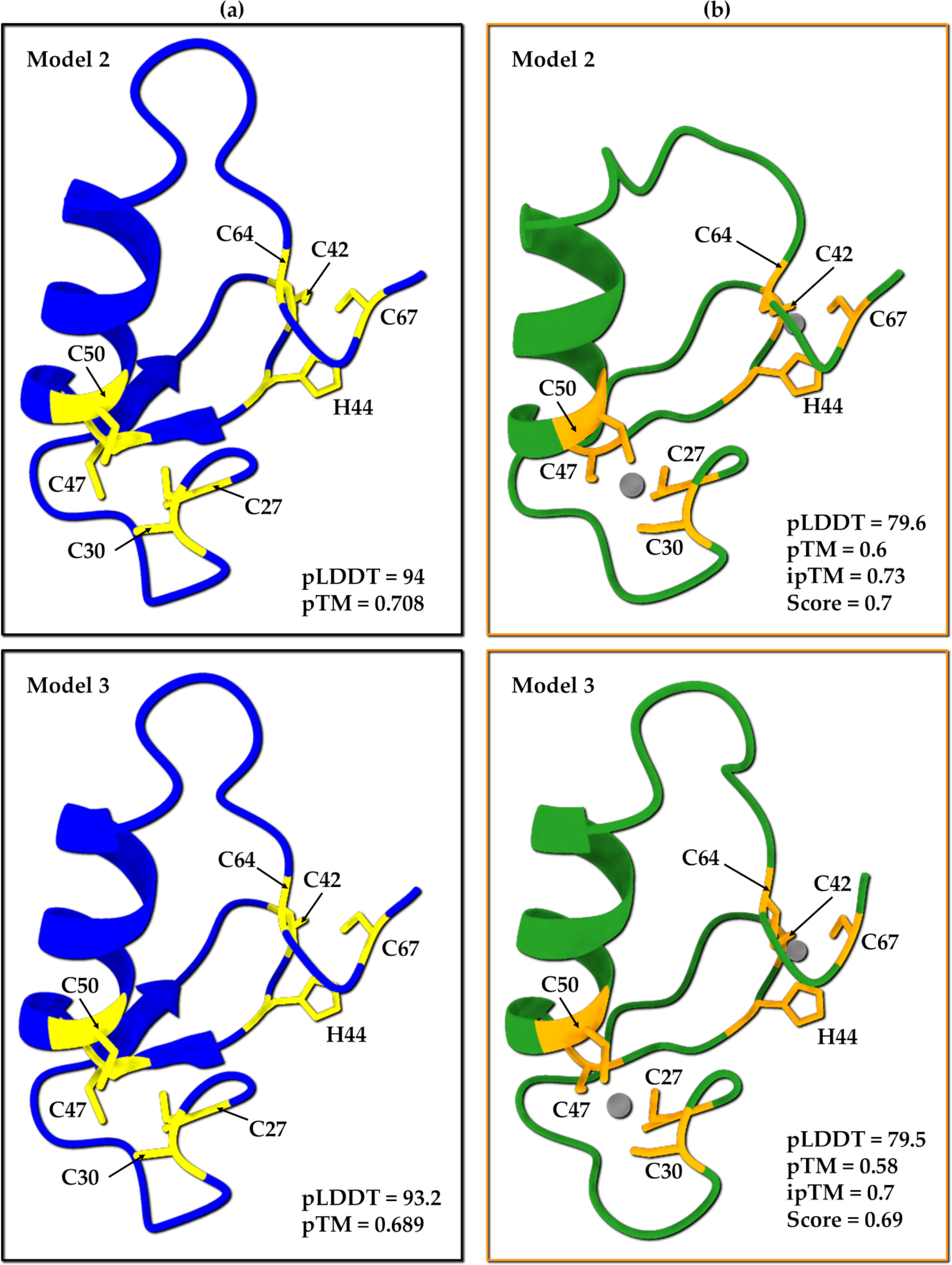


**
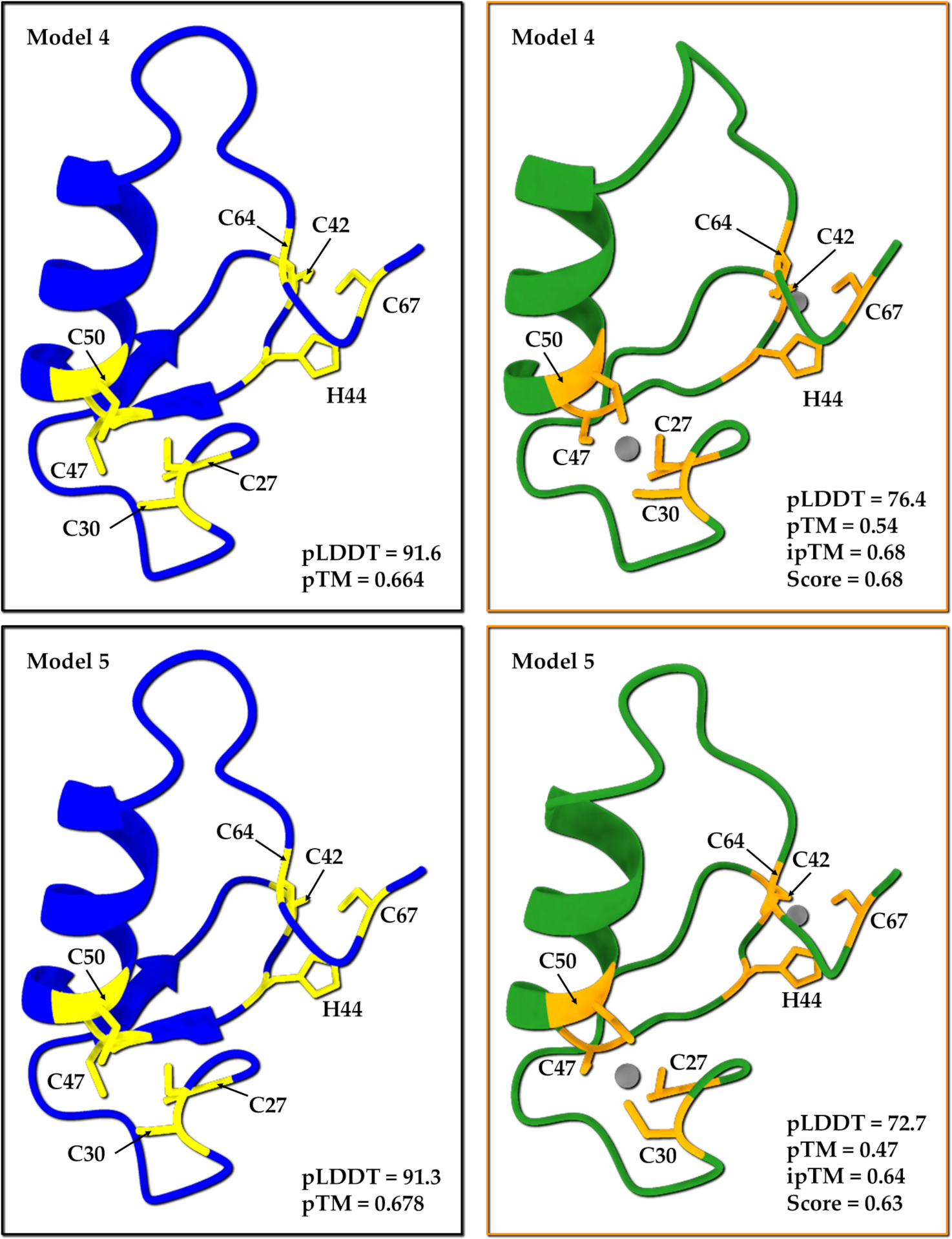
**

**Fig. S4.** (a) AlphaFold2 (AF2) [49,55] models (from 2^nd^ to 5^th^ ranked structures) of the RNF5 RING domain (residue range C27-K68 from UniProt [57] entry Q99942). The RING domain structure is shown in a ribbon representation colored blue except the residues contributing to the two Zn^2+^ ions coordination clusters HC3 (i.e., H44, C42, C64 and C67) and C4 (i.e., C27, C30, C47 and C50) that are shown in yellow with side chains reported in a neon representation (heavy atoms only). (b) AlphaFold3 (AF3) [51,52] models (from 2^nd^ to 5^th^ ranked structures) of the RNF5 RING domain (residue range C27-K68 from UniProt entry Q99942) including the two Zn^2+^ ions (shown in grey). The RING domain is colored green. Cysteine and Histidine residues participating in the two Zn^2+^ coordination clusters are colored gold with the side chains represented in a neon drawing with heavy atoms. AF pLDDT, pTM and ipTM confidence scores [107,137-139] of the 4 models are indicated in (a) and (b) panels. The AF3 ranking score [52] is also reported in (b).


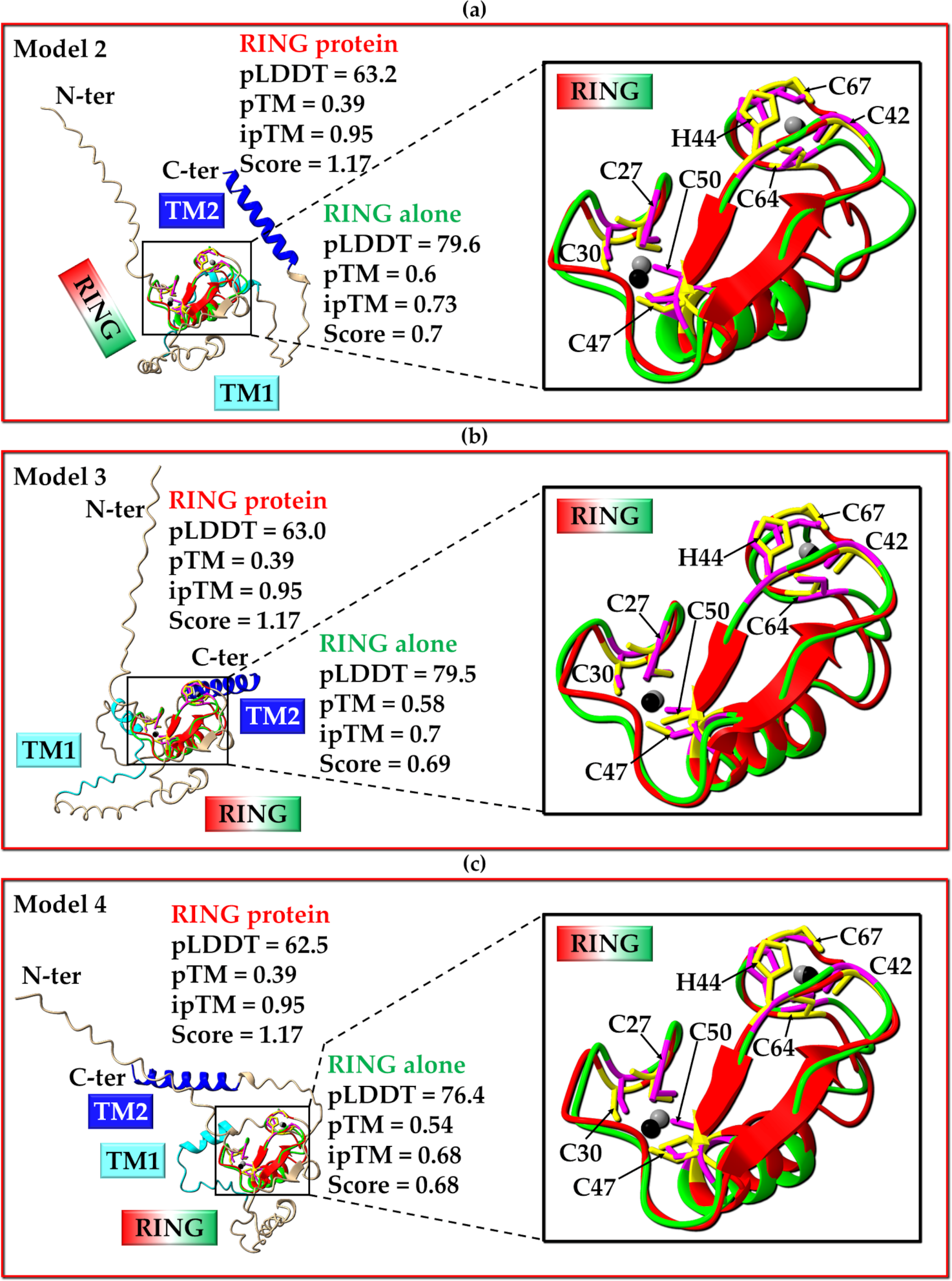


**
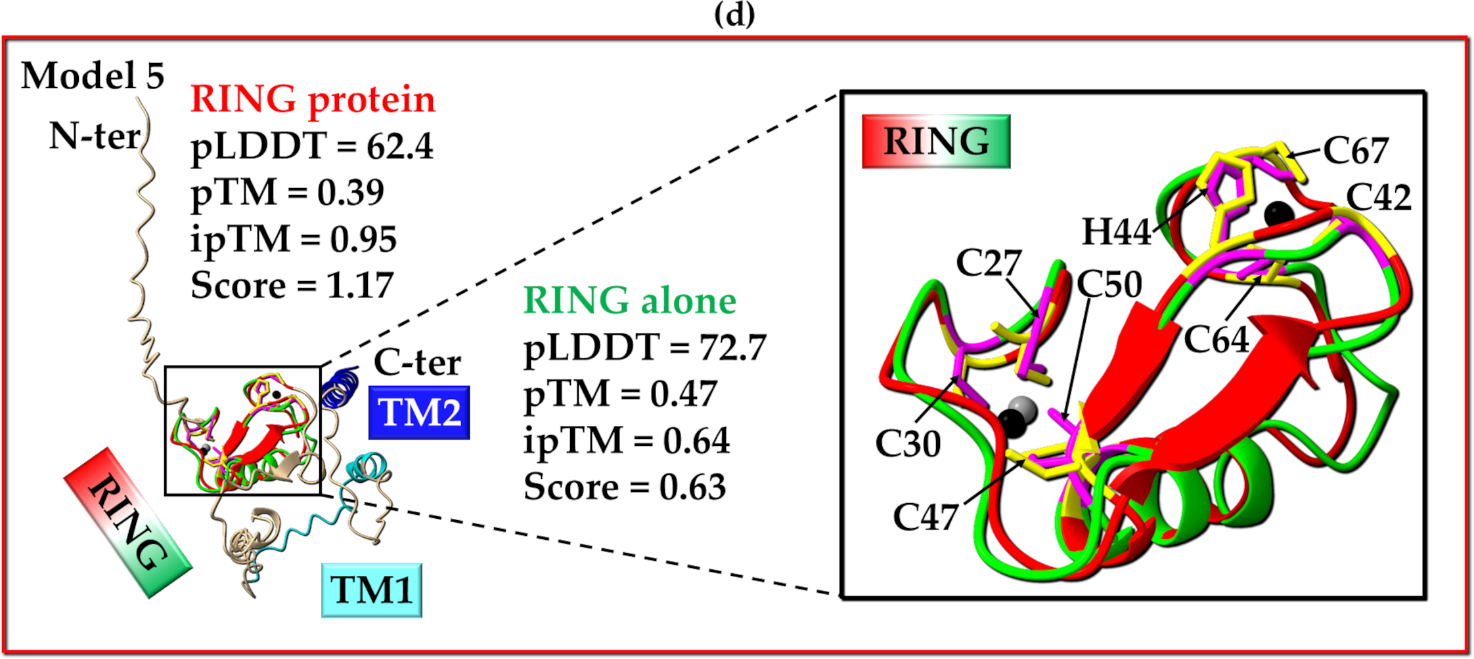
**

**Fig. S5.** (a-d) Comparison of best AF3 [51,52] models (from 2^nd^ to 5^th^ ranked structures) predicted in presence of two Zn^2+^ ions for the entire RNF5 protein and the isolated RING domain. The RNF5 protein is reported with the TM1 (a.a. G118-F138) and TM2 (a.a. S160-I180) regions colored cyan and blue, respectively, and with the RING domain (a.a. C27-K68) colored red and overlayed to the AF3 model of the isolated RING domain (green). RING domain superpositions featured on the left side are shown in a zoomed view in the right panels. The RING domain models have been overlayed on the Cα atoms of the residues from C27 to K68 with Chimera X version 1.5 [60]. Residues contributing to the two Zn^2+^ coordination clusters (HC3, i.e., H44, C42, C64 and C67 and C4, i.e., C27, C30, C47 and C50) of the RING finger region, in the “alone” or “within the entire protein” forms, are colored magenta and yellow, respectively. Zn^2+^ ions are colored black when inserted in the entire protein, and grey when in the RING domain alone. The confidence scores (i.e., pLDDT, pTM and ipTM) along with the AF3 ranking scores used to sort the best AF3 models are indicated [51,52,107].

**Table S1.** RMSD (Root Mean Square Deviation) values calculated between the best AF2 and AF3 models of the RNF5 RING domain (residues C27-K68 from UniProt [57] entry Q99942), TM1 (residues G118-F138 from UniProt entry Q99942), TM2 (residues S160-I180 from UniProt entry Q99942), and the G118-I180 segment of RNF5 (UniProt entry Q99942) including both the TM1 and the TM2 regions of the protein, predicted as isolated protein domains. In all models the RMSD values were calculated with Chimera X version 1.5 [60] by superimposing corresponding structures on the backbone atoms (N, Cα, C, O) of all residues from corresponding domains or also, regarding the RING domain, just the ordered secondary structure elements.

| **RNF5 regions**  **AF models** | **RMSD (Å)**  **Single regions** |
| --- | --- |
| RING domain | 2.629 / 1.700* |
| TM1 | 11.157 |
| TM2 | 0.714 |
| TM1+TM2 | 10.788 |

* = the RMSD value was calculated by superimposing corresponding structures on the backbone atoms (N, Cα, C, O) of all residues involved in secondary structure elements (i.e., β1 from A37 to V39, β2 from L45 to C47, α1 from W48 to T57, UniProt code Q99942) within the RNF5 RING domain.

**Table S2.** RMSD values calculated between each of the five AF3 models obtained for RNF5 RING domain alone and within the entire RNF5 protein. The RMSD values were calculated with Chimera X version 1.5 [60] by superimposing corresponding structures on the backbone atoms (bb) (N, Cα, C, O) of all residues of the RNF5 RING domain (C27-K68, UniProt [57] code Q99942) (2^nd^ column) and on the backbone atoms (N, Cα, C, O) of all residues involved in secondary structure elements (i.e., β1 from A37 to V39, β2 from L45 to C47, α1 from W48 to T57, UniProt code Q99942) within the RNF5 RING domain (3^rd^ column).

| **RNF5 RING domain** | **RMSD (Å)**  **All bb** | **RMSD (Å)**  **Secondary structure** |
| --- | --- | --- |
| Model 1 | 2.498 | 1.198 |
| Model 2 | 2.456 | 1.513 |
| Model 3 | 1.064 | 0.542 |
| Model 4 | 1.410 | 1.187 |
| Model 5 | 1.559 | 0.959 |


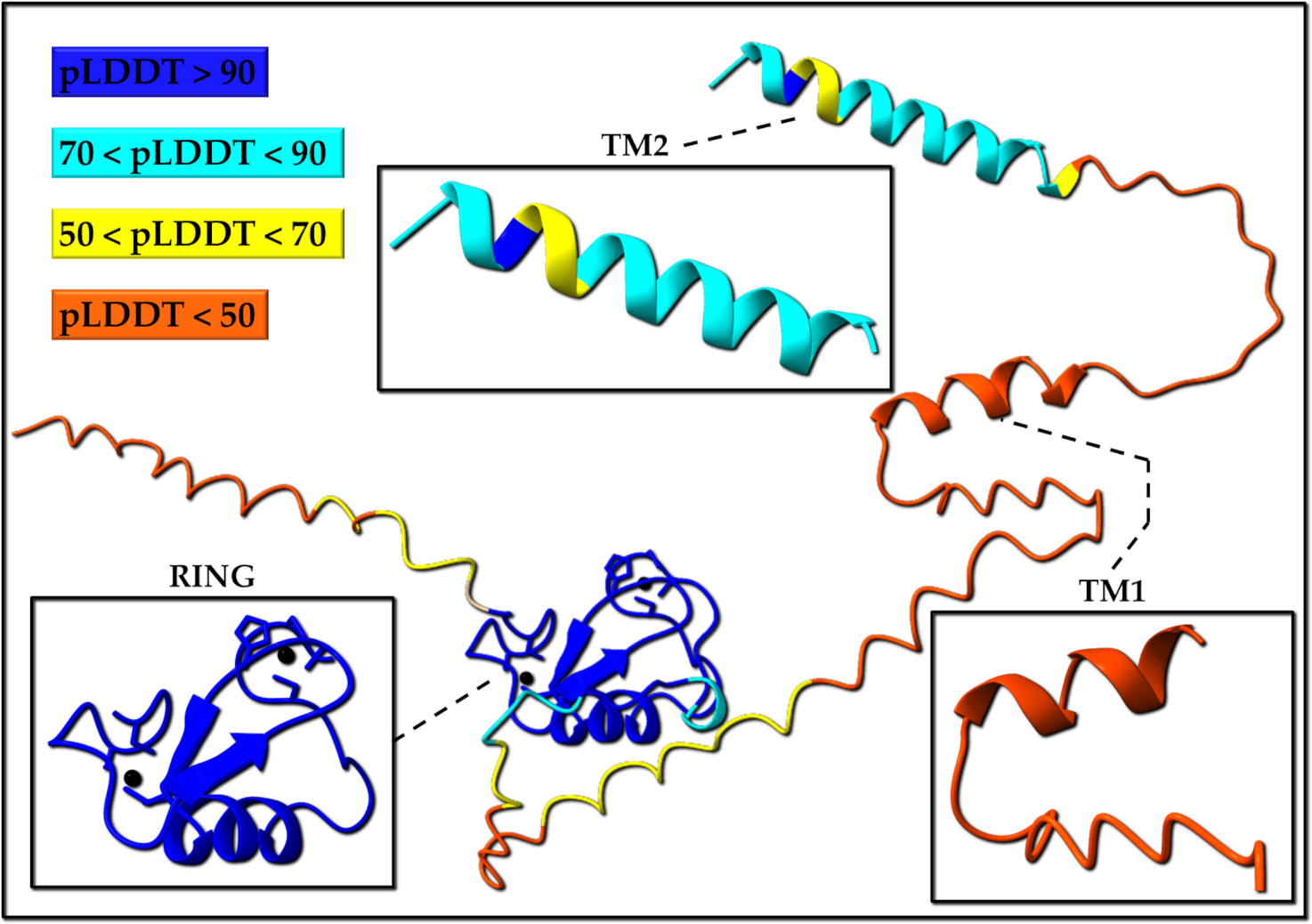


**Fig. S6.** Best AF3 [51,52] model, including two coordinating Zn^2+^ ions within the RING domain, of the RNF5 protein (residue range 1-180 from UniProt [57] entry Q99942). The atomic coordinates are represented in ribbon drawing with each amino acid colored according to the confidence level of the pLDTT score (See color legend on the top left side: blue stands for highly confident prediction, cyan: confident prediction, yellow: low accuracy prediction, orange: very low confident prediction [102]).

**
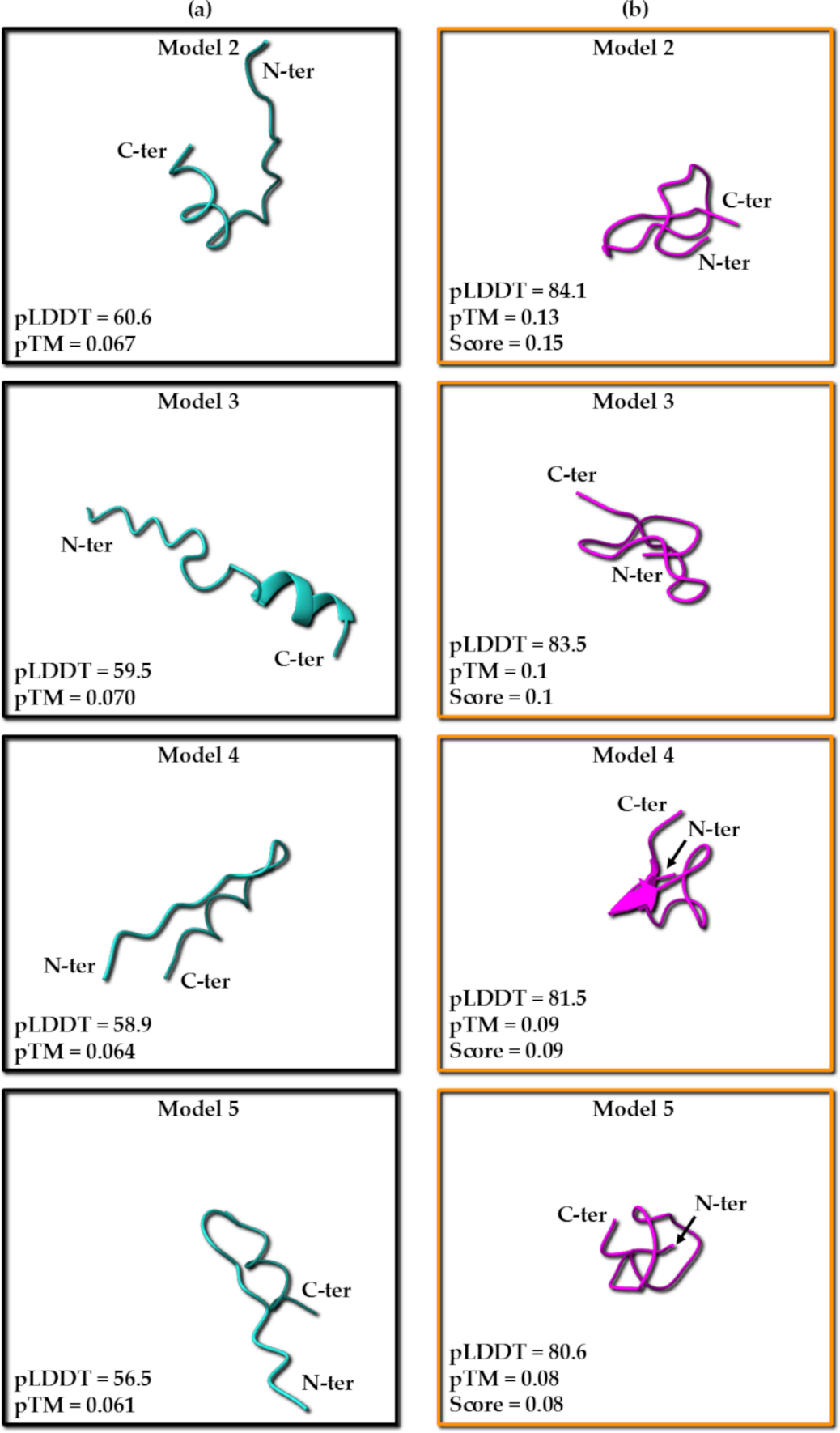
Fig. S7.** (a) AF2 [49,55] (light sea green) and (b) AF3 [51,52] (magenta) models (from 2^nd^ to 5^th^ ranked structures) of the first transmembrane (TM1) region of the RNF5 protein (residues G118-F138 from UniProt [57] entry Q99942). The pLDDT, and pTM scores [59,107,108] of the models are indicated in each (a) and (b) panel along with the AF3 scores [51,52] used to rank the best models that are indicated in the (b) column.

**
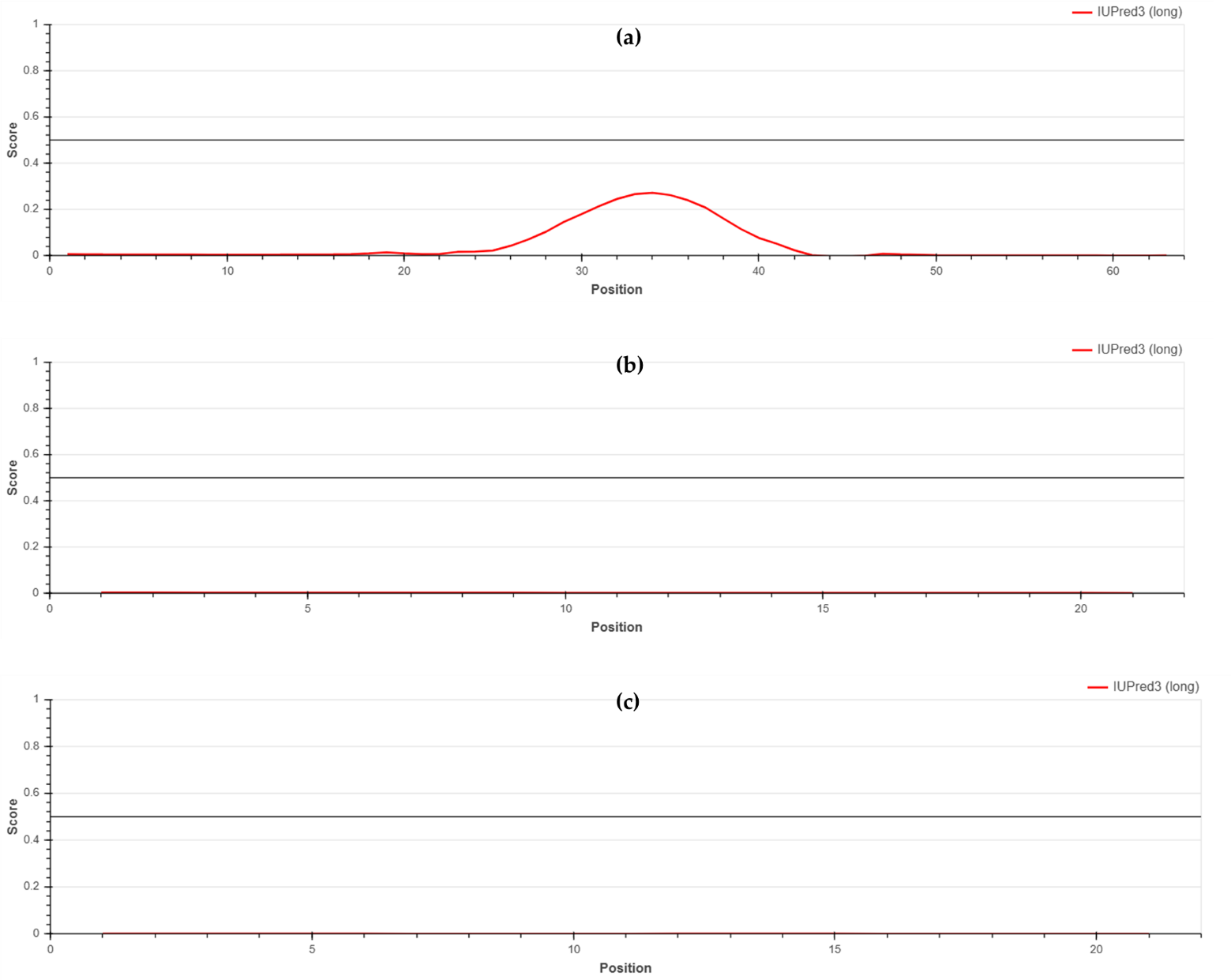
Fig. S8.** IUPred3 (Intrinsically Unstructured Prediction3) [110] disorder prediction for (a) the TM1 + TM2 region of human RNF5 protein (i.e., residues 118-180, UniProt [57] entry Q99942); (b) TM1 region (i.e., residues 118-138, UniProt entry Q99942); (c) TM2 region (i.e., residues 160-180, UniProt entry Q99942). Predictions were run through the IUPred3 webserver (https://iupred3.elte.hu/ access date 24/02/2026) by employing the medium smoothing and IUPred3 long Disorder options. A score close to zero indicates a well folded globular protein; scores higher than 0.5 point out the possible presence of intrinsically disordered regions (higher confident predictions are associated with scores closer to 1).

**
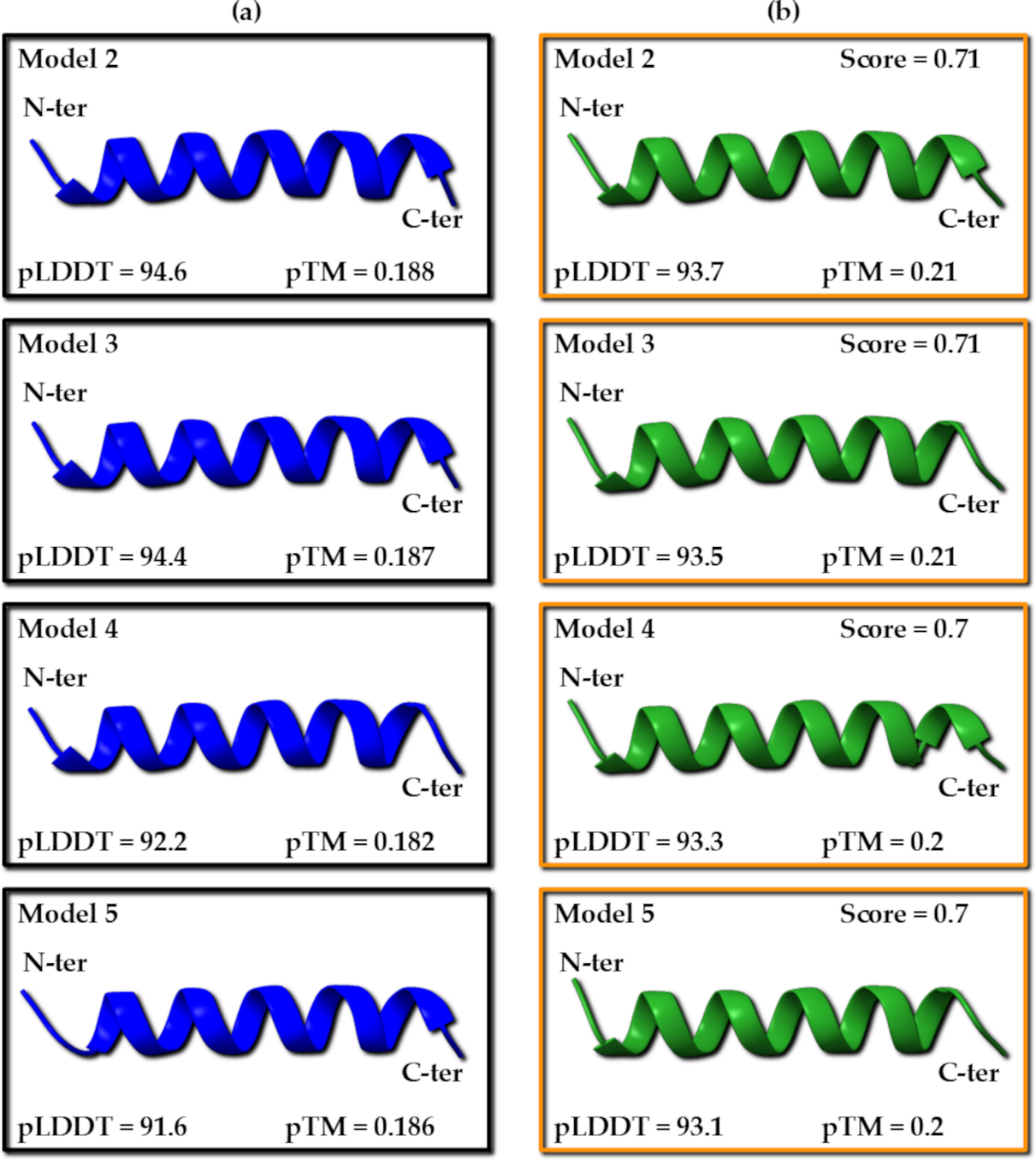

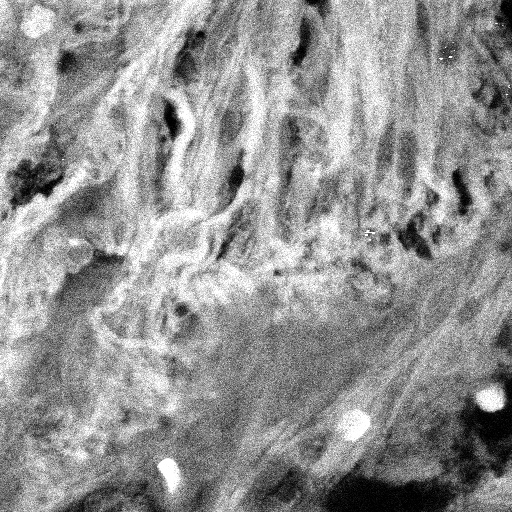
Fig. S9.** (a) AF2 [49,55] (blue) and (b) AF3 [51,52] (green) models (from 2^nd^ to 5^th^ ranked structures) of the second transmembrane (TM2) region of the RNF5 protein (residues S160-I180 from UniProt [57] entry Q99942). The pLDDT, and pTM scores [59,107,108] are indicated for all models along with the AF3 ranking scores [51,52].

**H**

**T**

**
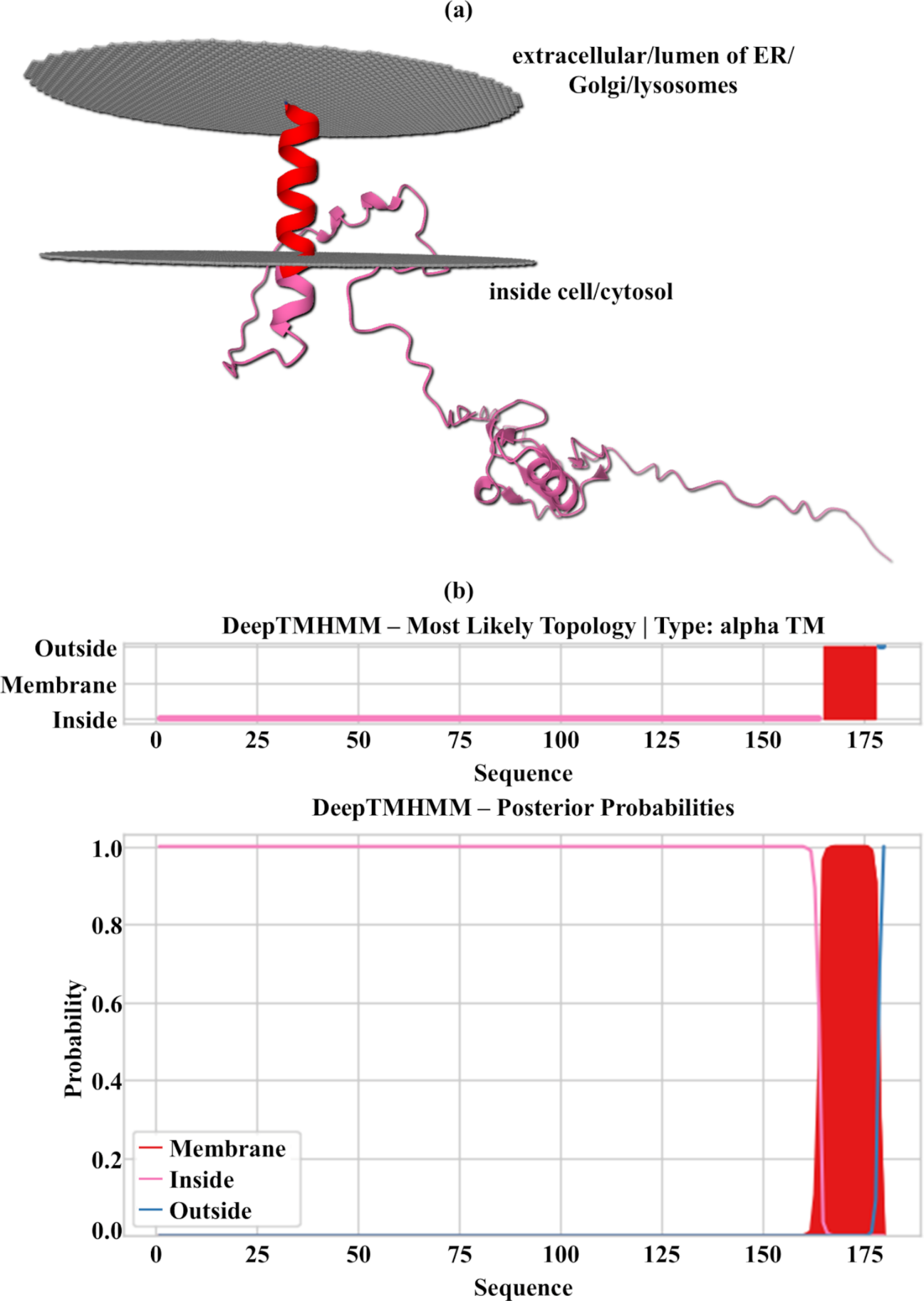
**

**Fig. S10.** Results from the webserver MembraneFold [112] (<https://biolib.com/KU/MembraneFold/>, access date 10 June 2026) obtained from the sequence of the entire RNF5 protein (residues M1-I180 from UniProt entry Q99942 for human RNF5). (a) Structure of RNF5-TM2 showing its positioning within the membrane. (b) DeepTMHMM (**Deep** Learning **T**rans**m**embrane **H**elices Hidden **M**arkov **M**odel (or **M**ethod)) [113] topology prediction with the most likely topology output is reported in the upper panel and the Posterior Probabilities output in the lower panel.

**
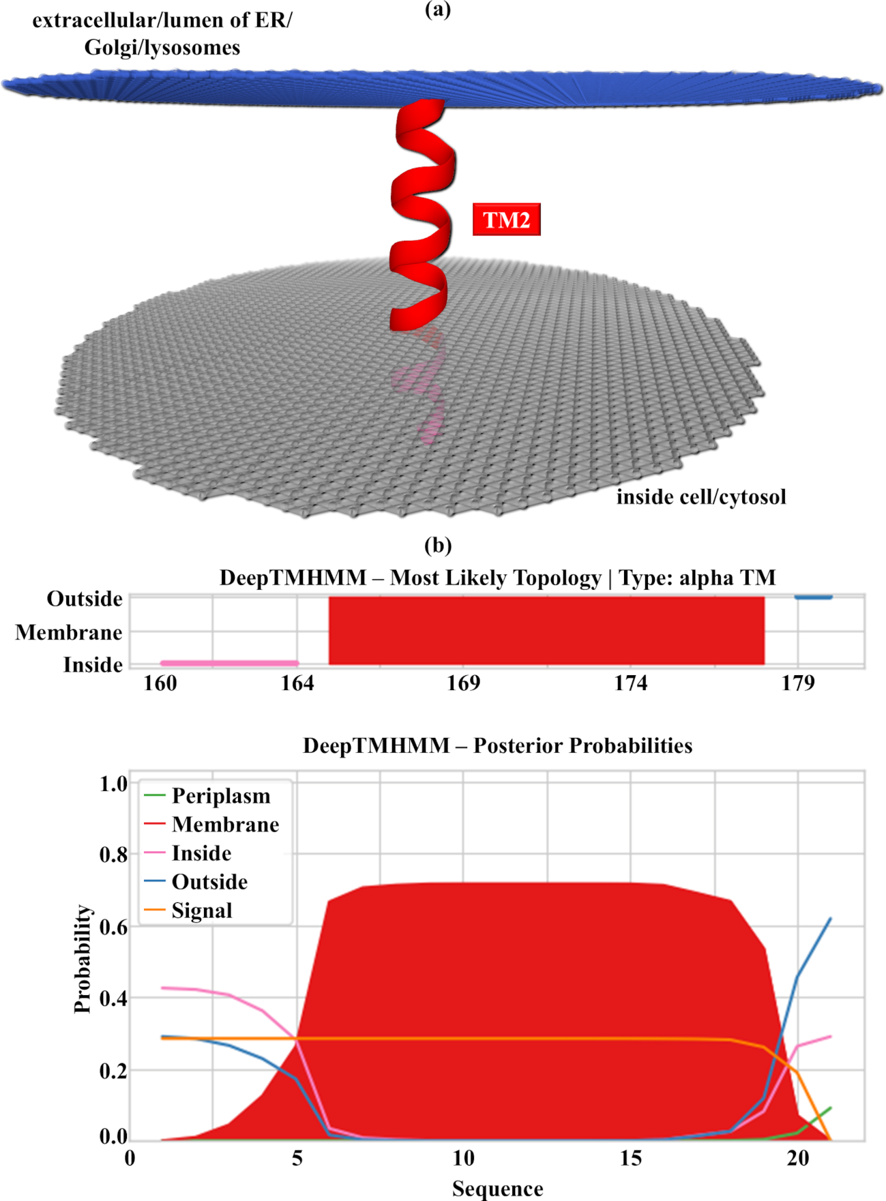
**

**Fig. S11.** Results from the webserver MembraneFold [112] obtained from the sequence of the RNF5-TM2 (residue S160-I180). (a) Structure of RNF5-TM2 and its positioning in the membrane. (b) DeepTMHMM [113] topology predictions with the Most Likely Topology output in the upper panel and the Posterior Probabilities output in the lower panel.

**
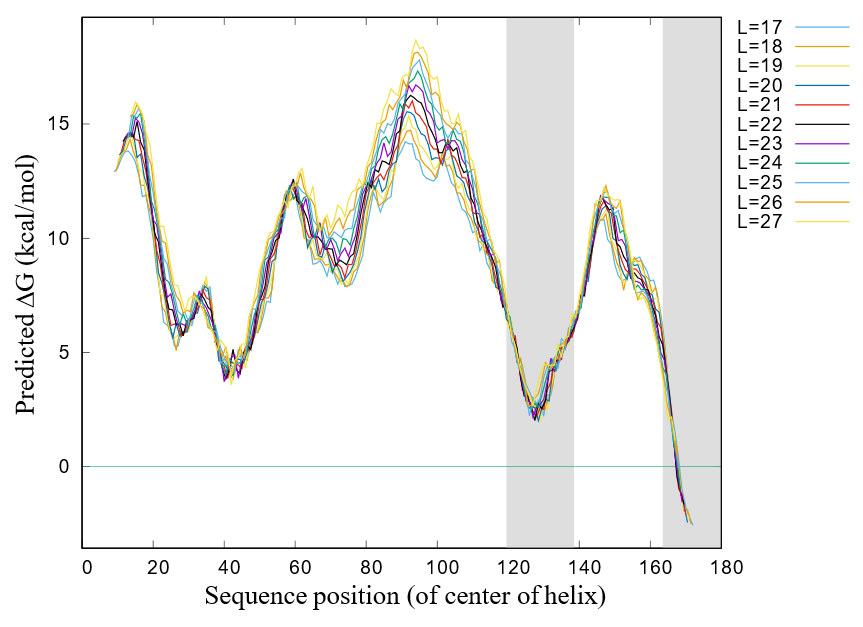
Fig. S12.** Prediction of TransMembrane (TM) helices in protein sequences through the ΔG prediction server [114]. The ΔG prediction server (<https://dgpred.cbr.su.se/index.php?p=fullscan->, Access date 11/06/2026) was used to predict helical TM regions in the RNF5 sequence. The sequence of the RNF5 protein (M1-I180 from UniProtKB entry Q99942) was used as input for the “Full protein scan” mode and other default parameters were implemented (i.e., Helix min length:17; Helix max length: 27; Length correction: Yes). The ΔG prediction server scans the input sequence of a protein using a sliding window of different residue lengths. The output is a prediction of the ΔG_app_ (apparent free energy difference) for insertion by the Sec61 translocon of the different protein stretches into the ER membrane. The ΔG_app_ is calculated by considering the energetic contributions from each amino acid based on its specific position within the primary sequence, the contribution of the hydrophobic moment, and the length of the analyzed sequence_._ Most negative ΔG_app_ values are related to the prediction of TM insertion of a protein sequence.


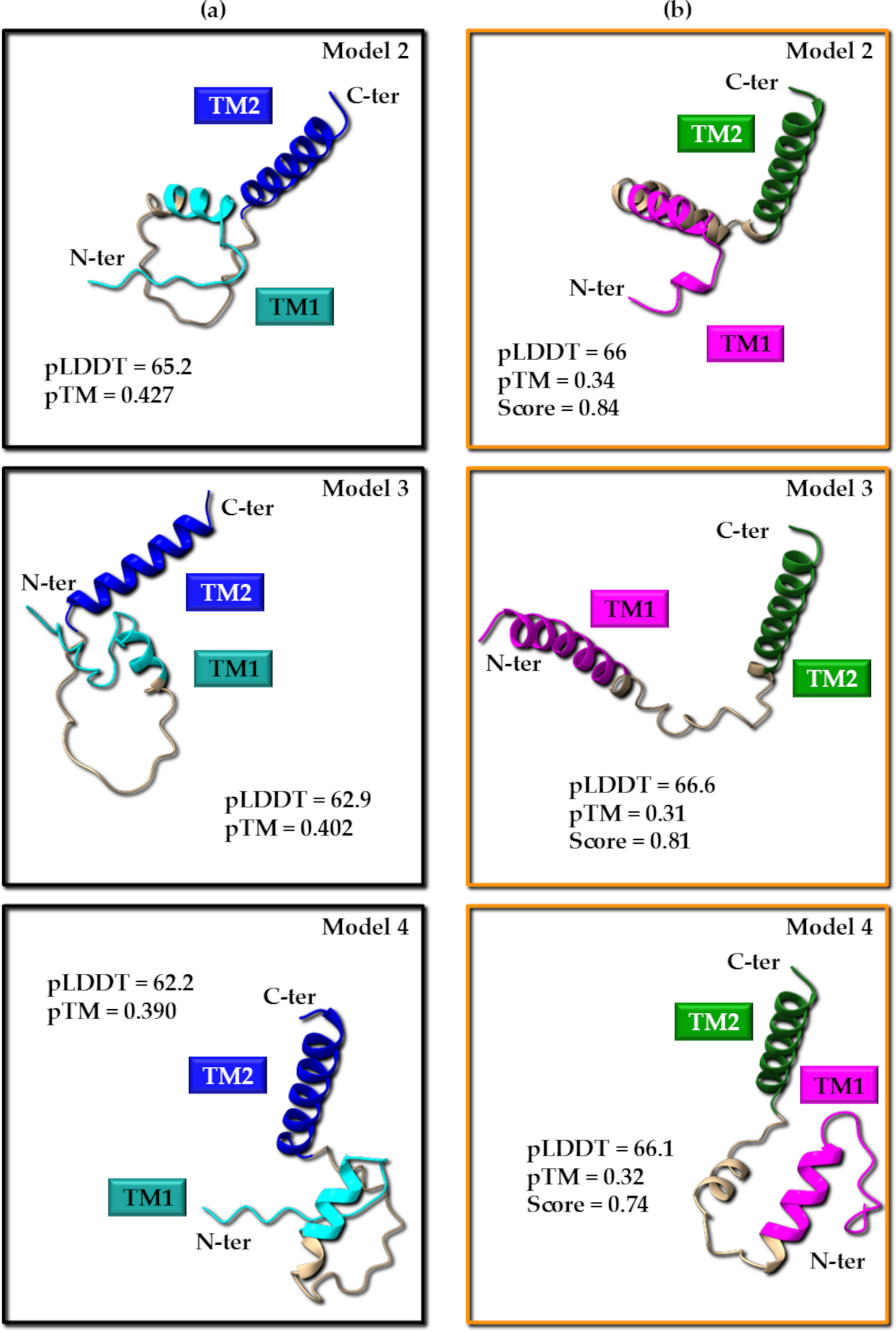


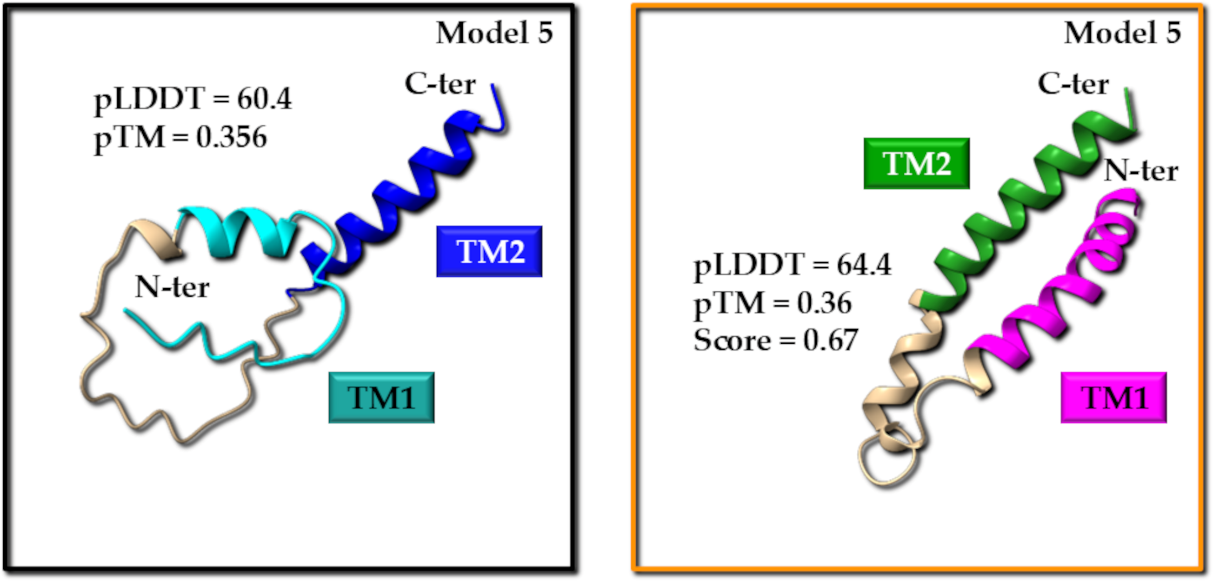
**Fig. S13.** (a) AF2 [49,55] and (b) AF3 [51,52] models (from 2^nd^ to 5^th^ ranked structures) of the G118-I180 region of RNF5 (UniProt [57] entry Q99942) including the first transmembrane (TM1) segment of the protein (residues G118-F138, cyan in AF2 models and magenta in AF3 models) and the second transmembrane (TM2) segment (S160-I180, blue in AF2 models and green in AF3 models). pLDDT, pTM and AF3 ranking scores of the 4 models are indicated [52,59,107,108].

**Table S3.** EphA2-Sam/RNF5 complex: analysis of LigPlot+ [63,64] intermolecular H-bonds and non-bonded interactions provided by different RNF5 residues in each of the 5 best AF2 [49,55] models of the complex between the whole RNF5 protein and EphA2-Sam. The number of contacts provided by EphA2 residues positioned inside the EH interface over the total number of EphA2-Sam provided interactions (EphA2-Sam EH contacts/Total number of contacts) is reported for each model in the last row, as well.

| **RNF5**  **residues** | **Number of**  **H-bonds** | | | | | **Number of**  **non-bonded interactions** | | | | |
| --- | --- | --- | --- | --- | --- | --- | --- | --- | --- | --- |
|  | **Model 1** | **Model 2** | **Model 3** | **Model 4** | **Model 5** | **Model 1** | **Model 2** | **Model 3** | **Model 4** | **Model 5** |
| E26 | ---------- | ---------- | 1 | 1 | 2 | ---------- | ---------- | 5 | 3 | 8 |
| I29 | 1 | 2 | ---------- | ---------- | ---------- | 4 | 10 |  | 6 | ---------- |
| C30 | ---------- | ---------- | 1 | ---------- | 2 | 7 | 8 | 12 | 8 | 5 |
| L31 | ---------- | ---------- | ---------- | 2 | ---------- | 20 | 7 | 15 | 24 | 15 |
| E32 | 1 | 1 | 2 | ---------- | 2 | 3 | 4 | 4 | 2 | 14 |
| Q53 | ---------- | 1 | ---------- | ---------- | ---------- | 1 | 5 | ---------- | 6 | ---------- |
| W54 | ---------- | ---------- | ---------- | ---------- | ---------- | 2 | 34 | ---------- | 3 | ---------- |
| Q57 | ---------- | 1 | ---------- | ---------- | ---------- | ---------- | 57 | ---------- | ---------- | ---------- |
| R58 | ---------- | 3 | ---------- | ---------- | ---------- | ---------- | 21 | ---------- | ---------- | ---------- |
| **EphA2-Sam EH contacts / Total number of contacts** | 2/2 | 3/8 | 4/4 | 3/3 | 6/6 | 34/37 | 68/93 | 36/36 | 49/52 | 38/42 |

**
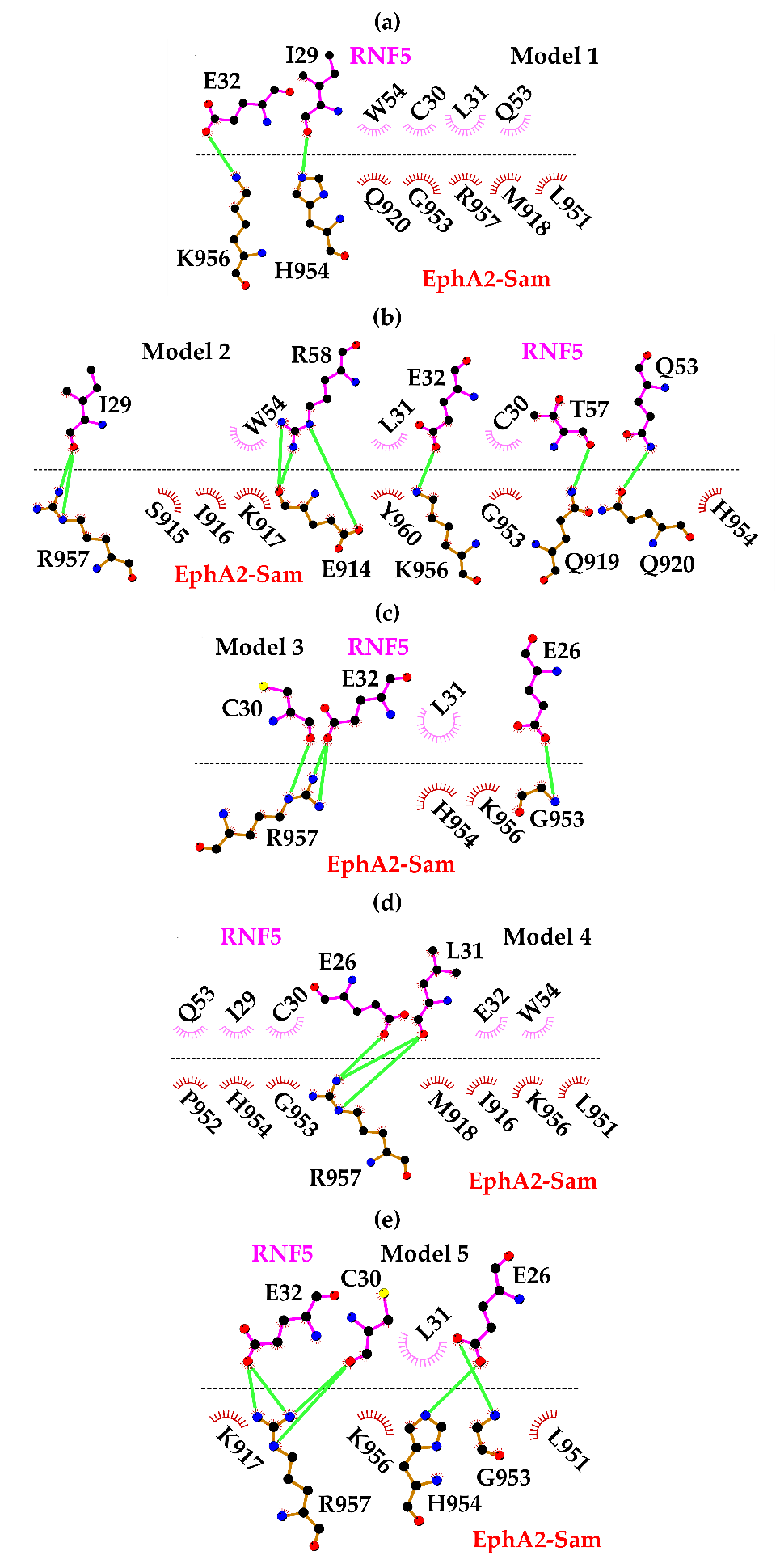
Fig. S14.** 2D diagrams of intermolecular interactions generated by LigPlot+ [63,64] for the five AF2 [49,55] models predicted for EphA2-Sam in complex with the entire RNF5 protein. (a) 1^st^ ranked, (b) 2^nd^ ranked, (c) 3^rd^ ranked, (d) 4^th^ ranked and (e) 5^th^ ranked. Residues involved in non-bonded interactions are labelled and represented by red and magenta crescents with bristles. Green lines indicate H-bonds.


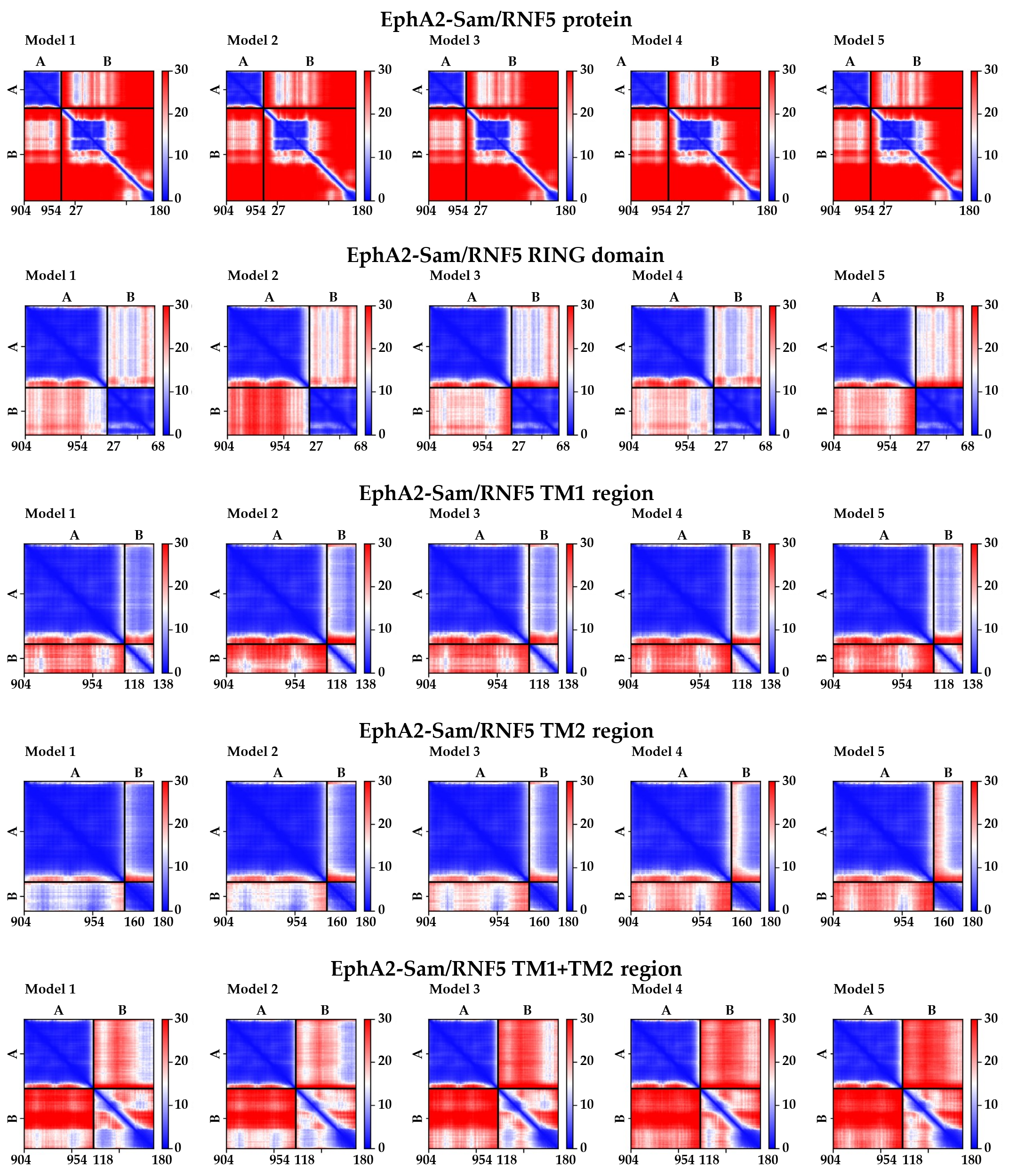


**Fig. S15.** PAE (Predicted Aligned Error) plots relative to the five AF2 models of the complex of EphA2-Sam (chain A in each row) with the entire RNF5 protein (chain B in the 1^st^ row), with the RNF5 RING domain (chain B in the 2^nd^ row), with the RNF5 TM1 region (chain B in the 3^rd^ row), with the RNF5 TM2 region (chain B in the 4^th^ row) and with the RNF5 TM1+TM2 region (chain B in the 5^th^ row). Each plot is flanked by a PAE score scale, where the values range from those <5 Å (dark blue) related to very low PAE and high confidence, to > 20 Å (dark red) related to high PAE and low confidence in the relative position of X and Y residues. In all plots the top-left to bottom-right regions represent intra-chain scores.

**Table S4.** RMSD values calculated between the best AF2 and AF3 models of EphA2-Sam in complex with either RNF5 RING domain (residues C27-K68 from UniProt [57] entry Q99942), or TM1 (residues G118-F138 from UniProt entry Q99942), or TM2 (residues S160-I180, from UniProt entry Q99942), or the G118-I180 region of RNF5 (UniProt entry Q99942) including both the TM1 region and the TM2 domains. The RMSD values were calculated with Chimera X version 1.5 [60] upon superimposing the backbone atoms (N, Cα, C, O) of all complex residues.

| **AF Complex** | **RMSD (Å)** |  |  |  |  |  |  |  |
| --- | --- | --- | --- | --- | --- | --- | --- | --- |
| EphA2-Sam/  RING domain | 8.376 |  |  |  |  |  |  |  |
| EphA2-Sam/  TM1 | 8.889 |  |  |  |  |  |  |  |
| EphA2-Sam/  TM2 | 9.685 |  |  |  |  |  |  |  |
| EphA2-Sam/  TM1+TM2 | 23.684 | 2 |  |  |  |  |  |  |


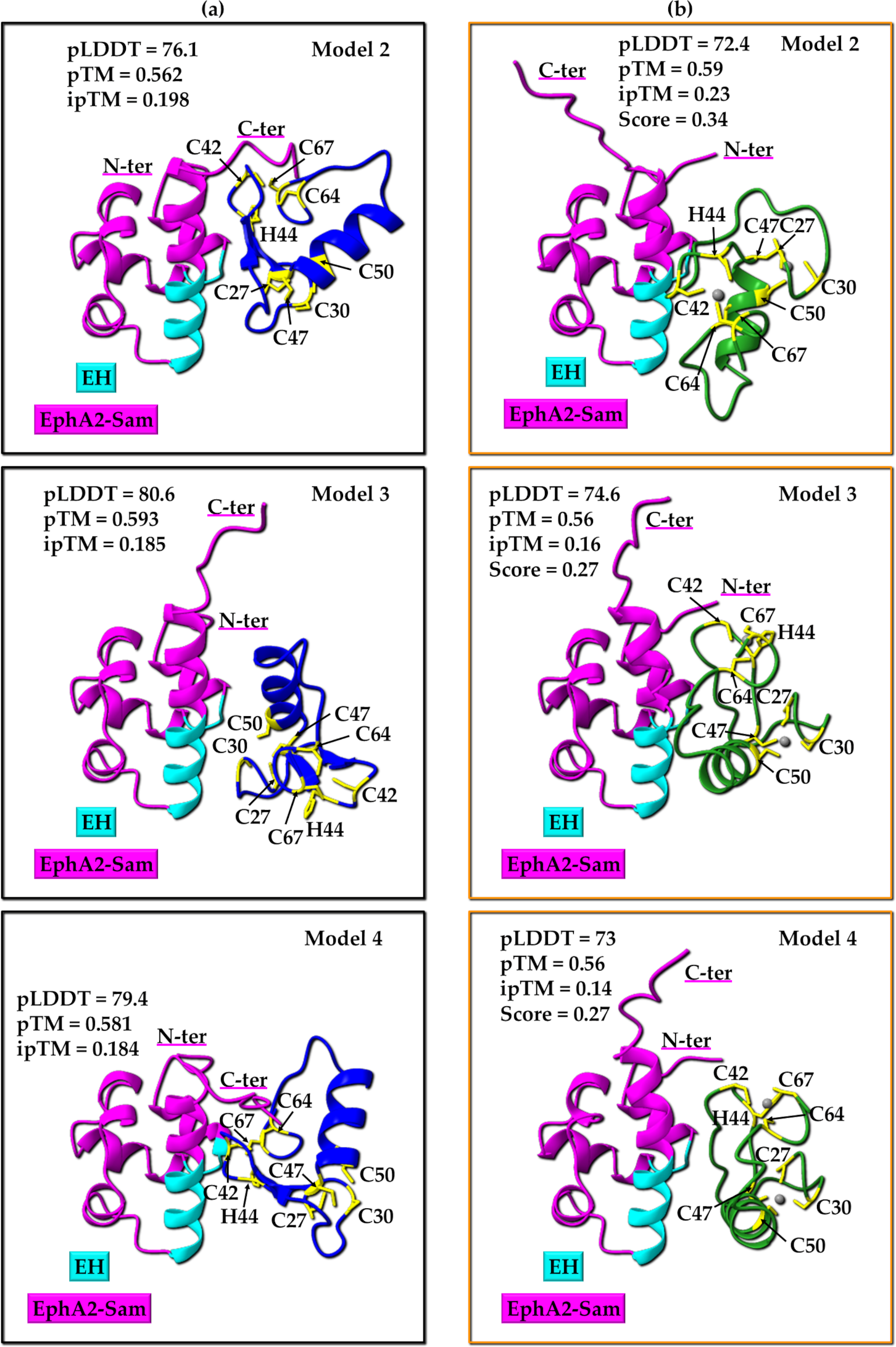


**
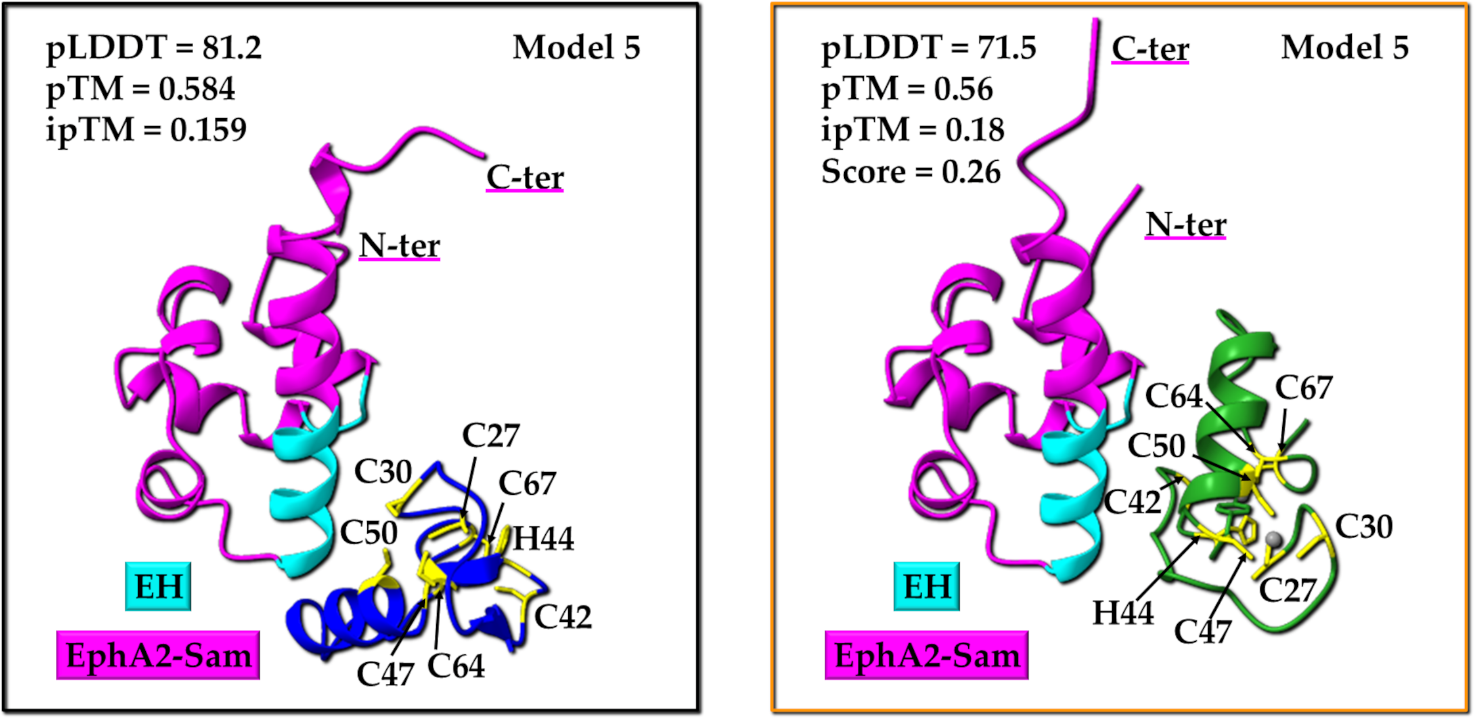
**

**Fig. S16.** (a) AF2 [49,55] models (from 2^nd^ to 5^th^ ranked structures) for EphA2-Sam (residues V904-I976 including the PBM (=PDZ Binding Motif) from the UniProt [57] entry P29317) in complex with the RING region of the RNF5 protein (residues C27-K68 from the UniProt entry Q99942). EphA2-Sam is shown in a ribbon representation (magenta) with the EH region colored cyan (residues I916-M918 and P952-Y960); the RNF5 RING domain is presented in blue. (b) AF3 [51,52] models (from 2^nd^ to 5^th^ ranked structures) for EphA2-Sam / RNF5 RING complex including Zn^2+^ ions. EphA2-Sam is colored as in (a) whereas, the RNF5 RING domain is shown in green. Yellow is used in (a) and (b) panels to highlight the backbone and side chains of the residues contributing to the HC3 (i.e., H44, C42, C64 and C67) and C4 (i.e., C27, C30, C47 and C50) Zn^2+^ coordination clusters. In panels (a,b) only the heavy atoms of the Zn^2+^ coordinating residues are shown. The pLDDT, pTM, ipTM confidence and the AF3 ranking scores of the 4 models are reported [52,59,107,108].


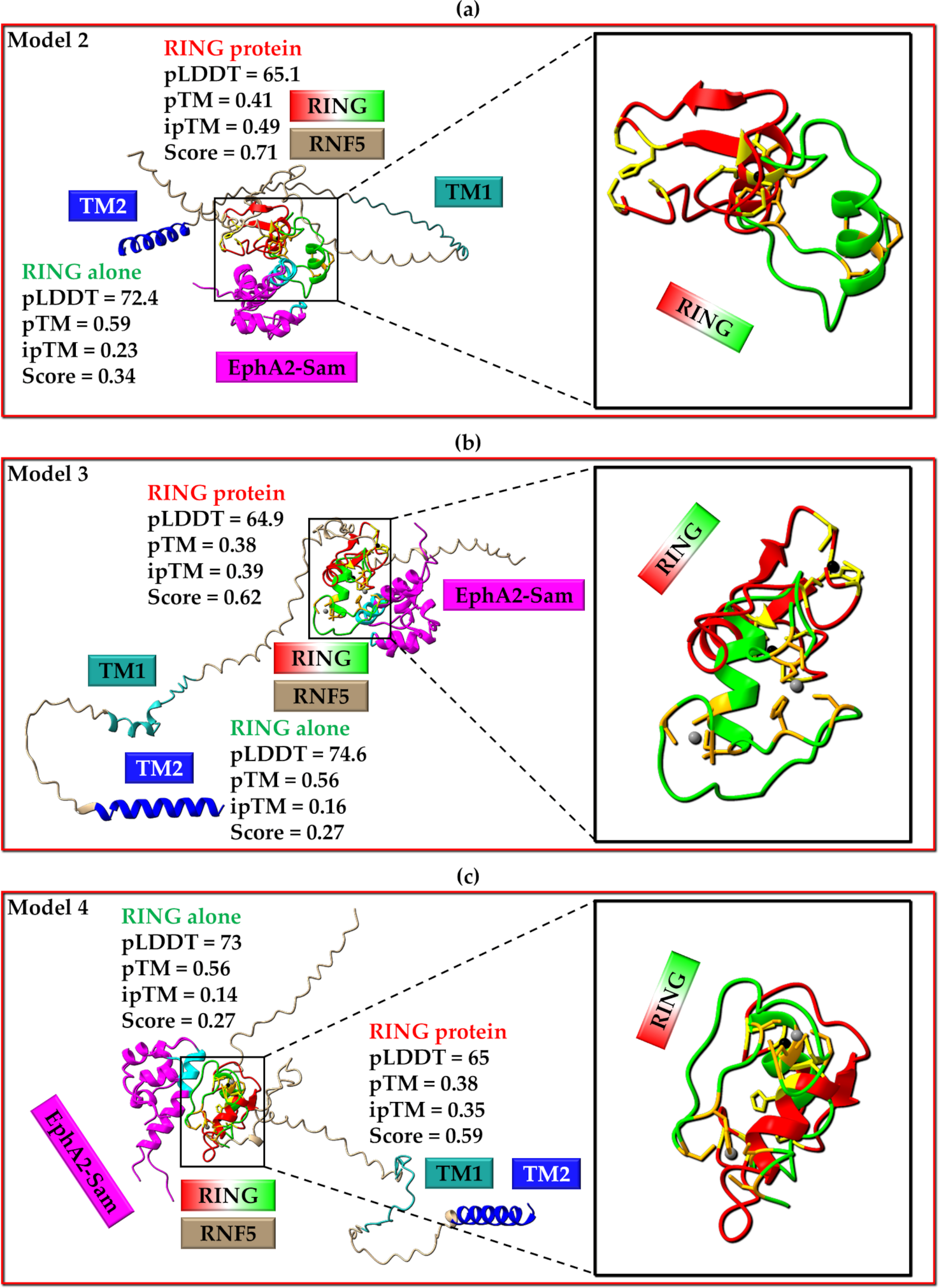


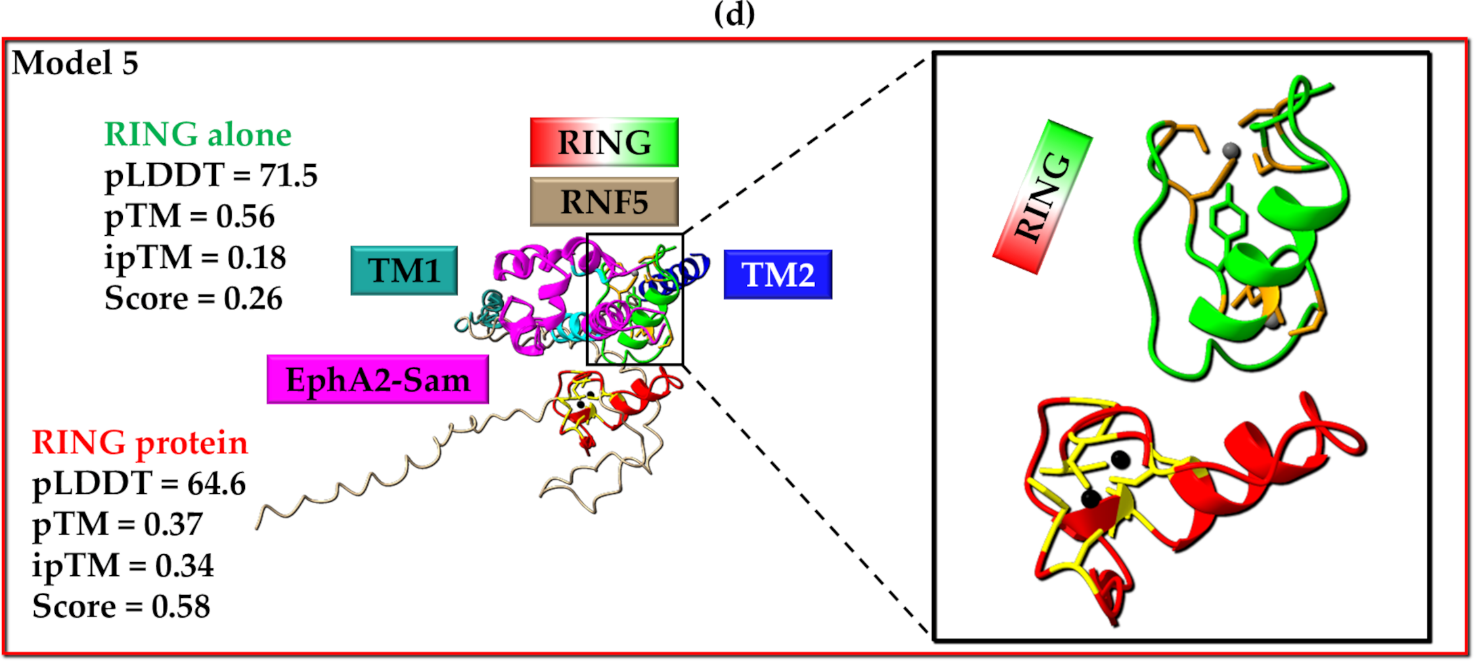


**Fig. S17.** (a-d) Comparison of the four AF3 predicted models (from 2^nd^ to 5^th^ ranked structures) [51,52] for EphA2-Sam (residues V904-I976 including the PBM from the UniProt [57] entry P29317) in complex with either the isolated RING region of the RNF5 protein (residues C27-K68 from UniProt entry Q99942) (light green) or the entire RNF5 protein (Residues M1-I180 from the UniProt entry Q99942) (red). Orange and yellow are used to highlight the residues contributing to the two Zn^2+^ coordinating clusters HC3 (i.e., H44, C42, C64 and C67) and C4 (i.e., C27, C30, C47 and C50) of the RING region, respectively, alone and within the entire protein. EphA2-Sam is shown in magenta with the EH interface (residues I916-M918 and P952-Y960) colored cyan. The RNF5 first (TM1) (residues G118-F138 from UniProt entry Q99942) and second (TM2) (residues S160-I180 from UniProt code Q99942) transmembrane regions are colored light sea green and blue, respectively. Models of EphA2-Sam/RNF5 and EphA2-Sam/RNF5 RING with the same AF ranking number, have been superimposed on the backbone Cα atoms of the EphA2-Sam domain with Chimera X version 1.5 [60]. The zoomed views of the RING region in each superimposition are enclosed within the right black boxes. The pLDDT, pTM and ipTM scores of the 4 models are indicated along with the AF3 ranking scores in each panel [51,52,107].

**Table S5.** RMSD values evaluated by comparing AF3 models of EphA2-Sam in complex with RNF5 RING domain alone and with the entire RNF5 protein. The RMSD values were calculated with Chimera X version 1.5 [60] and refer to overlays on the (N, Cα, C, O) backbone atoms of corresponding RNF5 RING and EphA2-Sam residues.

| **AF3 EphA2-Sam/**  **RNF5**  **AF3 EphA2-Sam/**  **RNF5 RING** | **RMSD (Å)** |
| --- | --- |
| Model 1 | 11.778 |
| Model 2 | 12.705 |
| Model 3 | 11.676 |
| Model 4 | 9.138 |
| Model 5 | 15.462 |


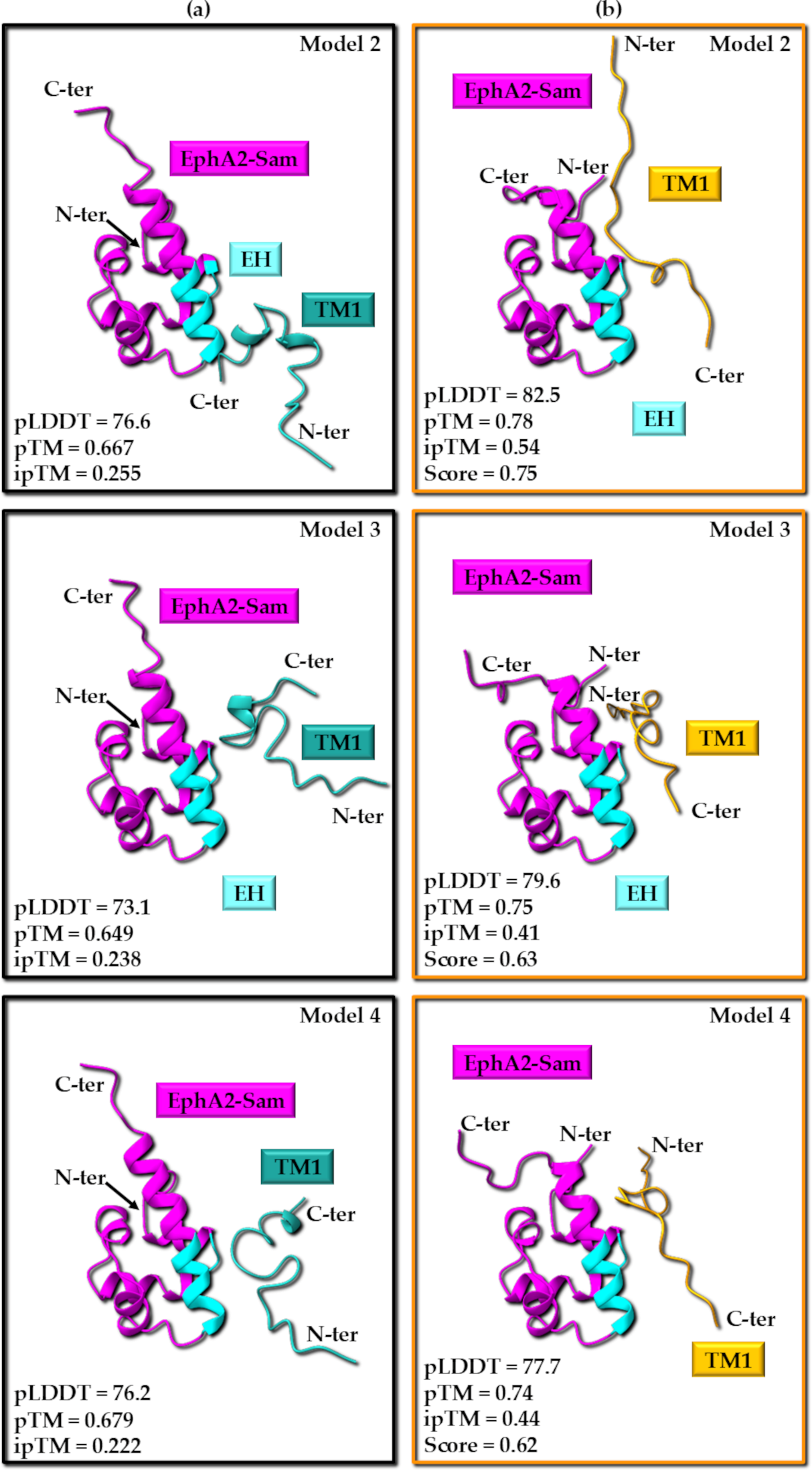


**
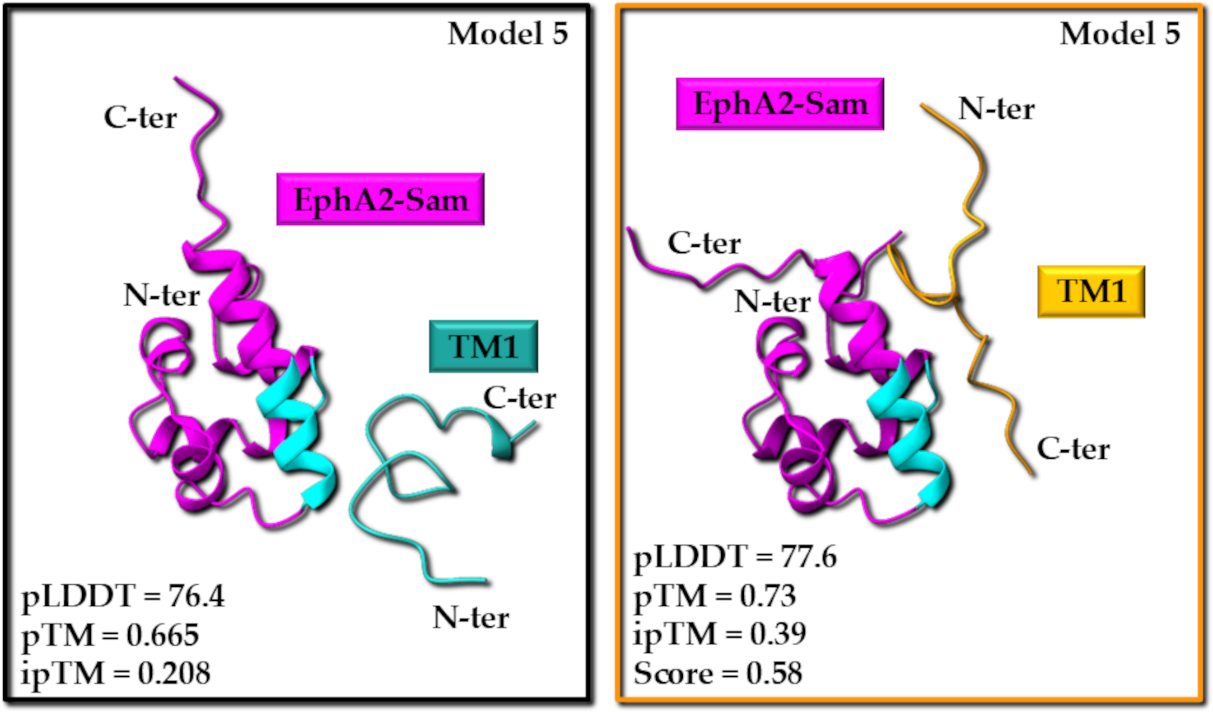
Fig. S18.** (a) AF2 [49,55] and (b) AF3 [51,52] models (from 2^nd^ to 5^th^ ranked structures) of EphA2-Sam (residues V904-I976 including the PBM from UniProt [57] entry P29317) in complex with the first transmembrane (TM1) region of the RNF5 protein (residues G118-F138 from the UniProt entry Q99942). In (a) and (b) panels EphA2-Sam is reported in magenta with the EH region (residues I916-M918 and P952-Y960) in cyan. The first transmembrane (TM1) region of the RNF5 protein is colored light sea green in (a) panels and orange in (b) panels. The pLDDT, pTM and ipTM scores along with the AF3 ranking scores are indicated for each model [52,59,107,108].


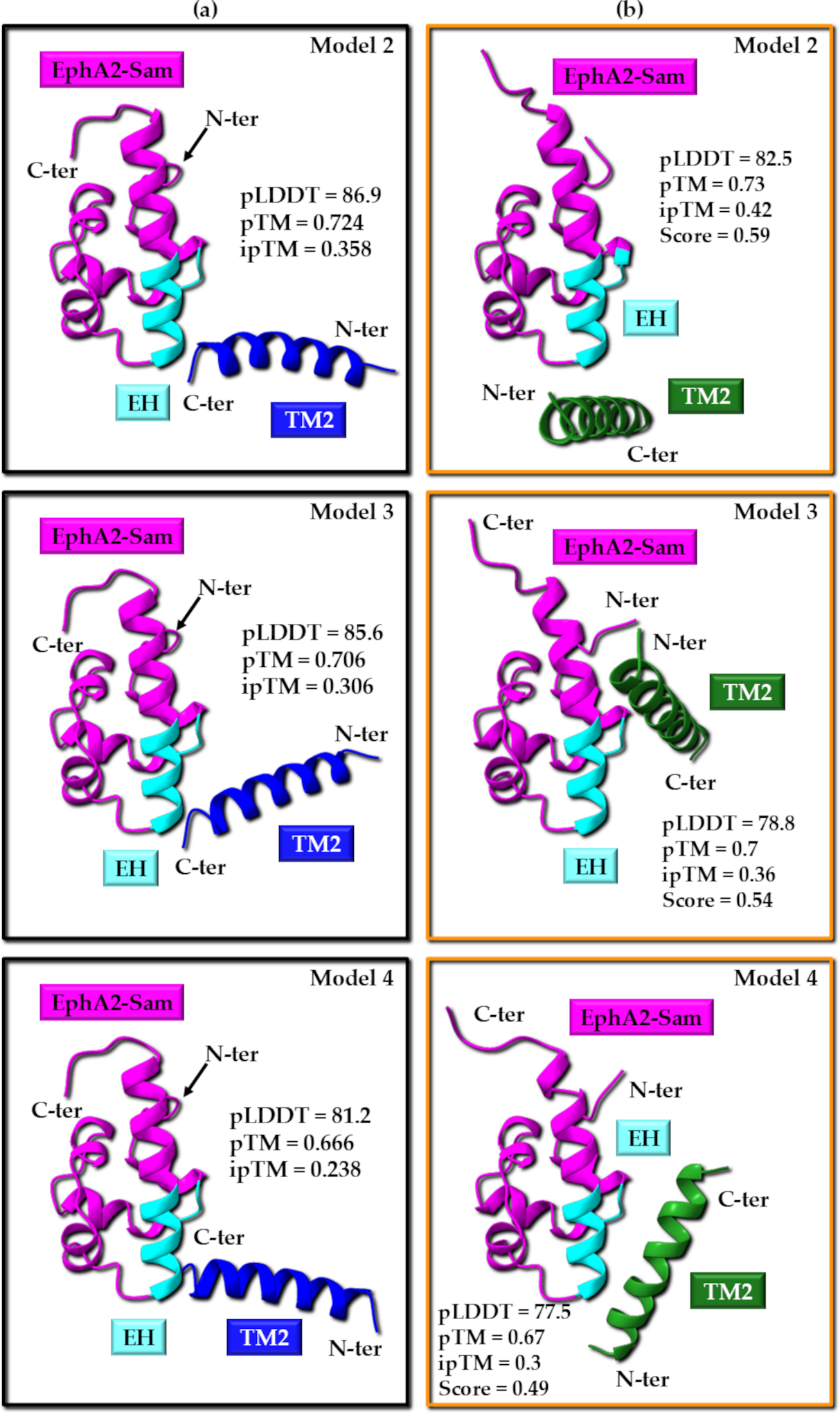


**
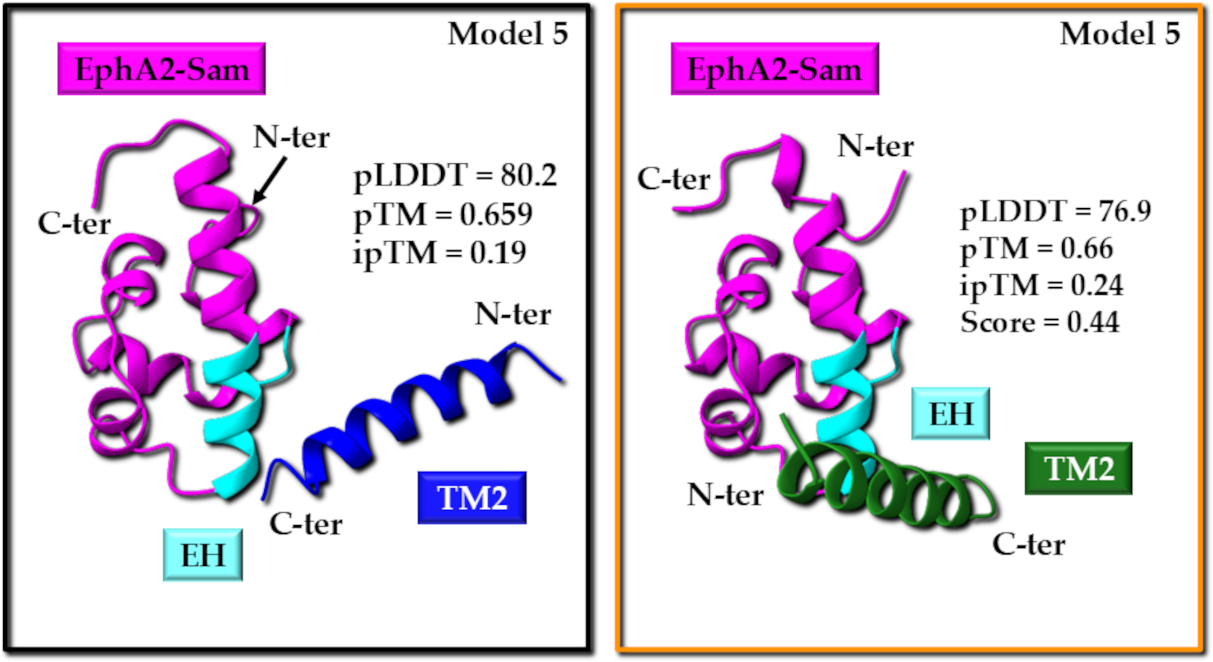
Fig. S19.** (a) AF2 [49,55] and (b) AF3 [51,52] models (from 2^nd^ to 5^th^ ranked structures) for EphA2-Sam (V904-I976 including the PBM, UniProt [57] entry P29317) in complex with the second transmembrane (TM2) region of the RNF5 protein (S160-I180, UniProt code Q99942). Magenta is used for EphA2-Sam, while the EH region (residues I916-M918, P952-Y960) is reported in cyan in (a) and (b) panels. Blue and green are used for the TM2 region of the RNF5 protein in (a) and (b) panels, respectively. The confidence pLDDT, pTM and ipTM scores of the 4 models are indicated in panels (a) and (b) while the AF3 ranking scores are reported in panels (b) as well [52,59,107,108].

**Fig. S20.** 2D diagrams of intermolecular interactions generated by LigPlot+ [63,64] for the five AF2 [49,55] models predicted for EphA2-Sam in complex with the RNF5 TM2 region. (a) 1^st^ ranked, (b) 2^nd^ ranked, (c) 3^rd^ ranked, (d) 4^th^ ranked and (e) 5^th^ ranked. Residues involved in non-bonded interactions are labelled and represented by red and magenta crescents with bristles. Green lines indicate H-bonds.


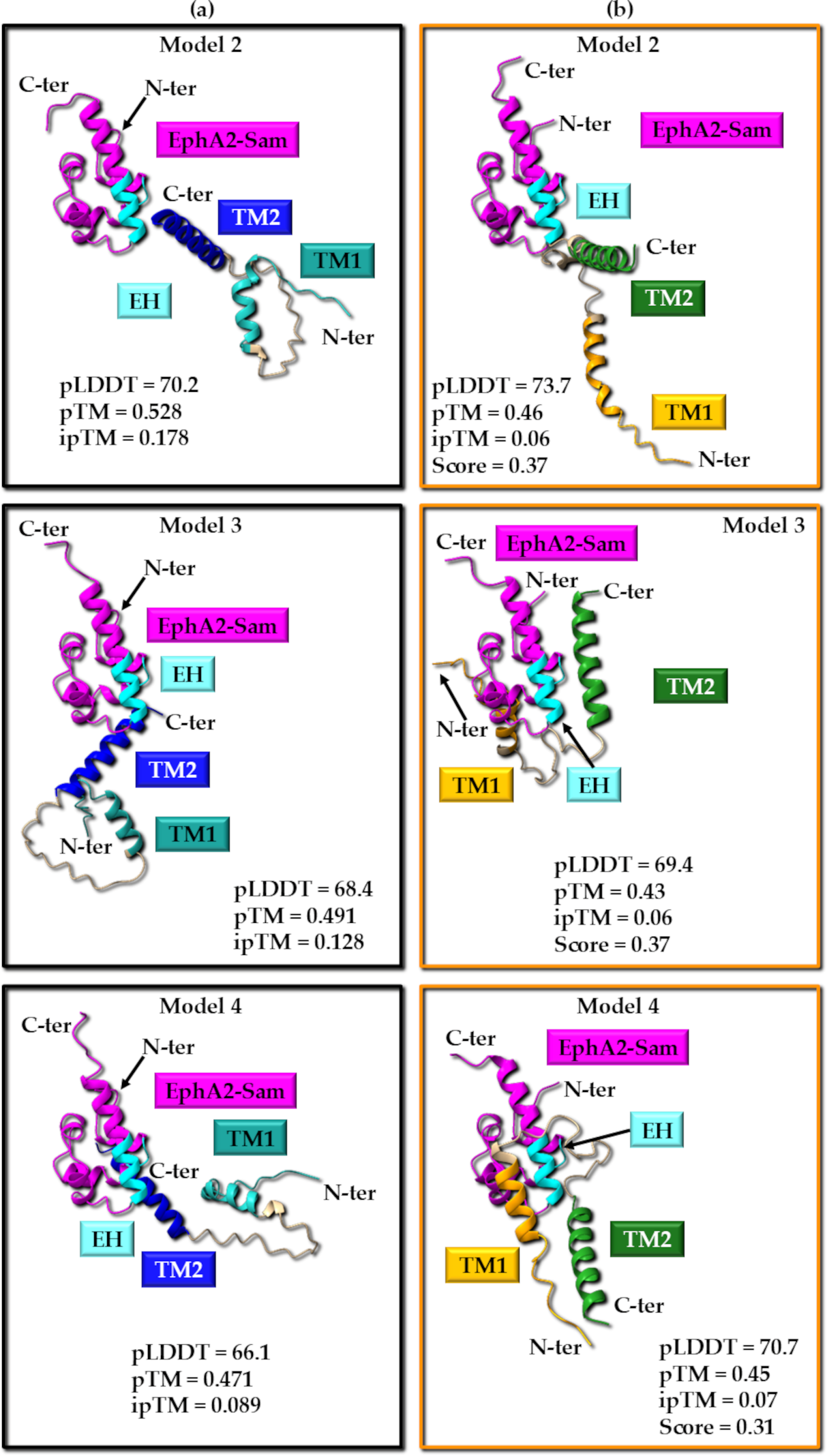


**
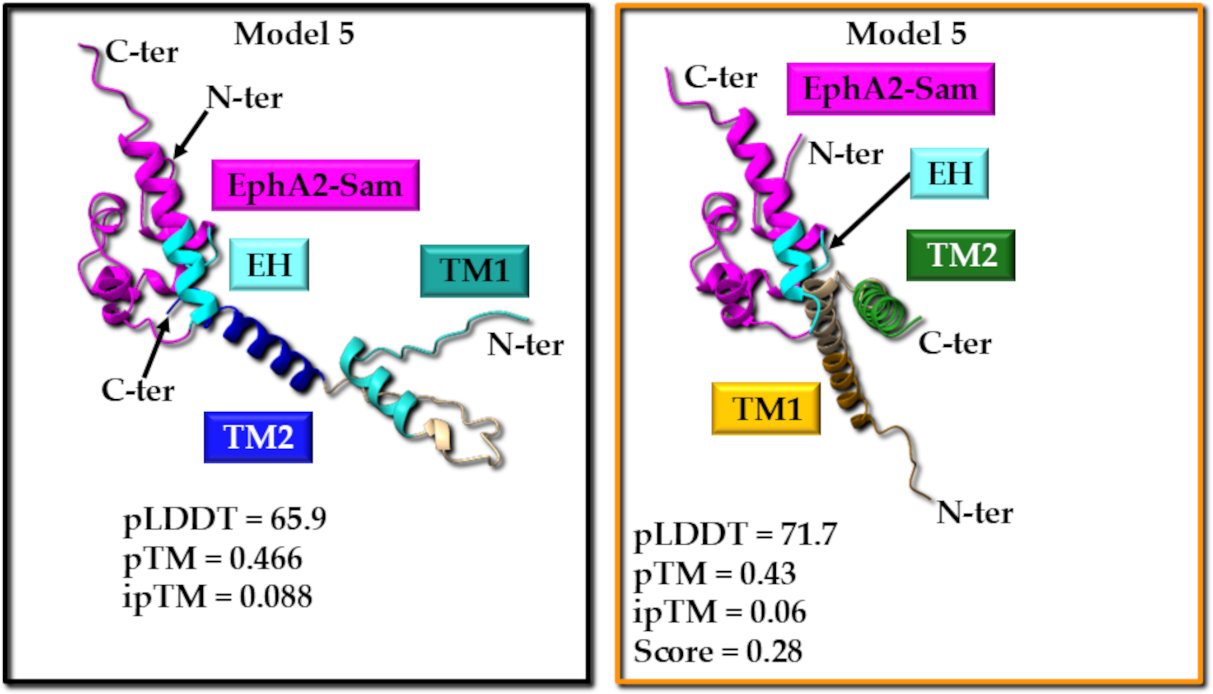
Fig. S21.** (a) AF2[49,55] and (b) AF3[51,52] models (from 2^nd^ to 5^th^ ranked structures) for EphA2-Sam (residues V904-I976 from the UniProt [57] entry P29317) in complex with the G118-I180 region of RNF5 (UniProt entry Q99942) including the first transmembrane segment (TM1) of the protein (residues G118-F138 from the UniProt entry Q99942, light sea green in AF2 models and orange in AF3 models) and the second one (TM2) (S160-I180, UniProt code Q99942, blue in AF2 models and green in AF3 models). In (a) and (b) panels EphA2-Sam is represented in magenta with the EH region (residues I916-M918, P952-Y960) in cyan. The pLDDT, pTM and ipTM scores of the 4 models are indicated in (a) and (b) whereas, AF3 ranking scores are reported in panels (b) [52,59,107,108].

*
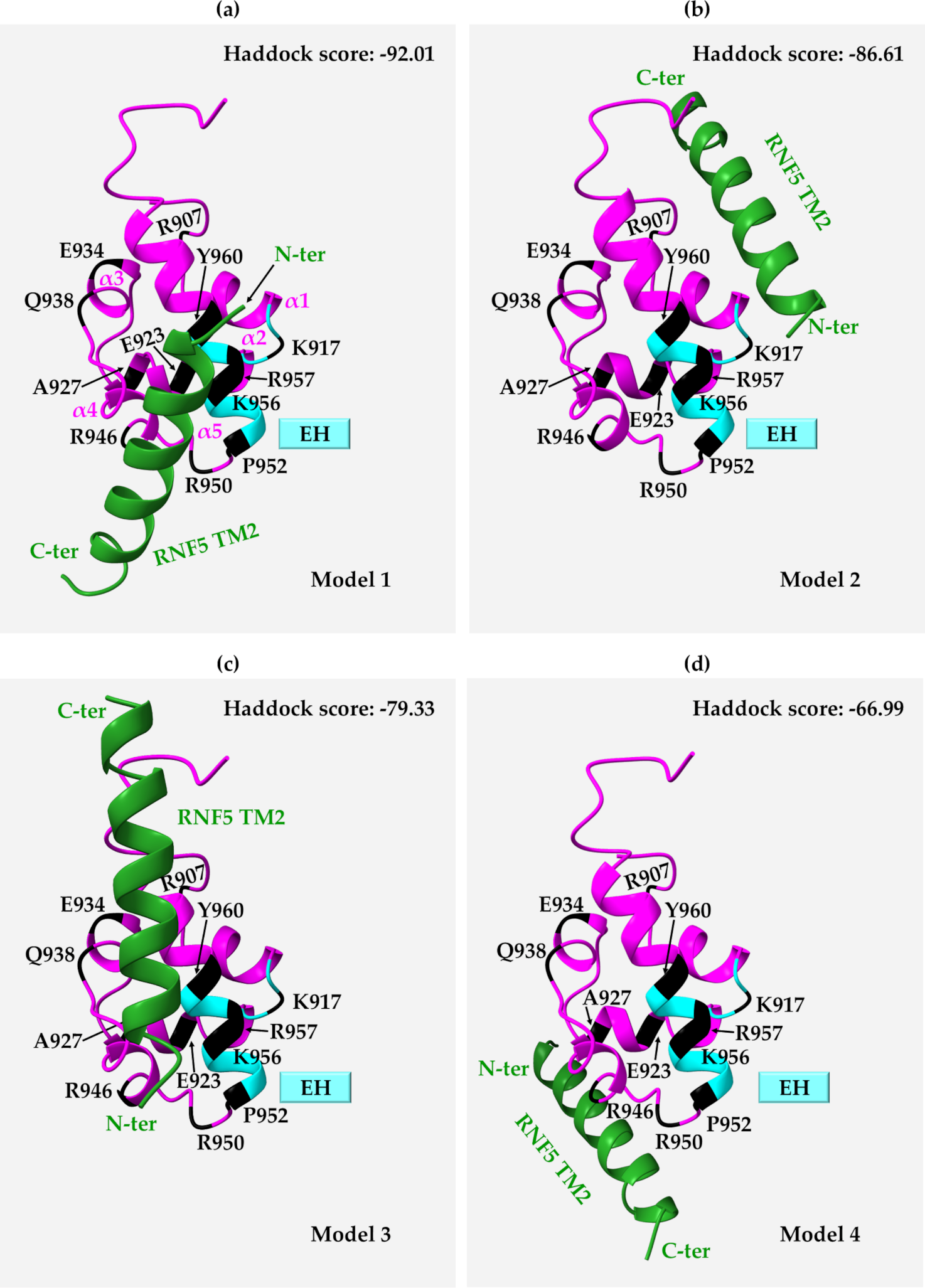

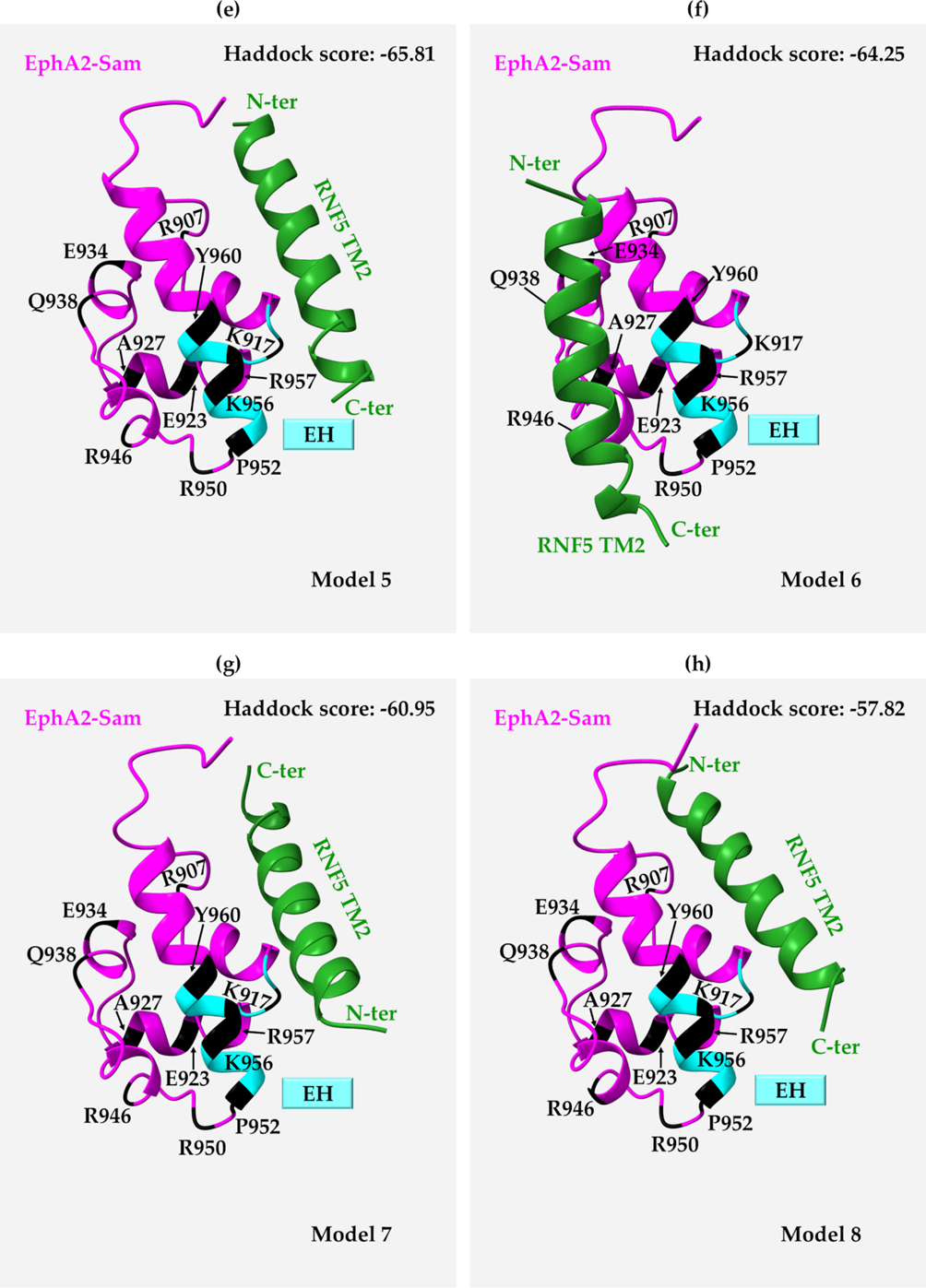
*

*
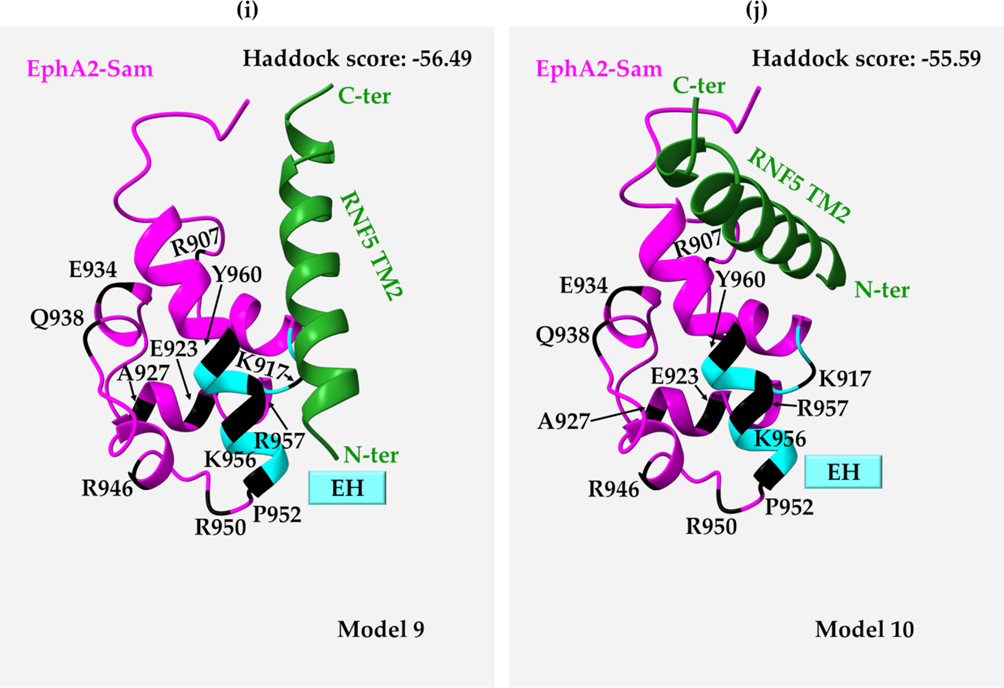
*

**Fig. S22.** Docking results for the EphA2-Sam/TM2 domain complex. (a-j) In each panel, a different docking solution among the best 10 -in terms of Haddock scores[54]- is shown. Each structure is reported in a ribbon representation: EphA2-Sam is colored magenta with a cyan EH region (residues I916-M918 and P952-Y960). The backbone of residues with solvent exposure higher than 40% and set as active during the docking calculations (i.e., R907, K917, E923, A927, E934, Q938, R946, R950, P952, K956, R957, Y960) is highlighted in black on the EphA2-Sam surface. The TM2 peptide is shown in a green ribbon drawing.

**
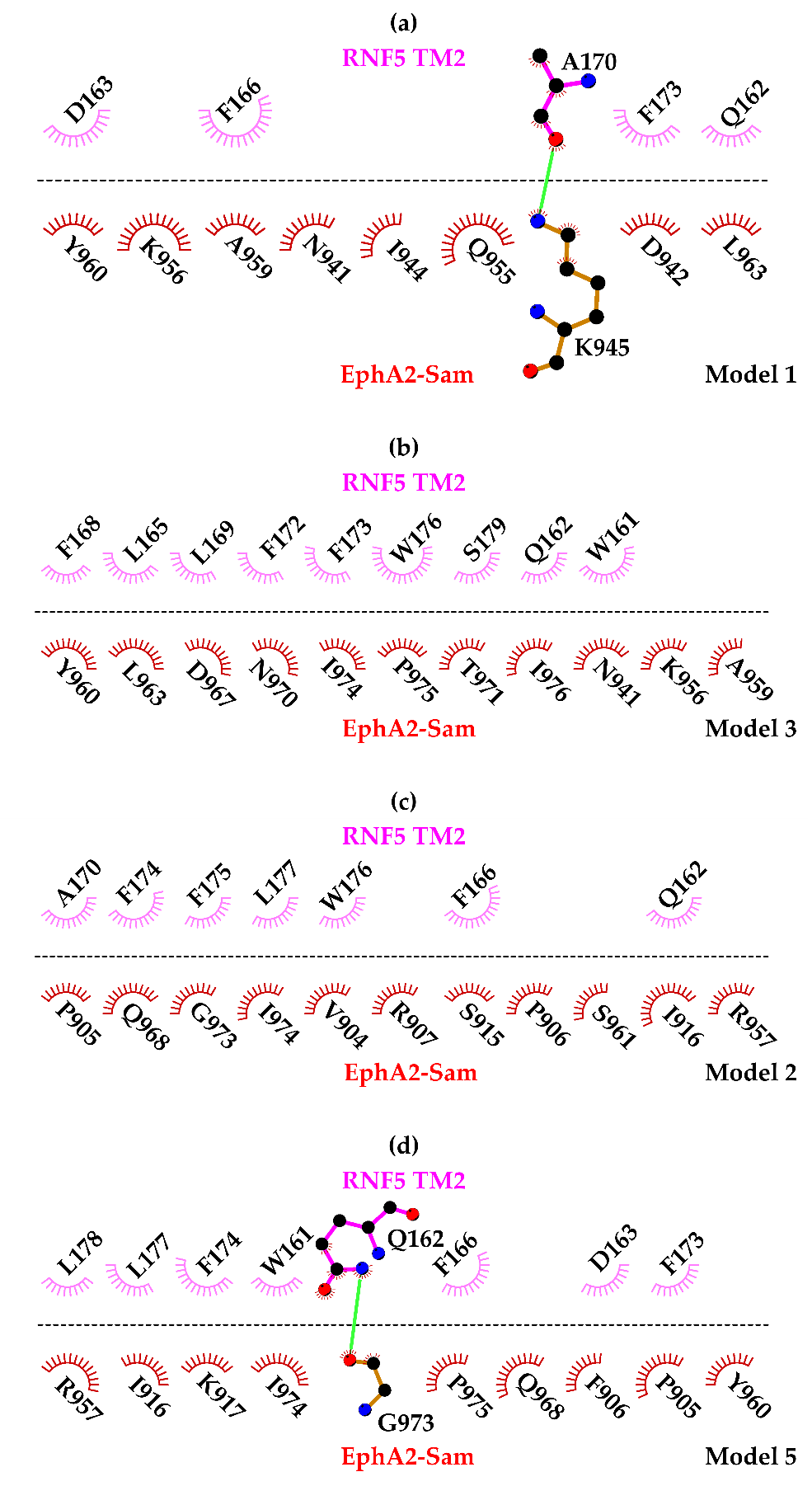
**

**Fig. S23.** 2D diagrams of intermolecular interactions generated by LigPlot+ [63,64] for the selected EphA2-Sam/RNF5 TM2 peptide docking poses. (a) 1^st^ Haddock pose. (b) 3^rd^ Haddock pose. (c) 2^nd^ Haddock pose. (d) 5^th^ Haddock pose. Residues involved in non-bonded interactions are labelled and represented by red and magenta crescents with bristles. Green lines indicate H-bonds.

**Table S6.** EphA2-Sam/RNF5 TM2 complex: intermolecular H-bonds and non-bonded interactions provided by EphA2-Sam. Intermolecular contacts refer to the selected Haddock [54] solutions among the 10 best ones (i.e., 1^st^ best model, 2^nd^ best model, 3^rd^ best model, 5^th^ best model, 6^th^ best model, 7^th^ best model, 8^th^ best model and 9^th^ best model) in terms of Haddock scores obtained through docking runs starting from the NMR structure of EphA2-Sam (1^st^ conformer of the NMR ensemble, PDB code 2E8N) and the best ranking AF2 model of the RNF5 TM2. The counting of the contacts was obtained starting from the lists of contacts generated by Haddock for the selected models. Underlined residues belong to the EphA2-Sam EH interface (I916-M918, P952-Y960).

| **EphA2-Sam Residue** | **Number of**  **H-bonds** | **Number of**  **non-bonded interactions** | **EphA2-Sam Residue** | **Number of**  **H-bonds** | **Number of**  **non-bonded interactions** |
| --- | --- | --- | --- | --- | --- |
| **P905** | 1 | 12 | **A959** | 0 | 4 |
| **F906** | 0 | 9 | **Y960** | 0 | 20 |
| **R907** | 1 | 0 | **S961** | 0 | 6 |
| **S915** | 0 | 1 | **L962** | 0 | 2 |
| **I916** | 0 | 6 | **L963** | 0 | 8 |
| **K917** | 0 | 1 | **G964** | 0 | 5 |
| **T940** | 0 | 1 | **K966** | 0 | 3 |
| **N941** | 1 | 10 | **D967** | 0 | 2 |
| **D942** | 0 | 4 | **Q968** | 0 | 3 |
| **I944** | 0 | 4 | **N970** | 0 | 7 |
| **K945** | 3 | 4 | **T971** | 0 | 3 |
| **G953** | 0 | 1 | **G973** | 1 | 2 |
| **Q955** | 0 | 3 | **I974** | 0 | 5 |
| **K956** | 0 | 12 | **P975** | 0 | 4 |
| **R957** | 3 | 4 | **I976** | 0 | 2 |

**Fig. S24.** Far-UV circular dichroism spectrum of RNF5-PEP2 (100 µM peptide concentration) recorded in 10 mM phosphate buffer (pH 7.4).

**
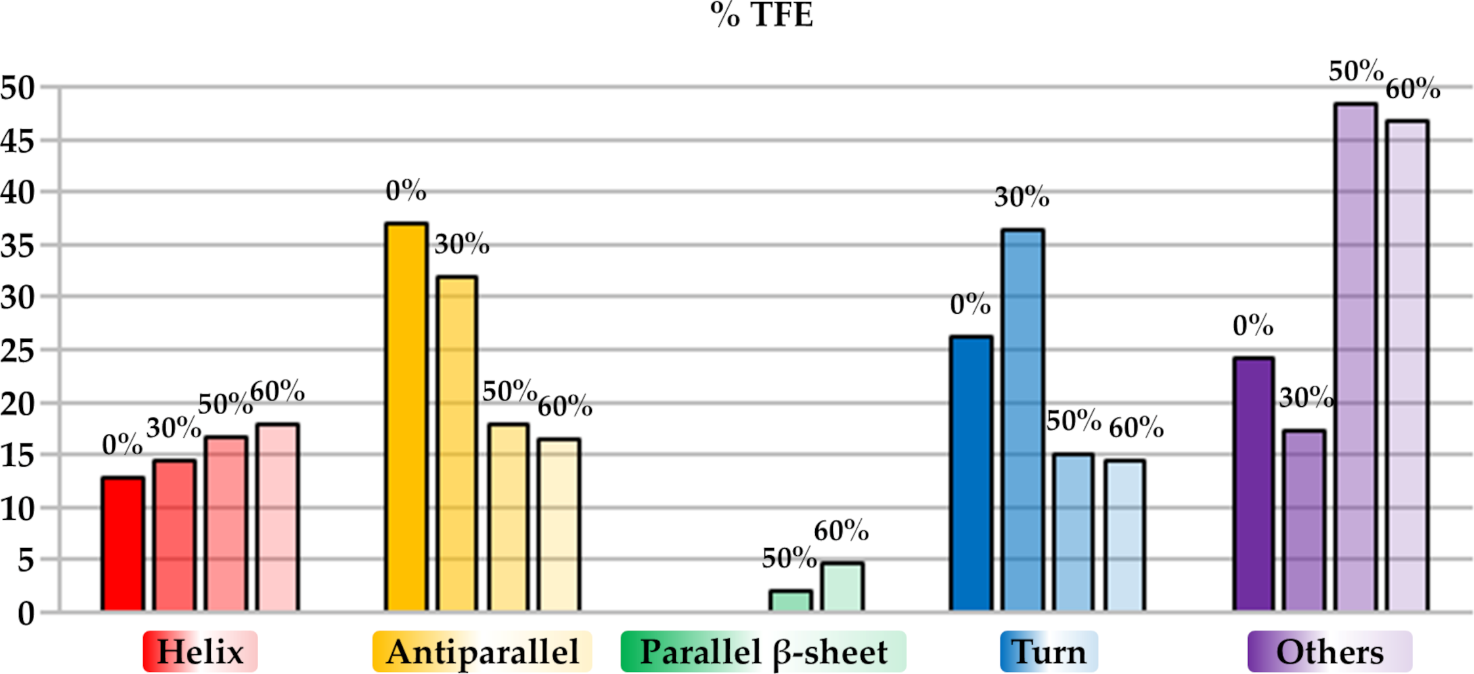
**

**Fig. S25.** Deconvolution of CD spectra of RNF5-PEP3 at increasing TFE amounts (i.e., 0%, 30%, 50% and 60%). Red, orange, green, blue, and violet were used for content in Helix, Antiparallel, Parallel β-sheet, Turn and Others, respectively. The percentage of TFE was reported above each bar as well. The deconvolutions of CD spectra were obtained by BeStSel software (<http://bestsel.elte.hu/> access date 29/12/2025).

**Table S7.** CD spectral analyses: secondary-structure deconvolution of RNF5-PEP3 in absence and presence of TFE obtained using the BeStSel [66] algorithm over the 190-250 nm wavelength range.

| **Secondary structure category** | **0% TFE** | **30% TFE** | **50% TFE** | **60% TFE** |
| --- | --- | --- | --- | --- |
| Helix (total) | 12.7 | 14.5 | 16.6 | 17.8 |
| Antiparallel (total) | 37.0 | 32.0 | 17.8 | 16.4 |
| Parallel β-sheet | 0.0 | 0.0 | 2.1 | 4.7 |
| Turn | 26.3 | 36.4 | 15.0 | 14.5 |
| Others | 24.1 | 17.2 | 48.5 | 46.7 |


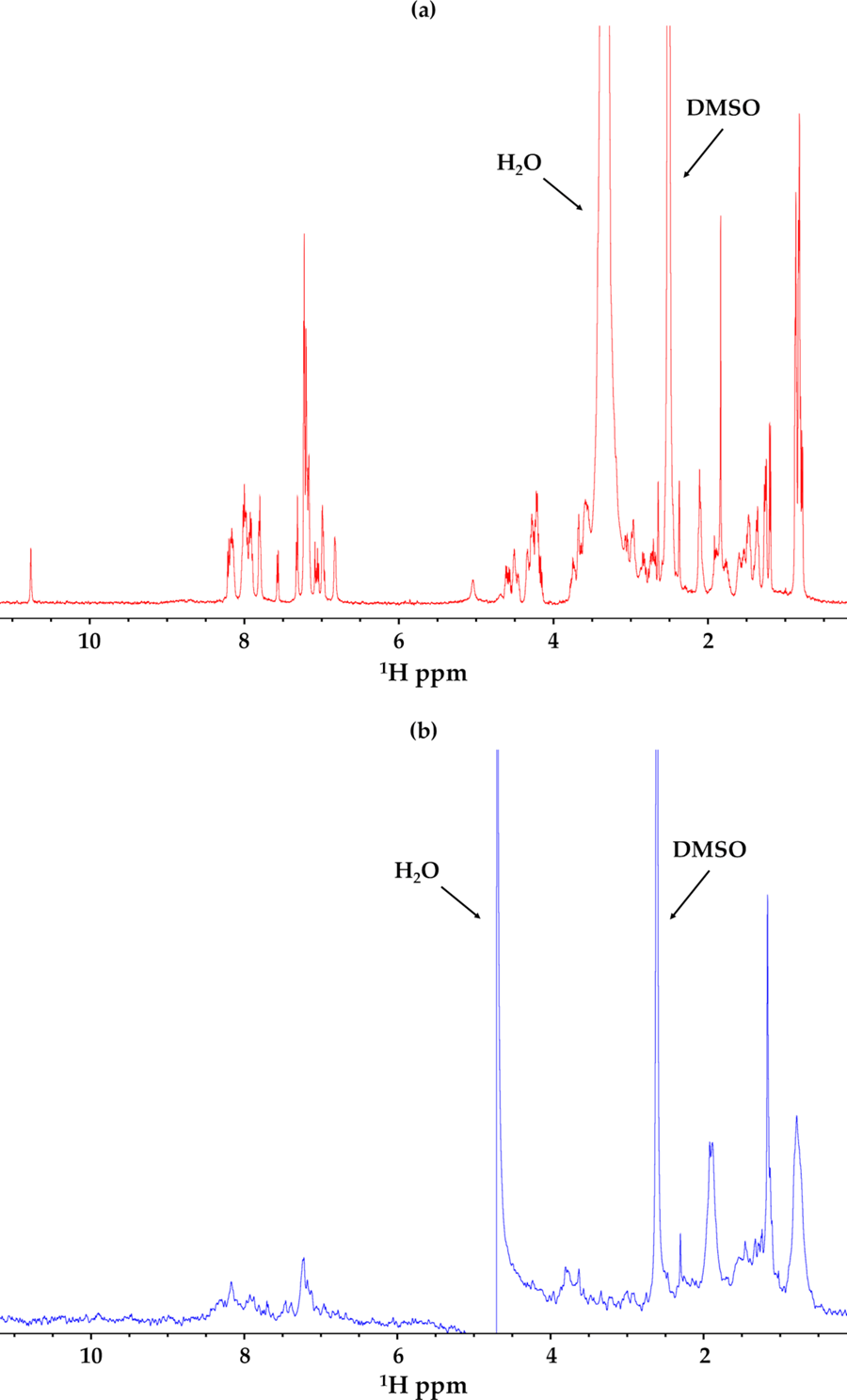


**Fig. S26.** 1D [^1^H] NMR spectra of RNF5-PEP3 458 µM in 100% DMSO-d6 (a) and RNF5-PEP3 330 µM concentration in PBS pH 7.4/D_2_O 90/10 v/v (b). All NMR samples were prepared starting from a stock solution made up of the peptide (55 mM concentration) dissolved in DMSO-d6.

**
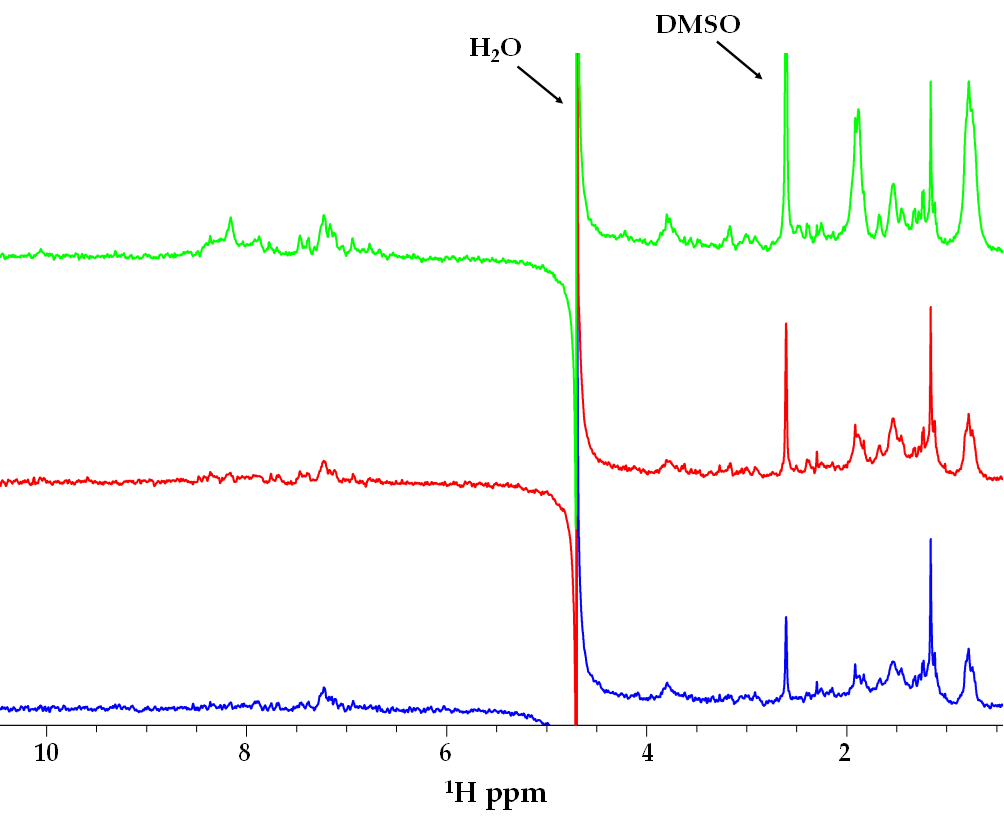
**

**Fig. S27.** 1D [^1^H] NMR spectra of RNF5-PEP3 registered in PBS pH 7.4/D_2_O 90/10 v/v at decreasing concentrations arising from the sample serial dilution: 330 µM (green), 100 µM (red) and 50 µM (blue). A peptide stock solution (55 mM concentration) in DMSO-d6 was used for NMR sample preparation.

**
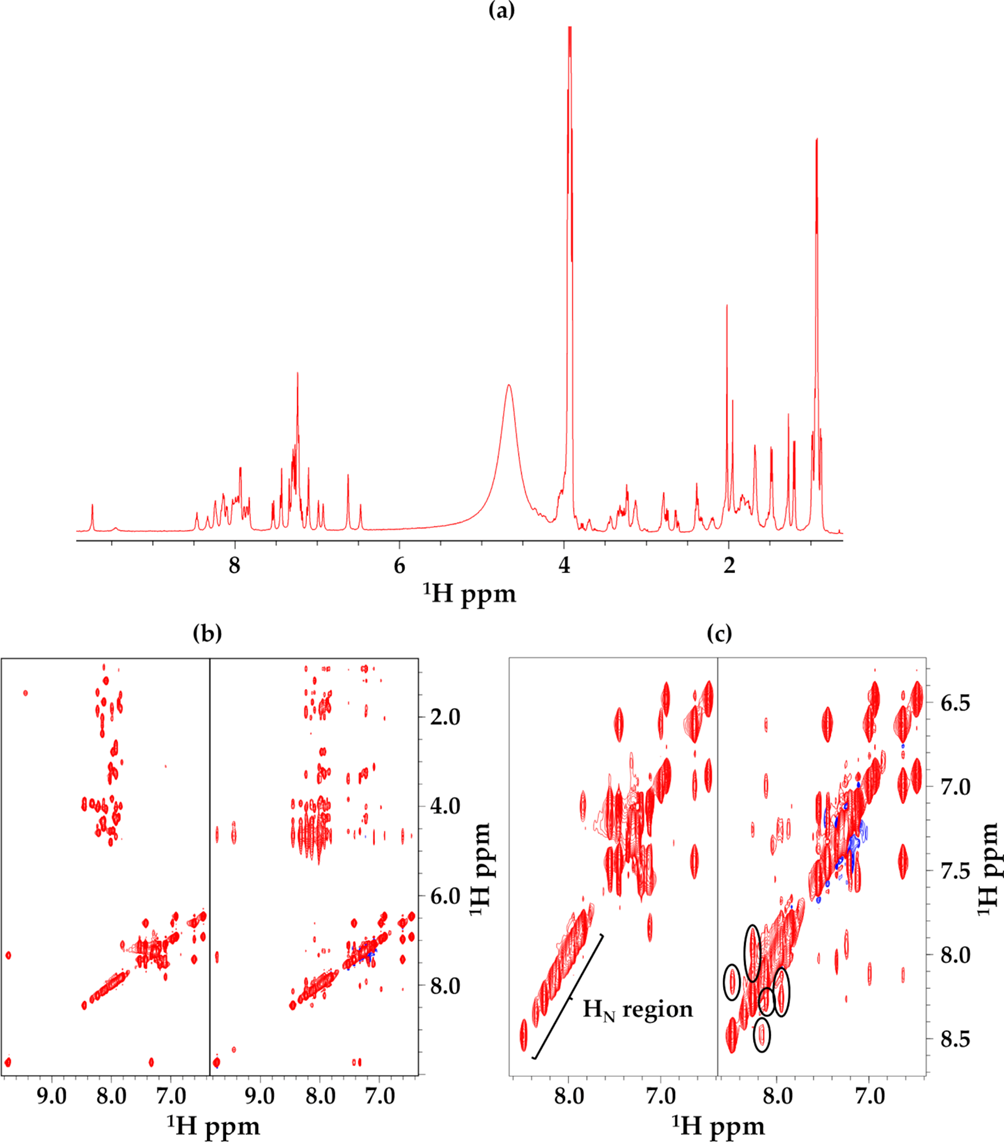
**

**Fig. S28.** NMR spectra acquired for RNF5-PEP3 in PBS/TFE 40/60 v/v (peptide concentration equal to 450 µM; pH=6.8). (a) 1D [^1^H] NMR spectrum, (b, c) 2D [^1^H, ^1^H] TOCSY (left panels) and NOESY (right panels); the H_N_-aromatic/aliphatic regions of TOCSY and NOESY spectra are reported in (b), while a detail of only the H_N_-aromatic regions is shown in (c). A few sequential H_N_i-H_N_i+1 correlations in the NOESY spectrum (c, right panel) are highlighted within circles.


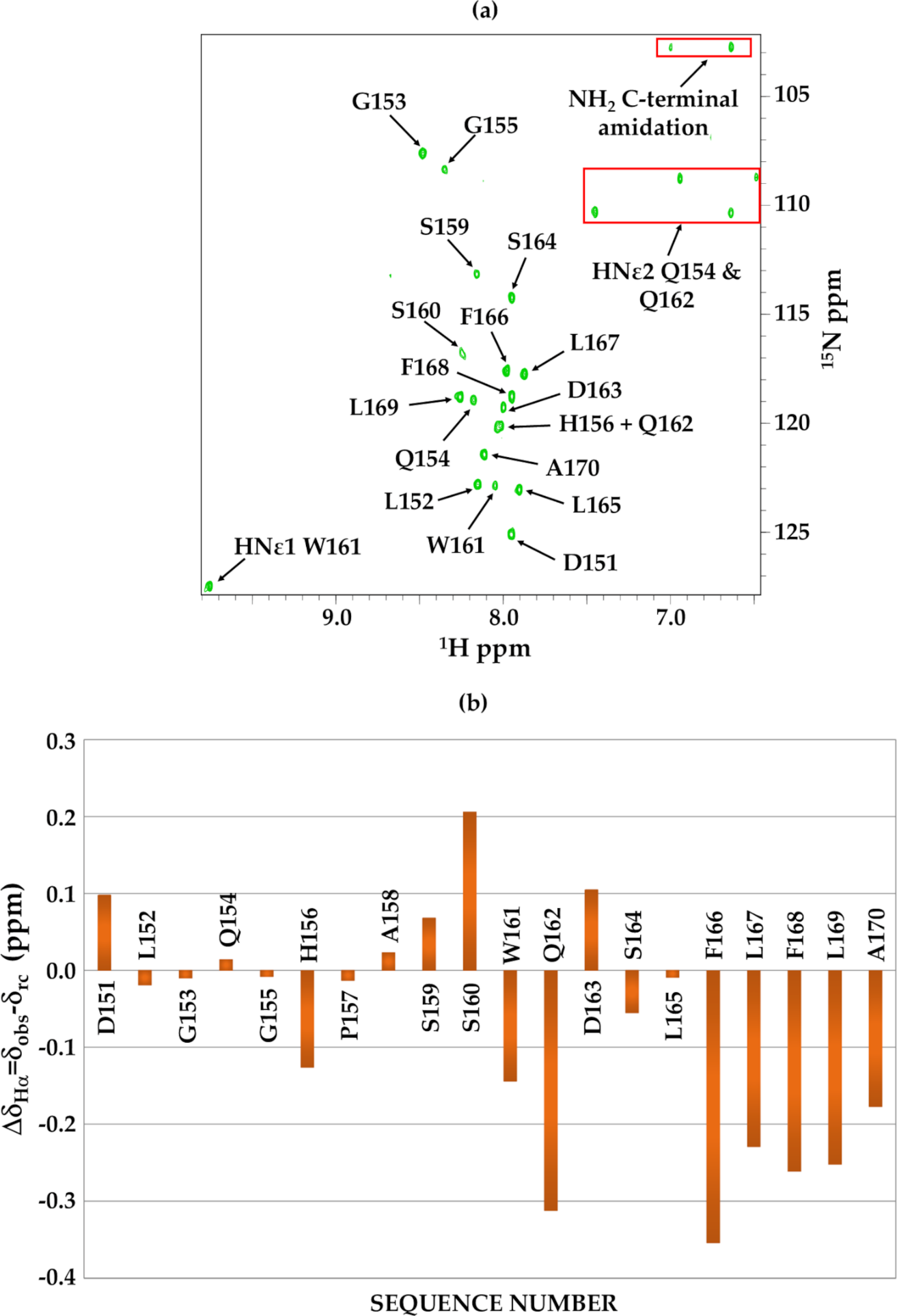


**Fig. S29.** (a) 2D [^1^H, ^15^N] HSQC NMR spectrum registered for RNF5-PEP3 in PBS/TFE 40/60 v/v with backbone and side chains HN assignments indicated by black arrows. (b) Graph of Hα proton chemical shift deviations (Δδ_Hα_=δ_obs_-δ_rc_ where δobs and δrc refer to the measured Hα chemical shifts and the predicted random coil values, respectively) calculated for RNF5-PEP3 in PBS/TFE 40/60 v/v (T=298 K, pH=6.8).


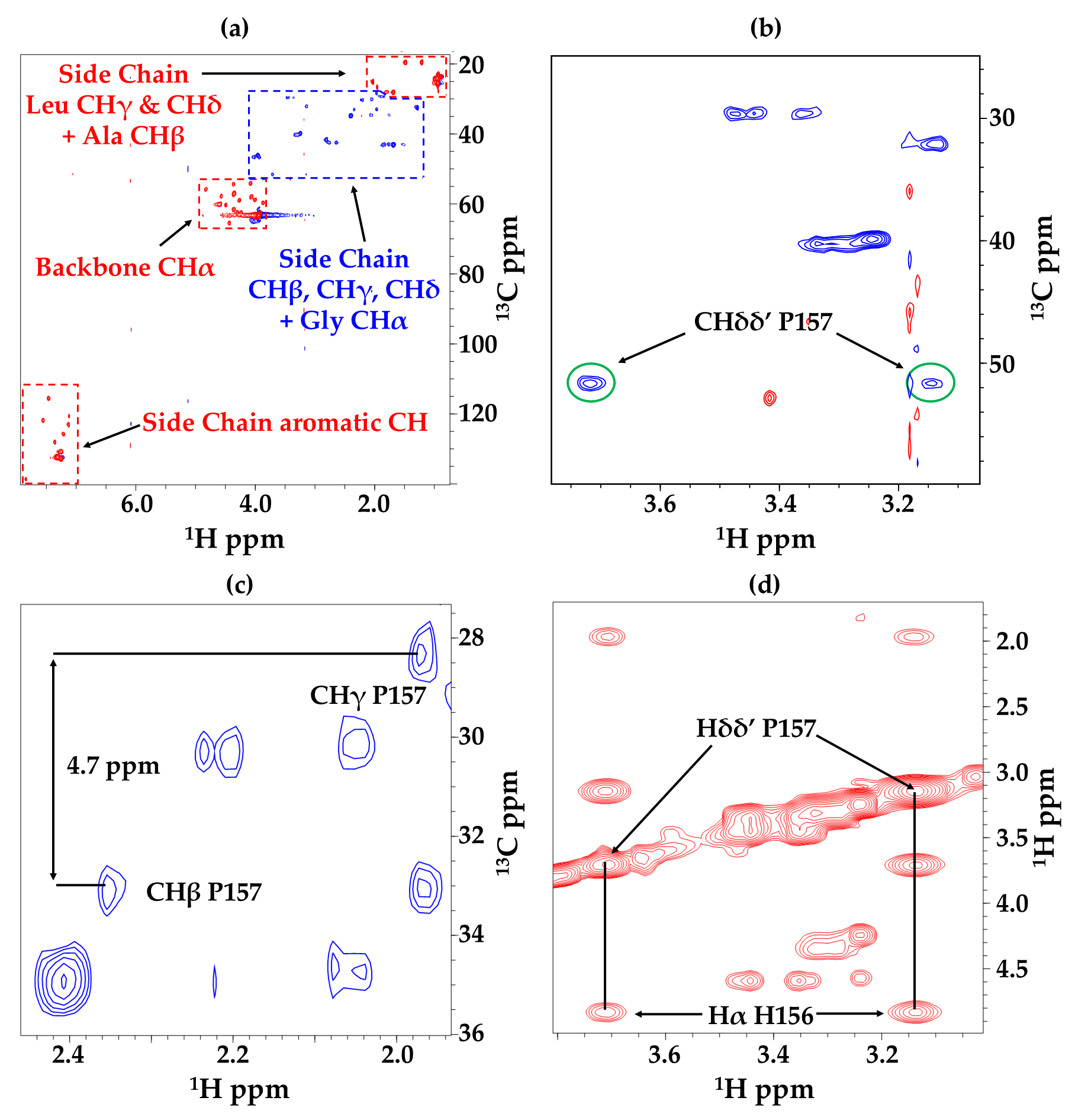


**Fig. S30.** (a) [^1^H, ^13^C] HSQC spectrum [140] of RNF5-PEP3 in PBS/TFE 40/60 v/v: different spectral regions including ^13^CH,^13^CH_3_ and ^13^CH_2_ peptide peaks have been highlighted. (b,c) Expansions of two sections from (a) showing the main P157 correlations (i.e., ^13^CHδ/δ’ (b) and ^13^CHβ, ^13^CHγ (c)). In (c) the chemical shift difference between P157 ^13^C_H_β and ^13^C_H_γ resonances, which is diagnostic of a Proline in trans configuration, is reported [126]. (d) A detail of the NOESY spectrum showing the Hδδ’_i+1_ - Hα_i_ NOEs canonical of a Proline in trans configuration [74].

**
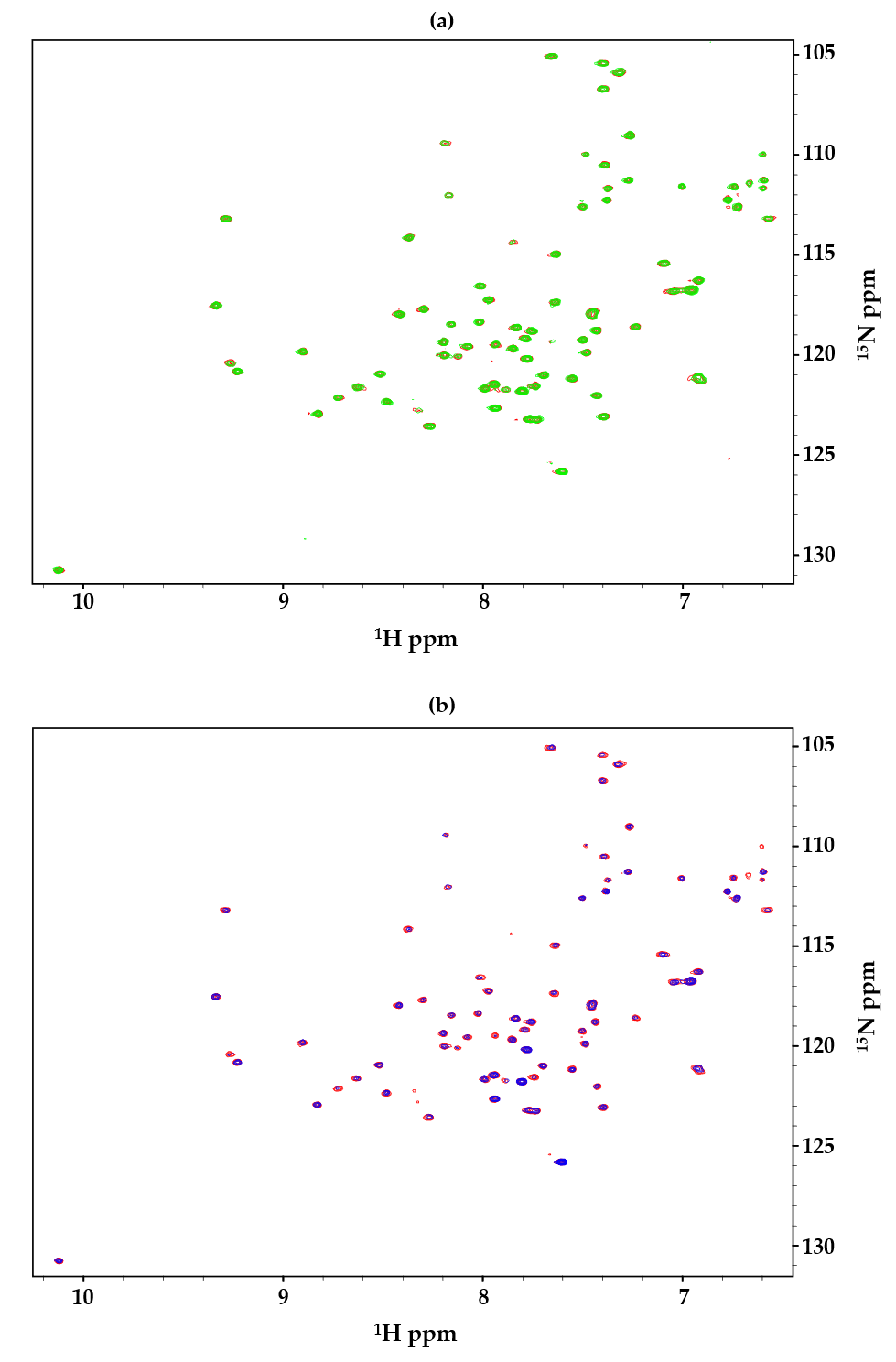
**

**
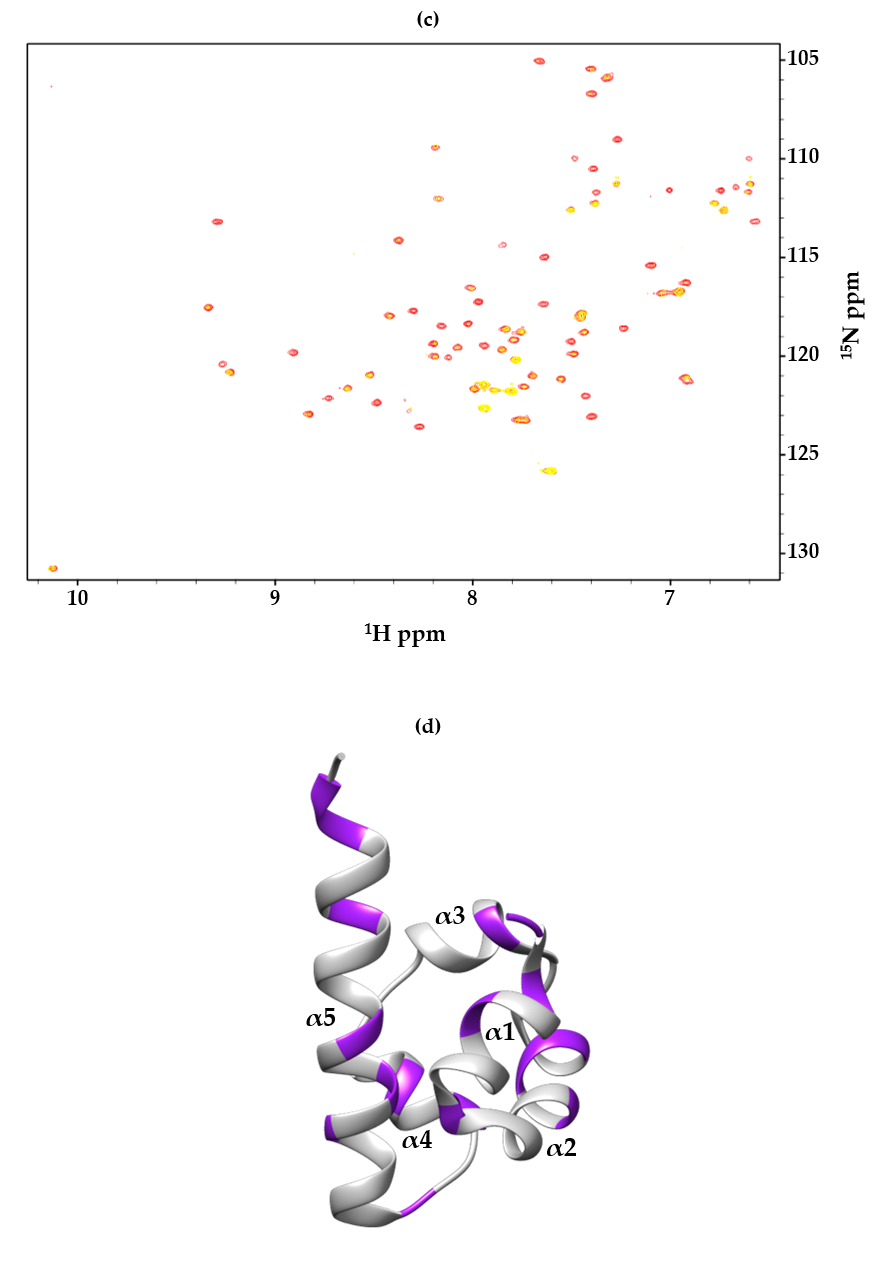
**

**Fig. S31.** Superposition of [^1^H, ^15^N] HSQC spectra recorded for ^15^N labelled EphA2-Sam alone (14.8 µM concentration) (red in all panels) and after addition of RNF5-PEP3 at increasing concentrations: (a) 45 µM (green); (b) 160 µM (blue); (c) 250 µM (yellow). (d) The first conformer of the EphA2-Sam NMR structure (PDB code 2E8N) is shown in ribbon representation; protein residues most affected by peak intensity decrease after addition of 250 µM of RNF5-PEP3 are coloured violet on the structure.


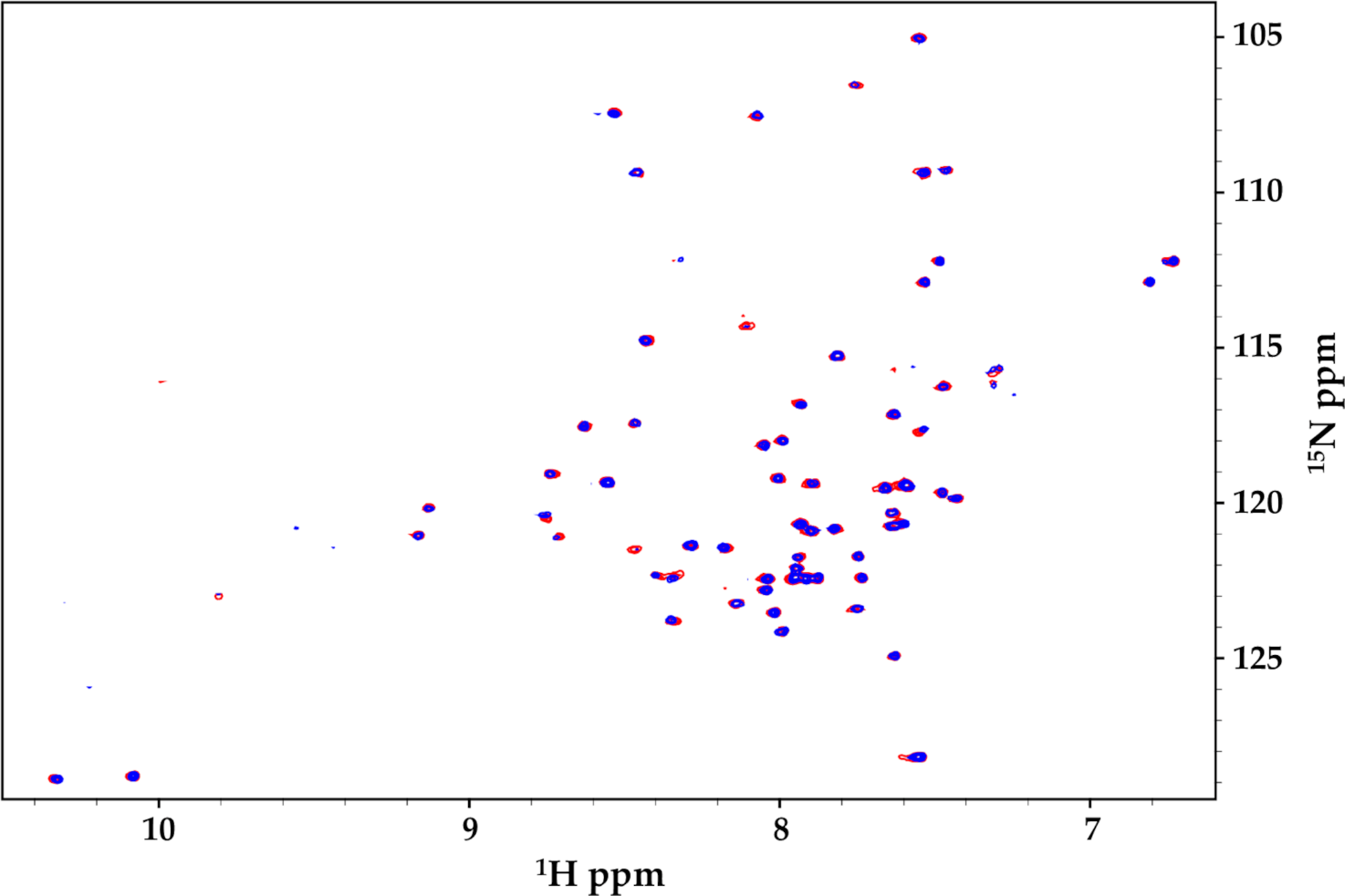


**Fig. S32.** Superposition of [^1^H, ^15^N] HSQC spectra obtained for Ship2-Sam at a concentration equal to 14.8 µM in the absence (red) and in the presence (blue) of RNF5-PEP3 (330 µM concentration).

**
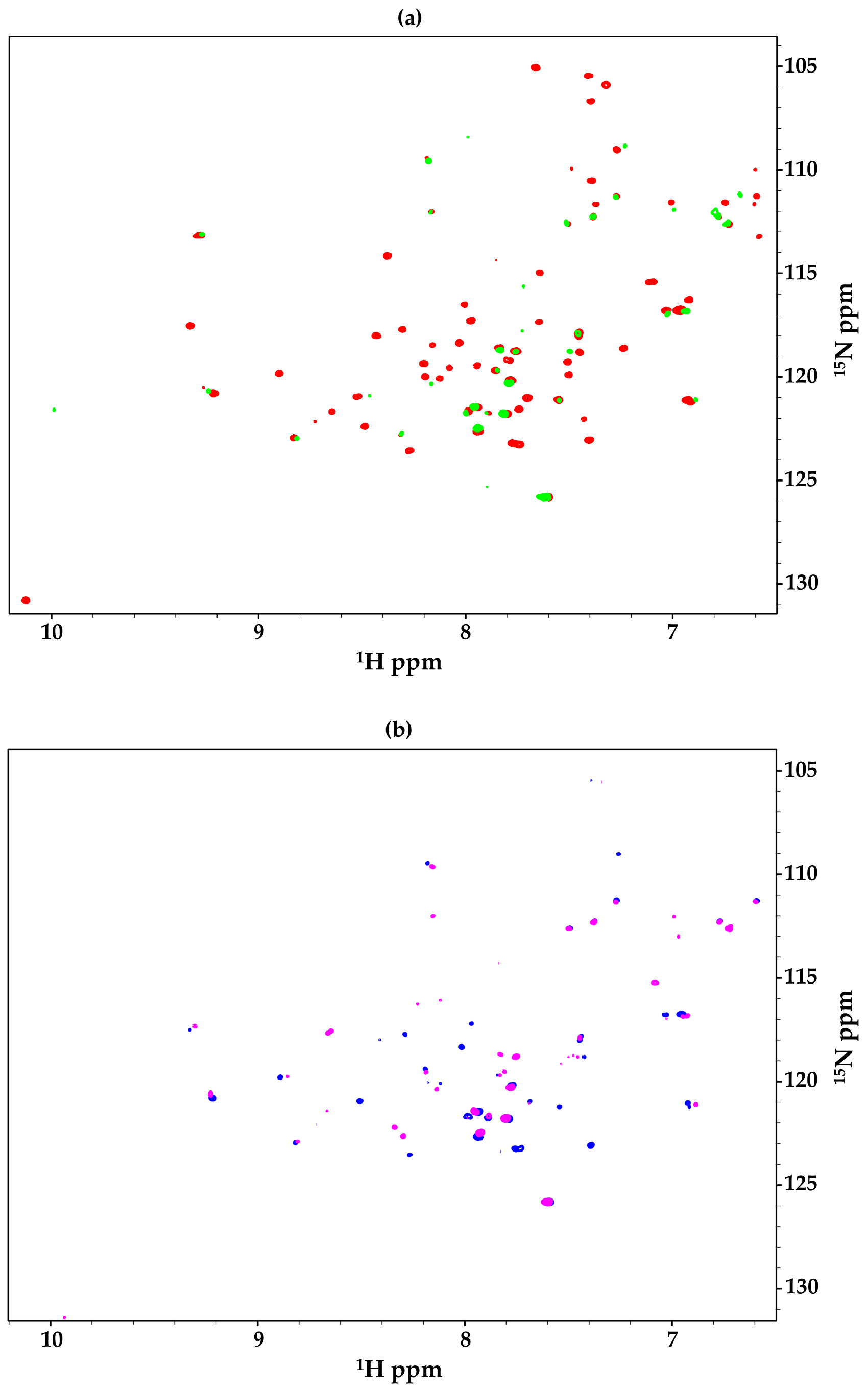
**

**
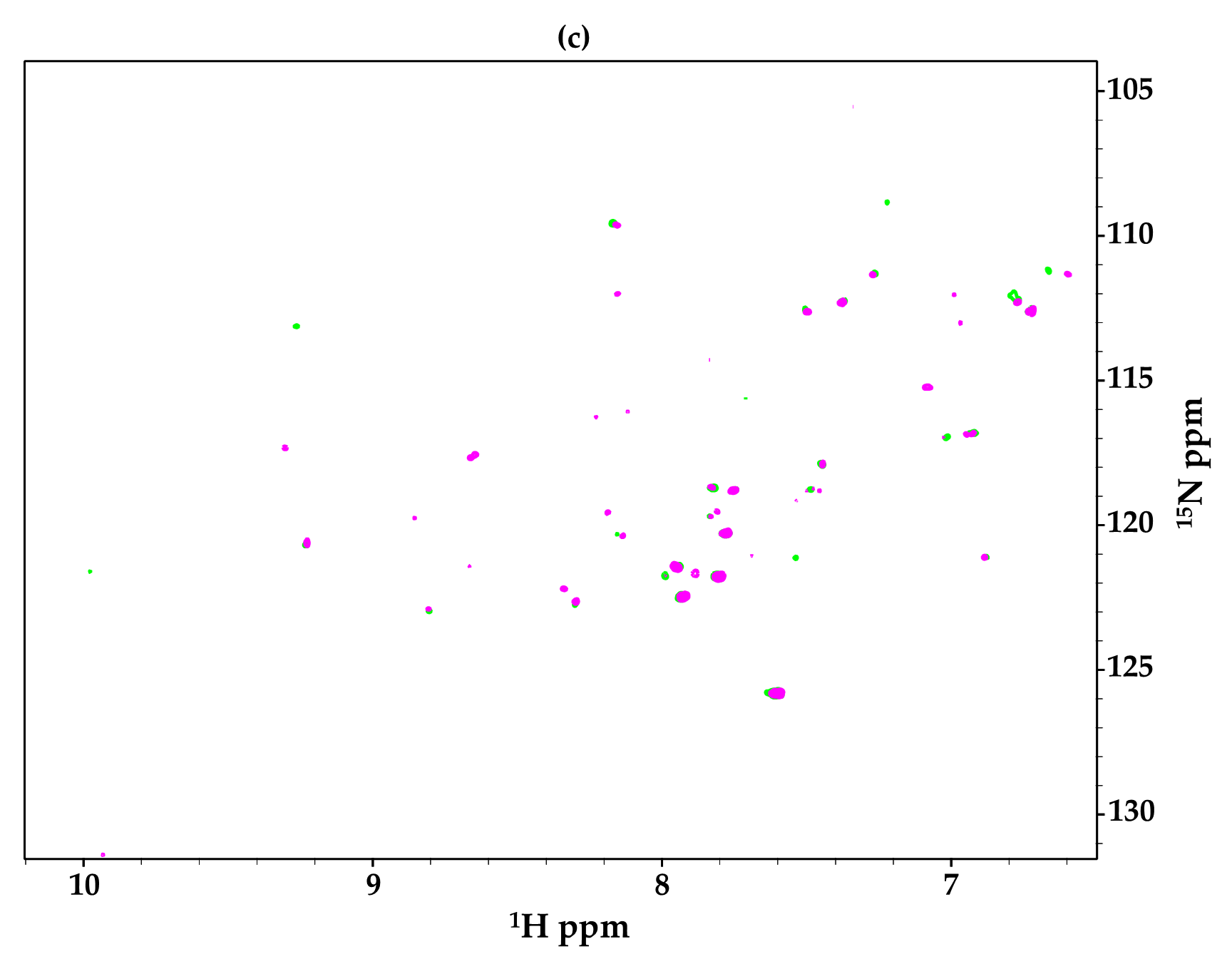
**

**Fig. S33.** Superposition of [^1^H, ^15^N] HSQC spectra recorded for (a) ^15^N labelled EphA2-Sam alone (14.8 µM concentration) (red) and after addition of unlabelled Ship2-Sam (45 µM concentration) to the sample containing ^15^N labelled EphA2-Sam and RNF5-PEP3 (330 µM concentration) (green); (b) ^15^N labelled EphA2-Sam in presence of RNF5-PEP3 before (blue) and after (magenta) addition of unlabelled Ship2-Sam (108 µM concentration); (c) ^15^N labelled EphA2-Sam in presence of both RNF5-PEP3 and unlabelled Ship2-Sam at 45 µM concentration (green) or 108 µM concentration (magenta). No relevant changes in the HSQC spectra could be detected by increasing the EphA2-Sam/Ship2-Sam concentration ratio from 1:3 to 1:7. Thus, a much higher Ship2-Sam amount is required to move RNF5-PEP3 away and to allow formation of the Ship2-Sam/EphA2-Sam complex.

**
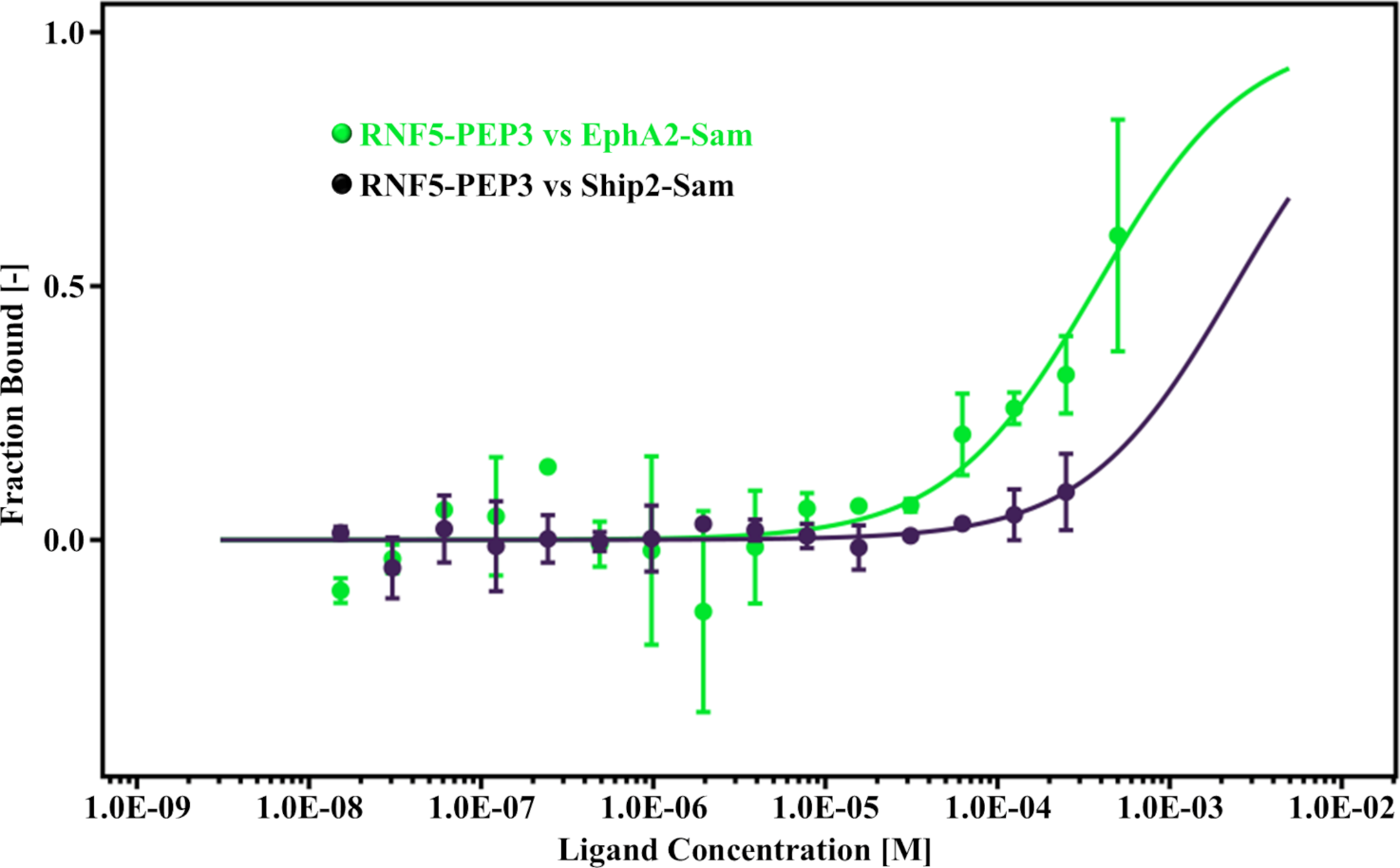
**

**Fig. S34.** MST interaction analysis. The graph shows dose-response curves of RNF5-PEP3 titrated against EphA2-Sam (green curve) and Ship2-Sam (violet curve).

**Table S8.** Table of H_N_ and Hα chemical shifts of RNF5-PEP3 obtained from NMR spectra registered with the peptide 450 µM concentrated in PBS/TFE 40/60 v/v, 298 K and pH=6.8.

| **Residue** | **H_N_** | **Hα** |
| --- | --- | --- |
| D151 | 7.95 | 4.61 |
| L152 | 8.15 | 4.36 |
| G153 | 8.48 | 4.01-3.93 |
| Q154 | 8.17 | 4.35 |
| G155 | 8.35 | 3.97 |
| H156 | 8.04 | 4.96 |
| P157 |  | 4.45 |
| A158 | 9.46 | 4.34 |
| S159 | 8.16 | 4.42 |
| S160 | 8.24 | 4.41 |
| W161 | 8.05 | 4.73 |
| Q162 | 8.02 | 4.18 |
| D163 | 8.00 | 4.46 |
| S164 | 7.95 | 4.38 |
| L165 | 7.90 | 4.29 |
| F166 | 7.98 | 4.61 |
| L167 | 7.87 | 4.24 |
| F168 | 7.94 | 4.62 |
| L169 | 8.25 | 4.31 |
| A170 | 8.11 | 4.26 |


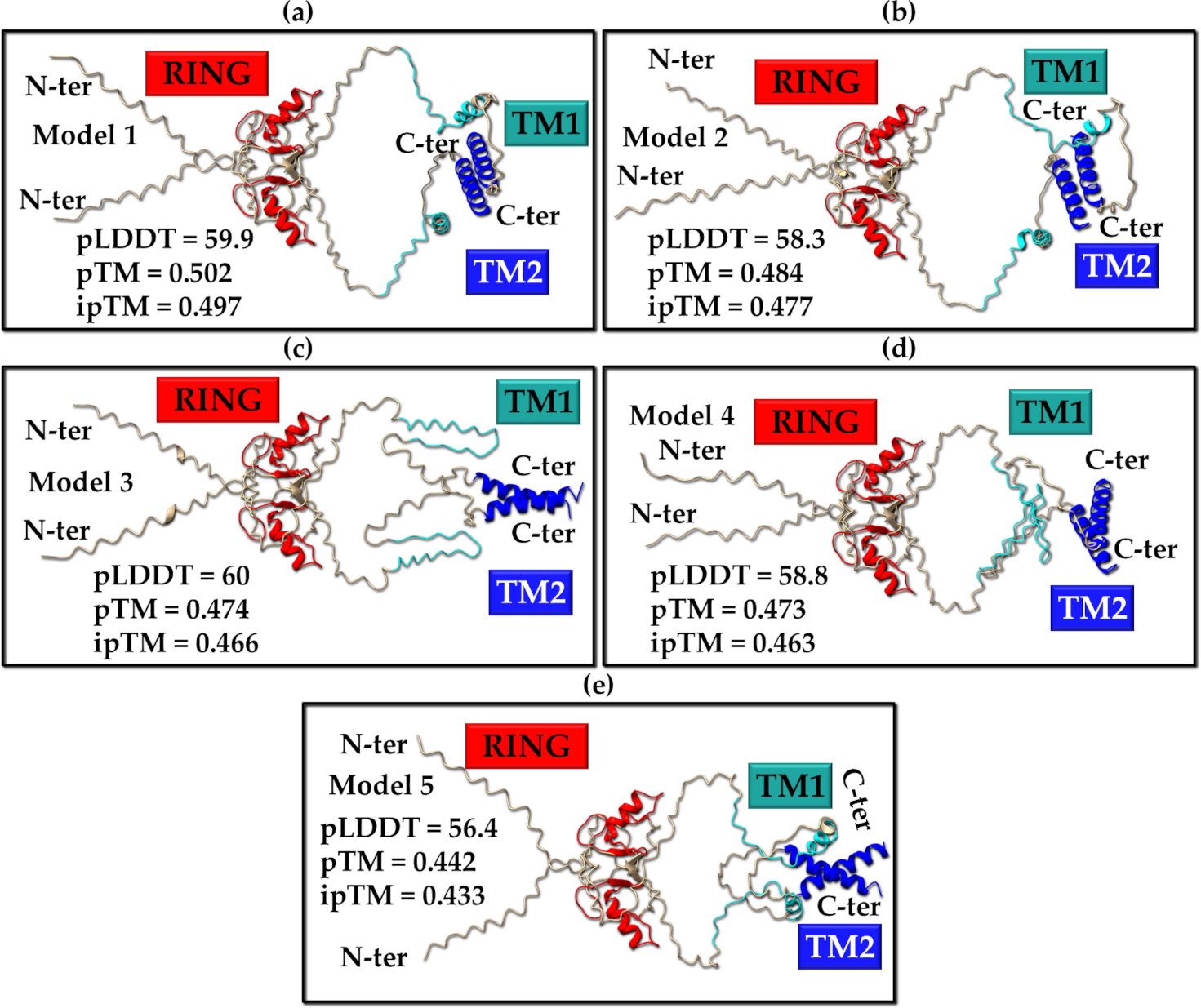


**Fig. S35.** (a-e) AF2 [49,55] structure models (from the 1^st^ to the 5^th^ ranked structures) of the RNF5 homodimer (residues M1-I180 from UniProt [57] entry Q99942 for human RNF5). The RING domain (residue range C27-K68) is shown in red; the first transmembrane segment (TM1) (residues G118-F138) in light sea green, and the second transmembrane segment (TM2) (residues S160-I180) in blue. The confidence pLDDT, pTM and ipTM scores of each model are indicated in the corresponding panel [107,137-139].

**
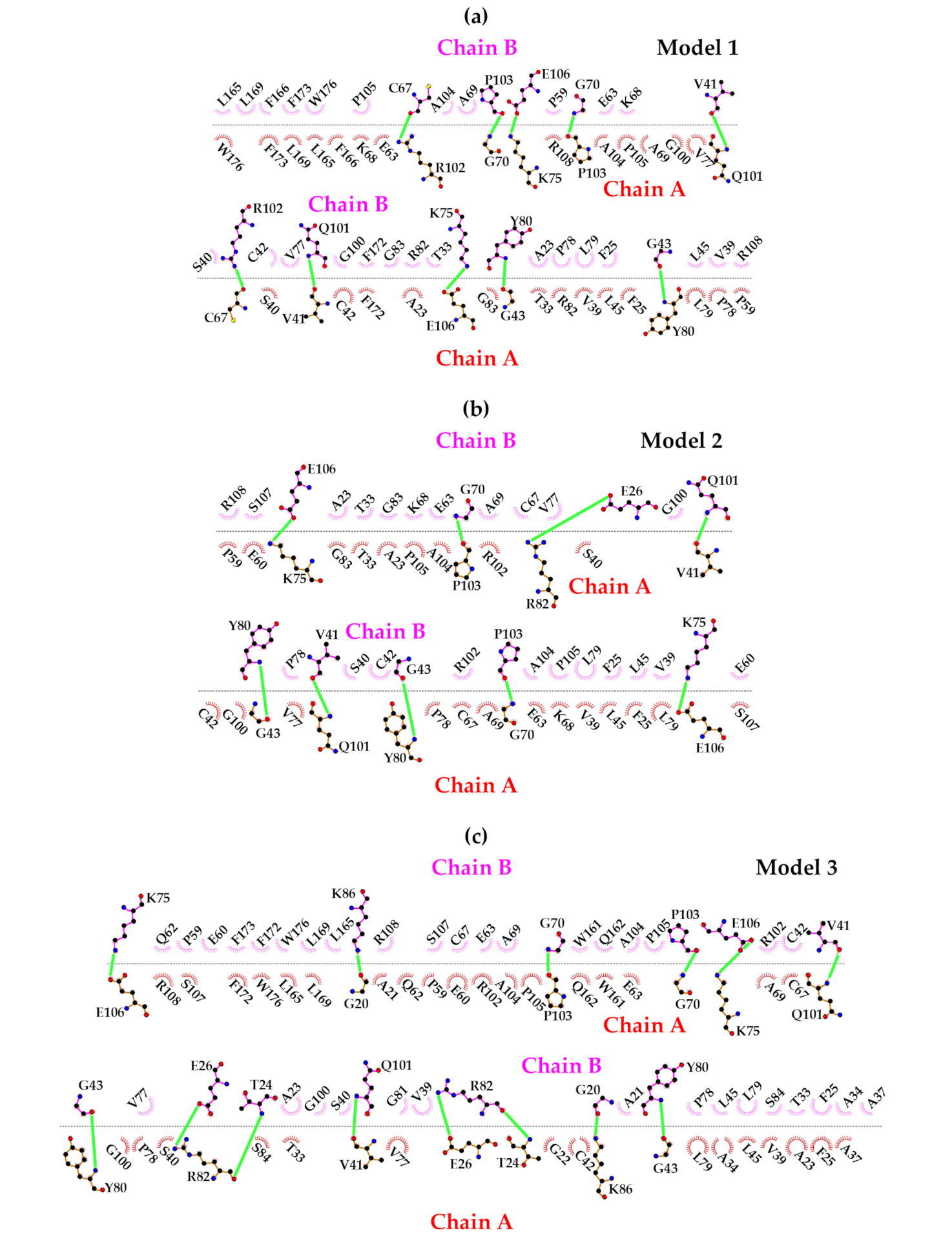
**

**
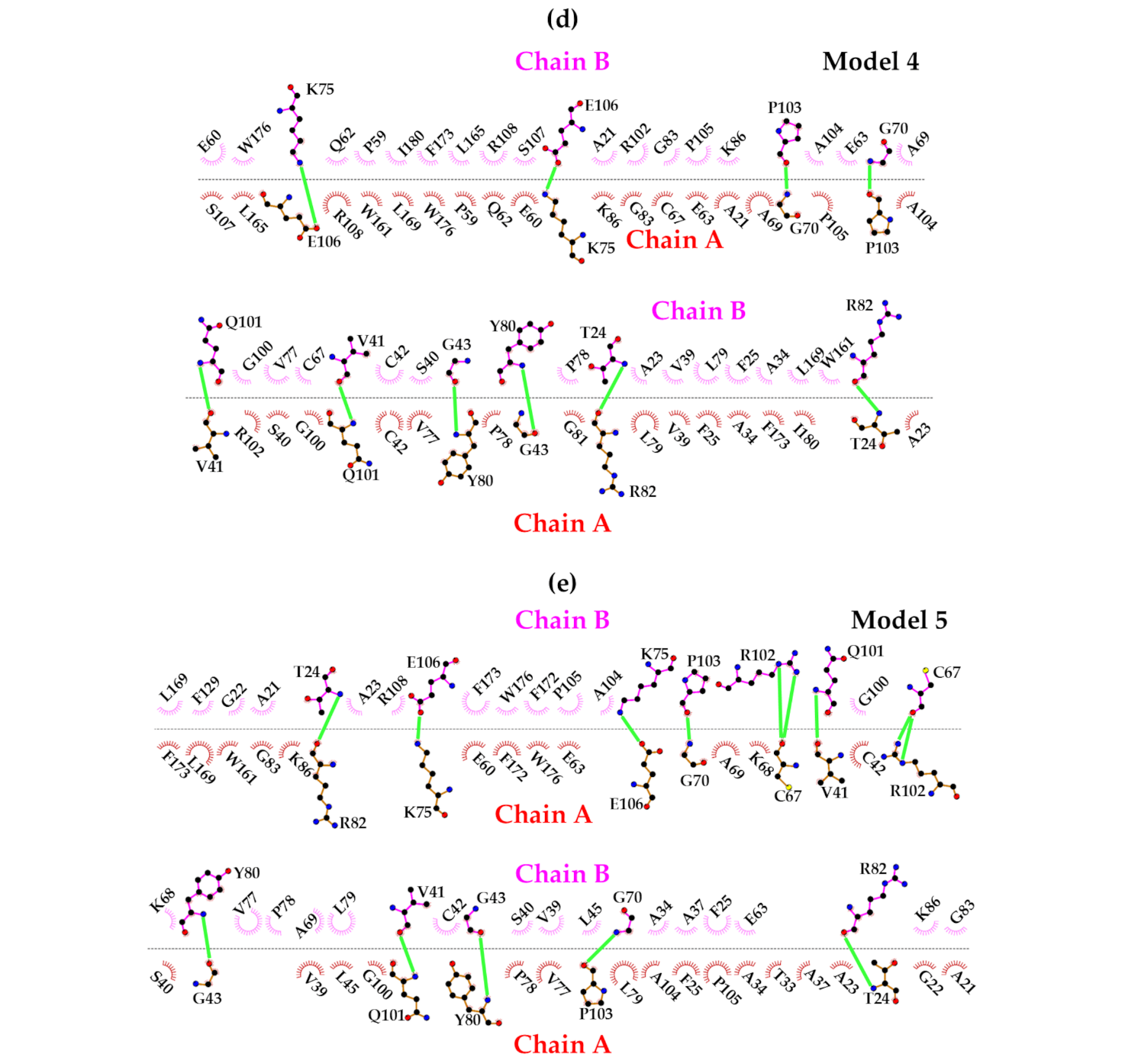
**

**Fig. S36.** 2D diagrams of intermolecular interactions generated by LigPlot+ [63,64] for the AF2 [49,55] models of the RNF5 homodimer (residues M1-I180 from UniProt [57] entry Q99942 for human RNF5). (a) 1^st^ ranked, (b) 2^nd^ ranked, (c) 3^rd^ ranked, (d) 4^th^ ranked and (e) 5^th^ ranked model. Residues involved in non-bonded interactions are labelled with the one letter amino acid code and sequence numbers and represented by red (if positioned inside Chain A) or magenta (if belonging to Chain B) crescents with bristles. H-bonds are represented by solid green lines.

**Table S9.** Interchain H-bonds and non-bonded interactions of the five AF2 [49,55] homodimeric models of the RNF5 protein (residues M1-I180 from UniProt [57] entry Q99942 for human RNF5). The global number of intermolecular interactions per residue (i.e., sum of all intermolecular contacts within the 5 AF2 models) was derived from the list of contacts generated by LigPlot+ [63,64].

| **RNF5 residues** | **Number of H-bonds** | | **Number of non-bonded interactions** | |
| --- | --- | --- | --- | --- |
|  | **Chain A** | **Chain B** | **Chain A** | **Chain B** |
| G20 | 1 | 1 | 2 | 3 |
| A21 | 0 | 0 | 22 | 23 |
| G22 | 0 | 0 | 3 | 1 |
| A23 | 0 | 0 | 19 | 19 |
| T24 | 3 | 3 | 15 | 16 |
| F25 | 0 | 0 | 96 | 99 |
| E26 | 1 | 2 | 1 | 5 |
| T33 | 0 | 0 | 6 | 5 |
| A34 | 0 | 0 | 4 | 4 |
| A37 | 0 | 0 | 3 | 3 |
| V39 | 0 | 0 | 8 | 8 |
| S40 | 0 | 0 | 5 | 5 |
| V41 | 5 | 5 | 48 | 48 |
| C42 | 0 | 0 | 25 | 25 |
| G43 | 4 | 5 | 58 | 60 |
| L45 | 0 | 0 | 5 | 4 |
| P59 | 0 | 0 | 7 | 5 |
| E60 | 0 | 0 | 11 | 8 |
| Q62 | 0 | 0 | 6 | 6 |
| E63 | 0 | 0 | 14 | 14 |
| C67 | 3 | 3 | 12 | 12 |
| K68 | 0 | 0 | 5 | 5 |
| A69 | 0 | 0 | 24 | 23 |
| G70 | 5 | 5 | 47 | 46 |
| K75 | 5 | 5 | 12 | 11 |
| V77 | 0 | 0 | 15 | 14 |
| P78 | 0 | 0 | 16 | 16 |
| L79 | 0 | 0 | 42 | 42 |
| Y80 | 5 | 5 | 44 | 42 |
| G81 | 0 | 0 | 1 | 1 |
| R82 | 5 | 4 | 26 | 23 |
| G83 | 0 | 0 | 8 | 7 |
| S84 | 0 | 0 | 1 | 1 |
| K86 | 1 | 1 | 24 | 23 |
| G100 | 0 | 0 | 10 | 10 |
| Q101 | 5 | 5 | 41 | 42 |
| R102 | 3 | 3 | 24 | 26 |
| P103 | 5 | 5 | 35 | 34 |
| A104 | 0 | 0 | 16 | 16 |
| P105 | 0 | 0 | 31 | 31 |
| E106 | 5 | 5 | 13 | 15 |
| S107 | 0 | 0 | 3 | 3 |
| R108 | 0 | 0 | 13 | 17 |
| F129 | 0 | 0 | 0 | 3 |
| W161 | 0 | 0 | 15 | 9 |
| Q162 | 0 | 0 | 6 | 7 |
| L165 | 0 | 0 | 14 | 15 |
| F166 | 0 | 0 | 2 | 2 |
| L169 | 0 | 0 | 29 | 25 |
| F172 | 0 | 0 | 67 | 64 |
| F173 | 0 | 0 | 11 | 18 |
| W176 | 0 | 0 | 43 | 42 |
| I180 | 0 | 0 | 4 | 6 |


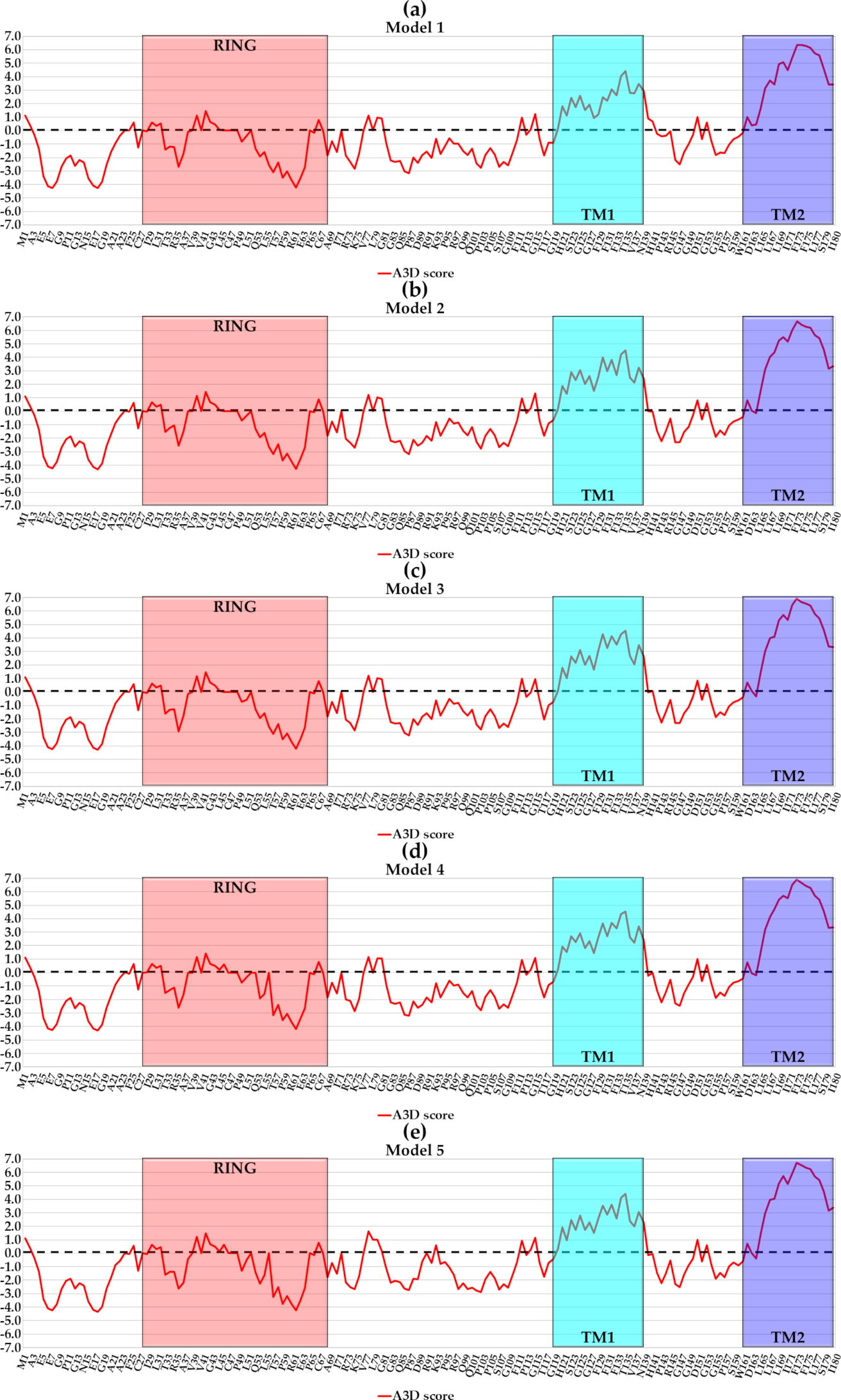


**Fig. S37.** Output graphs reporting the A3D scores (generated by the A3D 2.0 webserver [78]) for the AF2 predicted RNF5 protein structure. (a-e) Each panel refers to a single model (i.e., (a) Model 1; (b) Model 2,….(e) Model 5). The black dashed lines mark the thresholds (i.e., A3D score > 0) defining aggregation-prone residues [78]. Red, cyan and blue shaded boxes highlight the amino acid residues belonging to the RING, first transmembrane (TM1) and second transmembrane (TM2) regions, respectively.

**Table S10.** Aggregation propensity analysis of RNF5 protein by the A3D 2.0 webserver [78]. Output data are reported for each of the best 5 AF2 models of RNF5 structure just for residues belonging to the RING (red residue numbers from 27 to 68), TM1 (cyan residue numbers from 118 to 138) and TM2 (blue residue numbers 160-180) domains. A3D scores > 0 characterizing aggregation-prone residues have been highlighted in green.

| **Residue** | **A3D score** | | | | | **Residue** | **A3D score** | | | | | **Residue** | **A3D score** | | | | |
| --- | --- | --- | --- | --- | --- | --- | --- | --- | --- | --- | --- | --- | --- | --- | --- | --- | --- |
|  | **^#^1^st^** | **2^nd^** | **3^rd^** | **4^th^** | **5^th^** |  | **^#^1^st^** | **2^nd^** | **3^rd^** | **4^th^** | **5^th^** |  | **^#^1^st^** | **2^nd^** | **3^rd^** | **4^th^** | **5^th^** |
| **C27** | 0.0 | 0.0 | 0.0 | 0.0 | 0.0 | **L55** | -2.6 | -2.6 | -2.6 | 0.0 | 0.0 | **G132** | **2.6** | **2.7** | **3.5** | **3.3** | **2.6** |
| **N28** | -0.1 | 0.0 | -0.1 | **0.0** | -0.1 | **E56** | -3.1 | -3.2 | -3.1 | -3.2 | -3.3 | **F133** | **4.0** | **4.2** | **4.3** | **4.3** | **4.1** |
| **I29** | **0.6** | **0.7** | **0.6** | **0.6** | **0.6** | **T57** | -2.4 | -2.4 | -2.4 | -2.4 | -2.5 | **F134** | **4.41** | **4.51** | **4.55** | **4.52** | **4.39** |
| **C30** | **0.4** | **0.3** | **0.3** | **0.3** | **0.3** | **R58** | -3.5 | -3.7 | -3.5 | -3.5 | -3.7 | **T135** | **2.8** | **2.5** | **2.7** | **2.6** | **2.4** |
| **L31** | **0.5** | **0.5** | **0.5** | **0.5** | **0.5** | **P59** | -3.0 | -3.2 | -3.1 | -3.1 | -3.2 | **T136** | **2.8** | **2.1** | **2.1** | **2.2** | **2.0** |
| **E32** | -1.4 | -1.5 | -1.6 | -1.5 | -1.6 | **E60** | -3.7 | -3.7 | -3.7 | -3.7 | -3.7 | **V137** | **3.5** | **3.2** | **3.5** | **3.4** | **3.0** |
| **T33** | -1.2 | -1.2 | -1.3 | -1.3 | -1.4 | **R61** | -4.2 | -4.3 | -4.2 | -4.2 | -4.2 | **F138** | **2.9** | **2.4** | **2.6** | **2.4** | **2.3** |
| **A34** | -1.2 | -1.1 | -1.3 | -1.1 | -1.4 | **Q62** | -3.6 | -3.6 | -3.5 | -3.4 | -3.5 | **S160** | -0.2 | -0.4 | -0.4 | -0.5 | -0.6 |
| **R35** | -2.7 | -2.6 | -2.9 | -2.6 | -2.6 | **E63** | -2.7 | -2.7 | -2.6 | -2.6 | -2.6 | **W161** | **1.0** | **0.8** | **0.7** | **0.8** | **0.7** |
| **E36** | -1.7 | -1.5 | -1.8 | -1.6 | -2.2 | **C64** | 0.0 | 0.0 | 0.0 | 0.0 | 0.0 | **Q162** | **0.4** | **0.0** | **0.1** | -0.1 | **0.0** |
| **A37** | -0.1 | **0.0** | -0.2 | 0.0 | -0.4 | **P65** | -0.2 | -0.1 | -0.1 | -0.1 | -0.1 | **D163** | **0.4** | -0.2 | -0.3 | -0.2 | -0.4 |
| **V38** | 0.0 | 0.0 | 0.0 | 0.0 | 0.0 | **V66** | **0.8** | **0.9** | **0.8** | **0.8** | **0.8** | **S164** | **1.6** | **1.5** | **1.4** | **1.5** | **1.4** |
| **V39** | **1.1** | **1.1** | **1.2** | **1.2** | **1.2** | **C67** | 0.0 | **0.0** | 0.0 | 0.0 | 0.0 | **L165** | **3.1** | **3.1** | **3.0** | **3.2** | **2.9** |
| **S40** | 0.0 | 0.0 | 0.0 | 0.0 | 0.0 | **K68** | -1.9 | -1.8 | -1.8 | -1.8 | -1.8 | **F166** | **3.7** | **4.0** | **4.0** | **4.1** | **4.0** |
| **V41** | **1.4** | **1.4** | **1.5** | **1.4** | **1.5** | **G118** | -0.9 | -0.6 | -0.7 | -0.7 | -0.4 | **L167** | **3.4** | **4.3** | **4.1** | **4.7** | **4.1** |
| **C42** | **0.6** | **0.7** | **0.7** | **0.6** | **0.7** | **G119** | **0.13** | **0.15** | **0.07** | **0.19** | **0.32** | **F168** | **4.9** | **5.2** | **5.3** | **5.4** | **5.2** |
| **G43** | **0.5** | **0.5** | **0.5** | **0.5** | **0.5** | **F120** | **1.82** | **1.89** | **1.78** | **1.93** | **1.91** | **L169** | **5.1** | **5.5** | **5.7** | **5.7** | **5.7** |
| **H44** | **0.1** | **0.1** | **0.1** | **0.2** | **0.1** | **H121** | **1.1** | **1.3** | **1.0** | **1.5** | **0.9** | **A170** | **4.5** | **5.1** | **5.3** | **5.5** | **5.1** |
| **L45** | 0.0 | 0.0 | 0.0 | **0.6** | **0.6** | **F122** | **2.4** | **2.9** | **2.6** | **2.7** | **2.5** | **I171** | **5.4** | **6.0** | **6.5** | **6.5** | **5.9** |
| **Y46** | 0.0 | 0.0 | 0.0 | 0.0 | 0.0 | **S123** | **1.7** | **2.3** | **2.1** | **2.3** | **1.7** | **F172** | **6.3** | **6.6** | **6.9** | **6.9** | **6.7** |
| **C47** | 0.0 | 0.0 | 0.0 | 0.0 | 0.0 | **F124** | **2.6** | **3.1** | **3.1** | **2.9** | **2.8** | **F173** | **6.3** | **6.4** | **6.7** | **6.6** | **6.5** |
| **W48** | 0.0 | 0.0 | 0.0 | 0.0 | 0.0 | **G125** | **1.5** | **2.0** | **2.0** | **1.8** | **1.7** | **F174** | **6.3** | **6.3** | **6.6** | **6.4** | **6.4** |
| **P49** | -0.8 | -0.7 | -0.8 | -0.8 | -1.3 | **V126** | **1.9** | **2.6** | **2.7** | **2.3** | **2.3** | **F175** | **6.1** | **6.2** | **6.4** | **6.3** | **6.2** |
| **C50** | -0.4 | -0.3 | -0.6 | -0.3 | -0.5 | **G127** | **0.9** | **1.5** | **1.6** | **1.4** | **1.5** | **W176** | **5.7** | **5.7** | **5.8** | **5.7** | **5.7** |
| **L51** | 0.0 | 0.0 | 0.0 | 0.0 | 0.0 | **A128** | **1.2** | **2.6** | **3.1** | **2.6** | **2.6** | **L177** | **5.6** | **5.4** | **5.4** | **5.4** | **5.4** |
| **H52** | -1.3 | -1.3 | -1.3 | 0.0 | -1.4 | **F129** | **2.5** | **4.0** | **4.3** | **3.6** | **3.5** | **L178** | **4.6** | **4.6** | **4.6** | **4.6** | **4.6** |
| **Q53** | -1.9 | -1.9 | -1.9 | -1.9 | -2.3 | **P130** | **2.2** | **3.0** | **3.2** | **2.7** | **2.9** | **S179** | **3.4** | **3.1** | **3.4** | **3.3** | **3.2** |
| **W54** | -1.6 | -1.6 | -1.6 | -1.6 | -1.6 | **F131** | **3.0** | **3.8** | **4.1** | **3.7** | **3.6** | **I180** | **3.4** | **3.3** | **3.3** | **3.3** | **3.4** |
| **^#^** Results are shown for each of the best 5 AF2 predicted structure models of the RNF5 protein. | | | | | | | | | | | | | | | | | |

**Fig. S38.** Results from the AggreProt webserver [79]: graphs showing for the best five AF2 predicted structure models of the RNF5 protein, the diverse AggreProt scores. In details, the “Agg.” (i.e., “Aggregation” indicating the aggregation propensity), “SASA” (indicating the Solvent-Accessible Surface Area) and “TM” (i.e., “Transmembrane” indicating the transmembrane propensity) profiles are shown in red, blue and gold, respectively. The black dashed lines mark the thresholds (i.e., Agg. > 0.25 [79], SASA > 0.25 [141

], TM > 0.4 [130]) defining aggregation-prone residues, the solvent accessible ones and those with a tendency to contribute transmembrane regions. In each panel red, cyan and blue shaded boxes encompass the RING, the TM1 and the TM2 regions, respectively.

**Table S11.** Aggregation propensity analysis of RNF5 protein by the AggreProt [79] webserver. The Agg., SASA and TM scores are reported for residues in the RING domain (red), TM1 (cyan) and TM2 (blue) regions: values over the thresholds, that are indicative of prone to aggregate residues, the solvent-accessible ones and those with a tendency to contribute transmembrane regions (i.e., Agg. > 0.25 [79], SASA > 0.25 [141], TM > 0.4 [130] ) are coloured green.

| **Residue** | **Agg.** | | | | | **SASA** | | | | | **TM** | | | | |
| --- | --- | --- | --- | --- | --- | --- | --- | --- | --- | --- | --- | --- | --- | --- | --- |
|  | **1^st^** | **2^nd^** | **3^th^** | **4^th^** | **5^th^** | **1^st^** | **2^nd^** | **3^th^** | **4^th^** | **5^th^** | **1^st^** | **2^nd^** | **3^th^** | **4^th^** | **5^th^** |
| **C27** | **0.55** | **0.55** | **0.55** | **0.55** | **0.55** | 0.0 | 0.0 | 0.0 | 0.0 | 0.0 | 0.00 | 0.00 | 0.00 | 0.00 | 0.00 |
| **N28** | **0.64** | **0.64** | **0.64** | **0.64** | **0.64** | **0.4** | **0.4** | **0.4** | **0.4** | **0.4** | 0.00 | 0.00 | 0.00 | 0.00 | 0.00 |
| **I29** | **0.71** | **0.71** | **0.71** | **0.71** | **0.71** | **0.3** | **0.3** | **0.3** | **0.3** | **0.3** | 0.00 | 0.00 | 0.00 | 0.00 | 0.00 |
| **C30** | **0.64** | **0.64** | **0.64** | **0.64** | **0.64** | **0.3** | **0.3** | **0.3** | **0.3** | **0.3** | 0.00 | 0.00 | 0.00 | 0.00 | 0.00 |
| **L31** | **0.51** | **0.51** | **0.51** | **0.51** | **0.51** | **0.7** | **0.7** | **0.7** | **0.7** | **0.7** | 0.00 | 0.00 | 0.00 | 0.00 | 0.00 |
| **E32** | **0.37** | **0.37** | **0.37** | **0.37** | **0.37** | **0.5** | **0.5** | **0.5** | **0.5** | **0.6** | 0.00 | 0.00 | 0.00 | 0.00 | 0.00 |
| **T33** | 0.23 | 0.23 | 0.23 | 0.23 | 0.23 | **0.5** | **0.5** | **0.5** | **0.5** | **0.5** | 0.00 | 0.00 | 0.00 | 0.00 | 0.00 |
| **A34** | 0.13 | 0.13 | 0.13 | 0.13 | 0.13 | 0.1 | 0.1 | 0.2 | 0.1 | 0.2 | 0.00 | 0.00 | 0.00 | 0.00 | 0.00 |
| **R35** | 0.04 | 0.04 | 0.04 | 0.04 | 0.04 | **0.5** | **0.5** | **0.5** | **0.5** | **0.7** | 0.00 | 0.00 | 0.00 | 0.00 | 0.00 |
| **E36** | 0.14 | 0.14 | 0.14 | 0.14 | 0.14 | 0.2 | 0.2 | 0.2 | 0.2 | **0.4** | 0.00 | 0.00 | 0.00 | 0.00 | 0.00 |
| **A37** | **0.27** | **0.27** | **0.27** | **0.27** | **0.27** | 0.1 | 0.1 | 0.1 | 0.1 | 0.1 | 0.00 | 0.00 | 0.00 | 0.00 | 0.00 |
| **V38** | **0.37** | **0.37** | **0.37** | **0.37** | **0.37** | 0.0 | 0.0 | 0.0 | 0.0 | 0.0 | 0.00 | 0.00 | 0.00 | 0.00 | 0.00 |
| **V39** | **0.42** | **0.42** | **0.42** | **0.42** | **0.42** | 0.2 | 0.2 | 0.2 | 0.2 | 0.2 | 0.00 | 0.00 | 0.00 | 0.00 | 0.00 |
| **S40** | **0.48** | **0.48** | **0.48** | **0.48** | **0.48** | 0.0 | 0.0 | 0.0 | 0.0 | 0.0 | 0.00 | 0.00 | 0.00 | 0.00 | 0.00 |
| **V41** | **0.60** | **0.60** | **0.60** | **0.60** | **0.60** | **0.7** | **0.7** | **0.7** | **0.7** | **0.7** | 0.00 | 0.00 | 0.00 | 0.00 | 0.00 |
| **C42** | **0.64** | **0.64** | **0.64** | **0.64** | **0.64** | 0.3 | 0.3 | 0.3 | 0.3 | 0.3 | 0.00 | 0.00 | 0.00 | 0.00 | 0.00 |
| **G43** | **0.65** | **0.65** | **0.65** | **0.65** | **0.65** | **0.7** | **0.7** | **0.7** | **0.6** | **0.7** | 0.00 | 0.00 | 0.00 | 0.00 | 0.00 |
| **H44** | **0.66** | **0.66** | **0.66** | **0.66** | **0.66** | 0.2 | 0.2 | 0.2 | 0.2 | 0.2 | 0.00 | 0.00 | 0.00 | 0.00 | 0.00 |
| **L45** | **0.71** | **0.71** | **0.71** | **0.71** | **0.71** | 0.1 | 0.1 | 0.1 | 0.1 | 0.1 | 0.00 | 0.00 | 0.00 | 0.00 | 0.00 |
| **Y46** | **0.75** | **0.75** | **0.75** | **0.75** | **0.75** | 0.0 | 0.0 | 0.0 | 0.0 | 0.0 | 0.00 | 0.00 | 0.00 | 0.00 | 0.00 |
| **C47** | **0.68** | **0.68** | **0.68** | **0.68** | **0.68** | 0.0 | 0.0 | 0.0 | 0.0 | 0.0 | 0.00 | 0.00 | 0.00 | 0.00 | 0.00 |
| **W48** | **0.58** | **0.58** | **0.58** | **0.58** | **0.58** | 0.0 | 0.0 | 0.0 | 0.0 | 0.0 | 0.00 | 0.00 | 0.00 | 0.00 | 0.00 |
| **P49** | **0.49** | **0.49** | **0.49** | **0.49** | **0.49** | 0.2 | 0.2 | 0.2 | 0.2 | **0.3** | 0.00 | 0.00 | 0.00 | 0.00 | 0.00 |
| **C50** | **0.51** | **0.51** | **0.51** | **0.51** | **0.51** | 0.2 | 0.2 | 0.2 | 0.2 | 0.2 | 0.00 | 0.00 | 0.00 | 0.00 | 0.00 |
| **L51** | **0.49** | **0.49** | **0.49** | **0.49** | **0.49** | 0.0 | 0.0 | 0.0 | 0.0 | 0.0 | 0.00 | 0.00 | 0.00 | 0.00 | 0.00 |
| **H52** | **0.45** | **0.45** | **0.45** | **0.45** | **0.45** | 0.1 | 0.1 | 0.1 | 0.1 | 0.1 | 0.00 | 0.00 | 0.00 | 0.00 | 0.00 |
| **Q53** | **0.39** | **0.39** | **0.39** | **0.39** | **0.39** | **0.6** | **0.6** | **0.6** | **0.6** | **0.5** | 0.00 | 0.00 | 0.00 | 0.00 | 0.00 |
| **W54** | **0.35** | **0.35** | **0.35** | **0.35** | **0.35** | 0.2 | 0.2 | 0.2 | 0.2 | 0.2 | 0.00 | 0.00 | 0.00 | 0.00 | 0.00 |

| **Residue** | **Agg.** | | | | | **SASA** | | | | | **TM** | | | | |
| --- | --- | --- | --- | --- | --- | --- | --- | --- | --- | --- | --- | --- | --- | --- | --- |
|  | **^#^1^st^** | **2^nd^** | **3^rd^** | **4^th^** | **5^th^** | **^#^1^st^** | **2^nd^** | **3^rd^** | **4^th^** | **5^th^** | **^#^1^st^** | **2^nd^** | **3^rd^** | **4^th^** | **5^th^** |
| **L55** | **0.29** | **0.29** | **0.29** | **0.29** | **0.29** | 0.1 | 0.1 | 0.1 | 0.0 | 0.1 | 0.00 | 0.00 | 0.00 | 0.00 | 0.00 |
| **Q56** | 0.16 | 0.16 | 0.16 | 0.16 | 0.16 | **0.6** | **0.6** | **0.6** | **0.5** | **0.6** | 0.00 | 0.00 | 0.00 | 0.00 | 0.00 |
| **T57** | 0.07 | 0.07 | 0.07 | 0.07 | 0.07 | **0.6** | **0.6** | **0.6** | **0.6** | **0.6** | 0.00 | 0.00 | 0.00 | 0.00 | 0.00 |
| **R58** | 0.01 | 0.01 | 0.01 | 0.01 | 0.01 | **0.4** | **0.5** | **0.4** | **0.4** | **0.5** | 0.00 | 0.00 | 0.00 | 0.00 | 0.00 |
| **P59** | 0.01 | 0.01 | 0.01 | 0.01 | 0.01 | **0.6** | **0.6** | **0.6** | **0.7** | **0.7** | 0.00 | 0.00 | 0.00 | 0.00 | 0.00 |
| **E60** | 0.01 | 0.01 | 0.01 | 0.01 | 0.01 | **0.9** | **0.9** | **0.9** | **0.9** | **0.9** | 0.00 | 0.00 | 0.00 | 0.00 | 0.00 |
| **R61** | 0.02 | 0.02 | 0.02 | 0.02 | 0.02 | **0.7** | **0.6** | **0.7** | **0.7** | **0.7** | 0.00 | 0.00 | 0.00 | 0.00 | 0.00 |
| **Q62** | 0.05 | 0.05 | 0.05 | 0.05 | 0.05 | **0.4** | **0.4** | **0.4** | **0.4** | **0.4** | 0.00 | 0.00 | 0.00 | 0.00 | 0.00 |
| **E63** | 0.08 | 0.08 | 0.08 | 0.08 | 0.08 | **0.4** | **0.4** | **0.4** | **0.4** | **0.3** | 0.00 | 0.00 | 0.00 | 0.00 | 0.00 |
| **C64** | 0.15 | 0.15 | 0.15 | 0.15 | 0.15 | 0.0 | 0.0 | 0.0 | 0.0 | 0.0 | 0.00 | 0.00 | 0.00 | 0.00 | 0.00 |
| **P65** | 0.16 | 0.16 | 0.16 | 0.16 | 0.16 | 0.2 | 0.2 | 0.2 | 0.2 | 0.2 | 0.00 | 0.00 | 0.00 | 0.00 | 0.00 |
| **V66** | 0.22 | 0.22 | 0.22 | 0.22 | 0.22 | **0.4** | **0.4** | **0.4** | **0.4** | **0.4** | 0.00 | 0.00 | 0.00 | 0.00 | 0.00 |
| **C67** | 0.25 | 0.25 | 0.25 | 0.25 | 0.25 | **0.4** | **0.4** | **0.4** | **0.4** | **0.4** | 0.00 | 0.00 | 0.00 | 0.00 | 0.00 |
| **K68** | 0.22 | 0.22 | 0.22 | 0.22 | 0.22 | **0.5** | **0.5** | **0.5** | **0.5** | **0.5** | 0.00 | 0.00 | 0.00 | 0.00 | 0.00 |
| **G118** | 0.13 | 0.13 | 0.13 | 0.13 | 0.13 | **1.0** | **1.0** | **1.0** | **0.9** | **0.8** | **0.66** | **0.66** | **0.66** | **0.66** | **0.66** |
| **G119** | 0.22 | 0.22 | 0.22 | 0.22 | 0.22 | **0.5** | **0.6** | **0.5** | **0.5** | **0.5** | **0.82** | **0.82** | **0.82** | **0.82** | **0.82** |
| **F120** | **0.30** | **0.30** | **0.30** | **0.30** | **0.30** | **0.8** | **0.8** | **0.8** | **0.6** | **0.7** | **0.90** | **0.90** | **0.90** | **0.90** | **0.90** |
| **H121** | **0.39** | **0.39** | **0.39** | **0.39** | **0.39** | **0.5** | **0.6** | **0.5** | **0.4** | **0.8** | **0.93** | **0.93** | **0.93** | **0.93** | **0.93** |
| **F122** | **0.43** | **0.43** | **0.43** | **0.43** | **0.43** | **0.6** | **0.7** | **0.7** | **0.6** | **0.6** | **0.97** | **0.97** | **0.97** | **0.97** | **0.97** |
| **S123** | **0.46** | **0.46** | **0.46** | **0.46** | **0.46** | **0.7** | **0.3** | **0.5** | **0.4** | **0.8** | **0.98** | **0.98** | **0.98** | **0.98** | **0.98** |
| **F124** | **0.45** | **0.45** | **0.45** | **0.45** | **0.45** | **0.7** | **0.7** | **0.6** | **0.8** | **0.7** | **0.98** | **0.98** | **0.98** | **0.98** | **0.98** |
| **G125** | **0.38** | **0.38** | **0.38** | **0.38** | **0.38** | **0.5** | **0.4** | **0.4** | **0.5** | **0.5** | **0.98** | **0.98** | **0.98** | **0.98** | **0.98** |
| **V126** | **0.32** | **0.32** | **0.32** | **0.32** | **0.32** | **1.0** | **0.9** | **0.9** | **0.8** | **0.8** | **0.98** | **0.98** | **0.98** | **0.98** | **0.98** |
| **G127** | 0.23 | 0.23 | 0.23 | 0.23 | 0.23 | **0.7** | **0.6** | **0.6** | **0.8** | **0.6** | **0.98** | **0.98** | **0.98** | **0.98** | **0.98** |
| **A128** | 0.19 | 0.19 | 0.19 | 0.19 | 0.19 | **0.6** | **0.6** | **0.5** | **0.5** | **0.3** | **0.98** | **0.98** | **0.98** | **0.98** | **0.98** |
| **F129** | 0.17 | 0.17 | 0.17 | 0.17 | 0.17 | 0.2 | **0.6** | **0.5** | **0.8** | **0.7** | **0.98** | **0.98** | **0.98** | **0.98** | **0.98** |
| **P130** | 0.17 | 0.17 | 0.17 | 0.17 | 0.17 | **0.6** | **0.4** | **0.4** | **0.5** | **0.4** | **0.98** | **0.98** | **0.98** | **0.98** | **0.98** |
| **F131** | 0.23 | 0.23 | 0.23 | 0.23 | 0.23 | **0.7** | **0.7** | **0.6** | **0.6** | **0.6** | **0.98** | **0.98** | **0.98** | **0.98** | **0.98** |

| **Residue** | **Agg.** | | | | | **SASA** | | | | | **TM** | | | | |
| --- | --- | --- | --- | --- | --- | --- | --- | --- | --- | --- | --- | --- | --- | --- | --- |
|  | **1^st^** | **2^nd^** | **3^rd^** | **4^th^** | **5^th^** | **1^st^** | **2^nd^** | **3^rd^** | **4^th^** | **5^th^** | **1^st^** | **2^nd^** | **3^rd^** | **4^th^** | **5^th^** |
| **G132** | **0.31** | **0.31** | **0.31** | **0.31** | **0.31** | 0.2 | **0.5** | **0.3** | **0.3** | **0.5** | **0.98** | **0.98** | **0.98** | **0.98** | **0.98** |
| **F133** | **0.44** | **0.44** | **0.44** | **0.44** | **0.44** | **0.6** | **0.6** | **0.5** | **0.7** | **0.5** | **0.98** | **0.98** | **0.98** | **0.98** | **0.98** |
| **F134** | **0.51** | **0.51** | **0.51** | **0.51** | **0.51** | **0.6** | **0.5** | **0.5** | **0.5** | **0.5** | **0.98** | **0.98** | **0.98** | **0.98** | **0.98** |
| **T135** | **0.54** | **0.54** | **0.54** | **0.54** | **0.54** | **0.3** | **0.5** | **0.5** | **0.5** | **0.5** | **0.98** | **0.98** | **0.98** | **0.98** | **0.98** |
| **T136** | **0.55** | **0.55** | **0.55** | **0.55** | **0.55** | **0.5** | **0.7** | **0.6** | **0.5** | **0.6** | **0.98** | **0.98** | **0.98** | **0.98** | **0.98** |
| **V137** | **0.52** | **0.52** | **0.52** | **0.52** | **0.52** | **0.6** | **0.5** | **0.5** | **0.5** | **0.5** | **0.96** | **0.96** | **0.96** | **0.96** | **0.96** |
| **F138** | **0.43** | **0.43** | **0.43** | **0.43** | **0.43** | **0.7** | **0.6** | **0.7** | **0.7** | **0.6** | **0.95** | **0.95** | **0.95** | **0.95** | **0.95** |
| **S160** | 0.03 | 0.03 | 0.03 | 0.03 | 0.03 | **0.6** | **0.7** | **0.7** | **0.6** | **0.6** | **0.72** | **0.72** | **0.72** | **0.72** | **0.72** |
| **W161** | 0.05 | 0.05 | 0.05 | 0.05 | 0.05 | **0.6** | **0.6** | **0.6** | **0.6** | **0.6** | **0.89** | **0.89** | **0.89** | **0.89** | **0.89** |
| **Q162** | 0.09 | 0.09 | 0.09 | 0.09 | 0.09 | **0.4** | **0.4** | **0.4** | **0.5** | **0.4** | **0.91** | **0.91** | **0.91** | **0.91** | **0.91** |
| **D163** | 0.16 | 0.16 | 0.16 | 0.16 | 0.16 | **0.4** | **0.6** | **0.6** | **0.6** | **0.6** | **0.92** | **0.92** | **0.92** | **0.92** | **0.92** |
| **S164** | **0.29** | **0.29** | **0.29** | **0.29** | **0.29** | **0.4** | **0.4** | **0.4** | **0.4** | **0.4** | **0.97** | **0.97** | **0.97** | **0.97** | **0.97** |
| **L165** | **0.43** | **0.43** | **0.43** | **0.43** | **0.43** | **0.5** | **0.4** | **0.5** | **0.5** | **0.4** | **0.99** | **0.99** | **0.99** | **0.99** | **0.99** |
| **F166** | **0.56** | **0.56** | **0.56** | **0.56** | **0.56** | **0.6** | **0.6** | **0.6** | **0.6** | **0.6** | **0.99** | **0.99** | **0.99** | **0.99** | **0.99** |
| **L167** | **0.69** | **0.69** | **0.69** | **0.69** | **0.69** | **0.3** | **0.6** | **0.4** | **0.6** | **0.5** | **0.99** | **0.99** | **0.99** | **0.99** | **0.99** |
| **F168** | **0.80** | **0.80** | **0.80** | **0.80** | **0.80** | **0.6** | **0.6** | **0.6** | **0.6** | **0.6** | **0.99** | **0.99** | **0.99** | **0.99** | **0.99** |
| **L169** | **0.88** | **0.88** | **0.88** | **0.88** | **0.88** | **0.5** | **0.5** | **0.5** | **0.5** | **0.5** | **0.99** | **0.99** | **0.99** | **0.99** | **0.99** |
| **A170** | **0.89** | **0.89** | **0.89** | **0.89** | **0.89** | **0.4** | **0.5** | **0.5** | **0.5** | **0.5** | **0.99** | **0.99** | **0.99** | **0.99** | **0.99** |
| **I171** | **0.91** | **0.91** | **0.91** | **0.91** | **0.91** | **0.4** | **0.4** | **0.5** | **0.5** | **0.4** | **0.99** | **0.99** | **0.99** | **0.99** | **0.99** |
| **F172** | **0.87** | **0.87** | **0.87** | **0.87** | **0.87** | **0.6** | **0.6** | **0.6** | **0.6** | **0.6** | **0.99** | **0.99** | **0.99** | **0.99** | **0.99** |
| **F173** | **0.82** | **0.82** | **0.82** | **0.82** | **0.82** | **0.5** | **0.5** | **0.5** | **0.5** | **0.5** | **0.99** | **0.99** | **0.99** | **0.99** | **0.99** |
| **F174** | **0.80** | **0.80** | **0.80** | **0.80** | **0.80** | **0.5** | **0.6** | **0.6** | **0.6** | **0.6** | **0.99** | **0.99** | **0.99** | **0.99** | **0.99** |
| **F175** | **0.78** | **0.78** | **0.78** | **0.78** | **0.78** | **0.6** | **0.6** | **0.6** | **0.6** | **0.6** | **0.99** | **0.99** | **0.99** | **0.99** | **0.99** |
| **W176** | **0.75** | **0.75** | **0.75** | **0.75** | **0.75** | **0.5** | **0.5** | **0.5** | **0.5** | **0.5** | **0.99** | **0.99** | **0.99** | **0.99** | **0.99** |
| **L177** | **0.71** | **0.71** | **0.71** | **0.71** | **0.71** | **0.6** | **0.6** | **0.6** | **0.6** | **0.6** | **0.98** | **0.98** | **0.98** | **0.98** | **0.98** |
| **L178** | **0.74** | **0.74** | **0.74** | **0.74** | **0.74** | **0.6** | **0.6** | **0.6** | **0.6** | **0.6** | **0.86** | **0.86** | **0.86** | **0.86** | **0.86** |
| **S179** | **0.82** | **0.82** | **0.82** | **0.82** | **0.82** | **0.7** | **0.7** | **0.7** | **0.7** | **0.7** | **0.45** | **0.45** | **0.45** | **0.45** | **0.45** |
| **I180** | **0.83** | **0.83** | **0.83** | **0.83** | **0.83** | **0.8** | **0.9** | **0.9** | **0.9** | **0.9** | 0.00 | 0.00 | 0.00 | 0.00 | 0.00 |
| “Agg.” stands for “Aggregation” score and indicates the aggregation propensity. “SASA” = score indicating solvent-accessible surface area. = “TM” stands for “Transmembrane” score indicating the transmembrane propensity | | | | | | | | | | | | | | | |
| **^#^** Results are shown for each of the best 5 AF2 predicted structure models of the RNF5 protein. | | | | | | | | | | | | | | | |


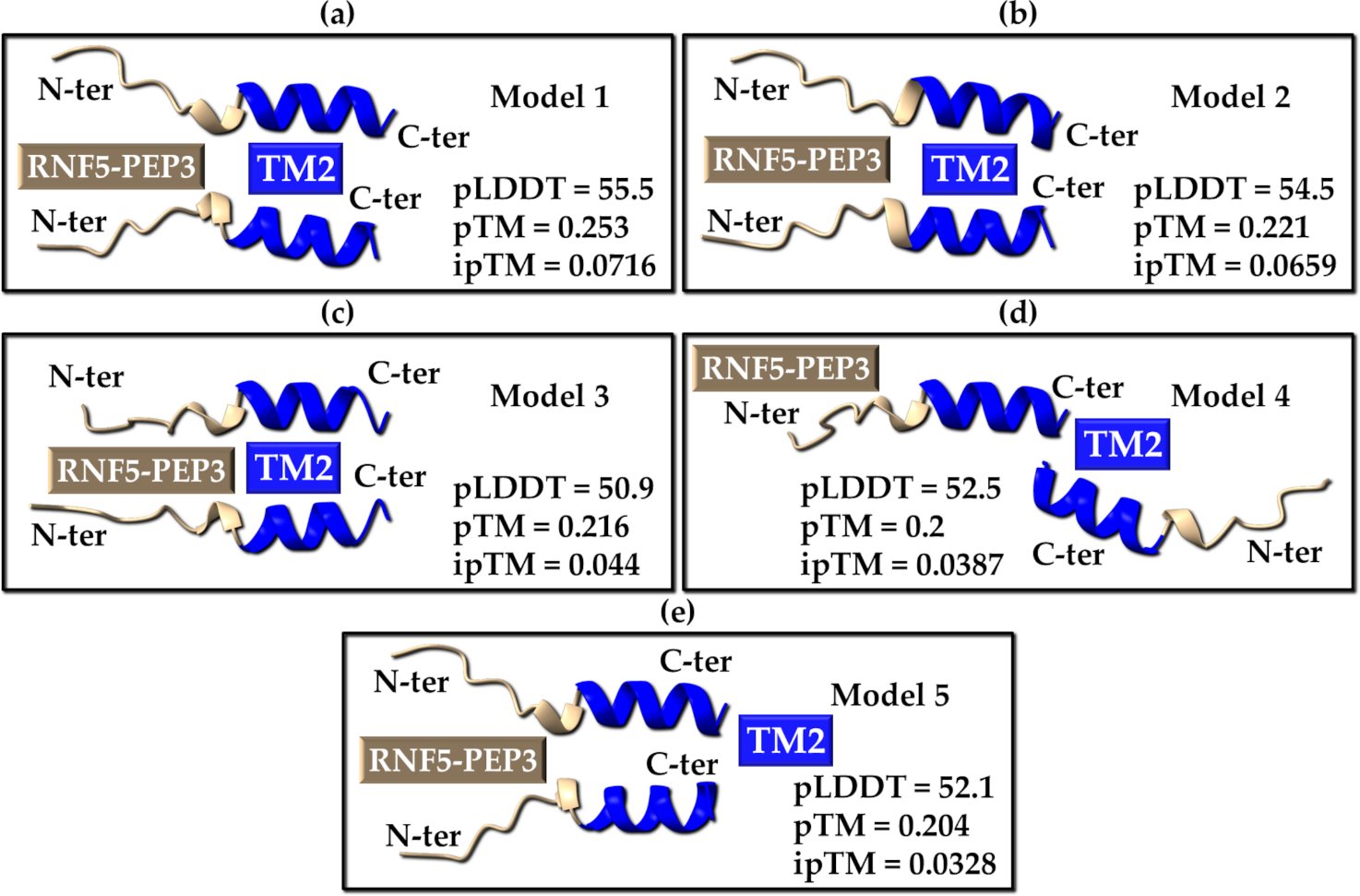


**Figure S39.**(a-e) AF2 [49,55] homodimeric models (from the 1^st^ to the 5^th^ ranked structures) of the RNF5-PEP3 peptide (residues D151-A170 from UniProt [57] entry Q99942 for human RNF5). Blue is used to indicate the residues belonging to the second transmembrane segment (TM2) (S160-I180, UniProt code Q99942) of RNF5 protein. The confidence pLDDT, pTM and ipTM scores of all models are indicated in each panel.

.


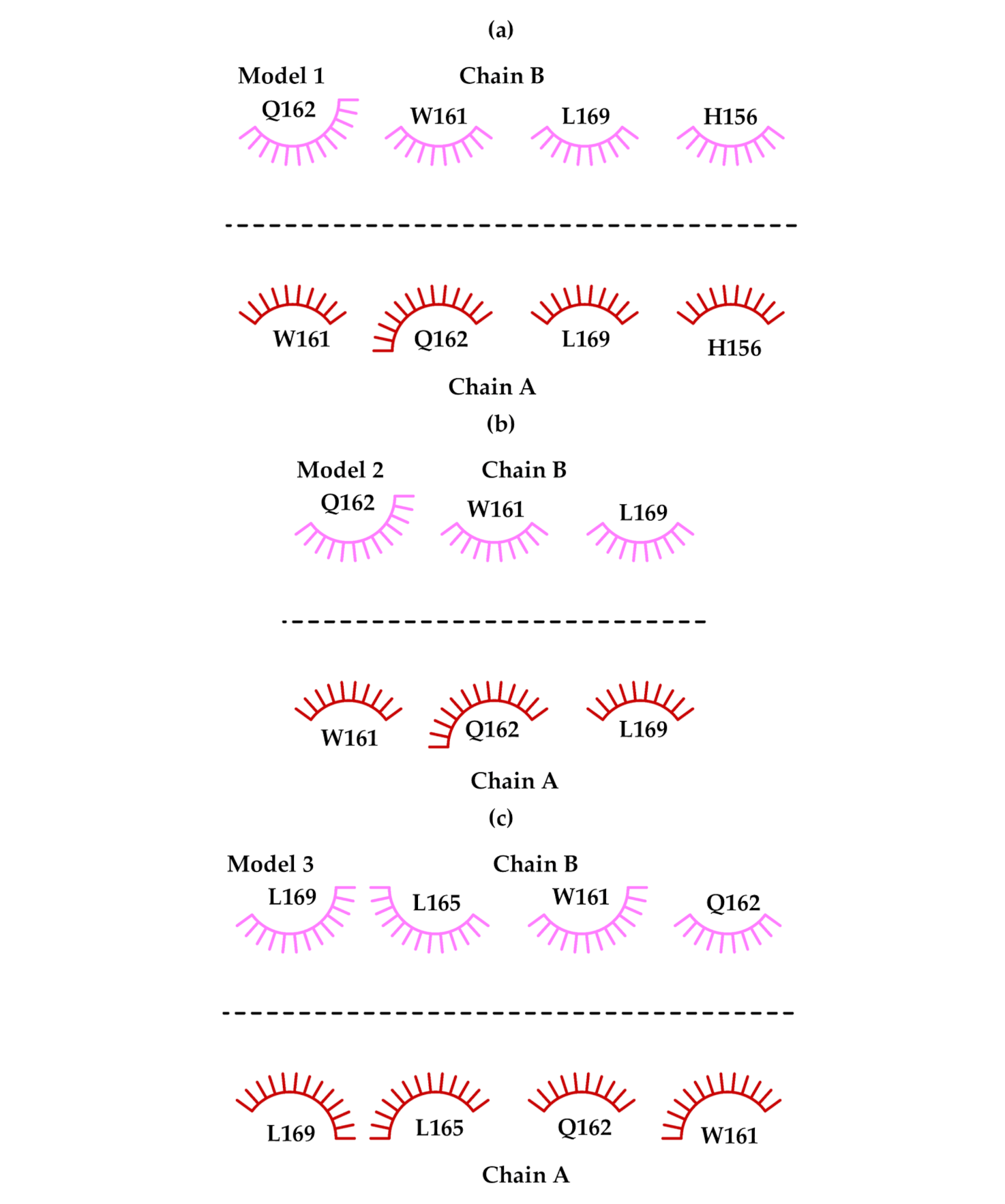


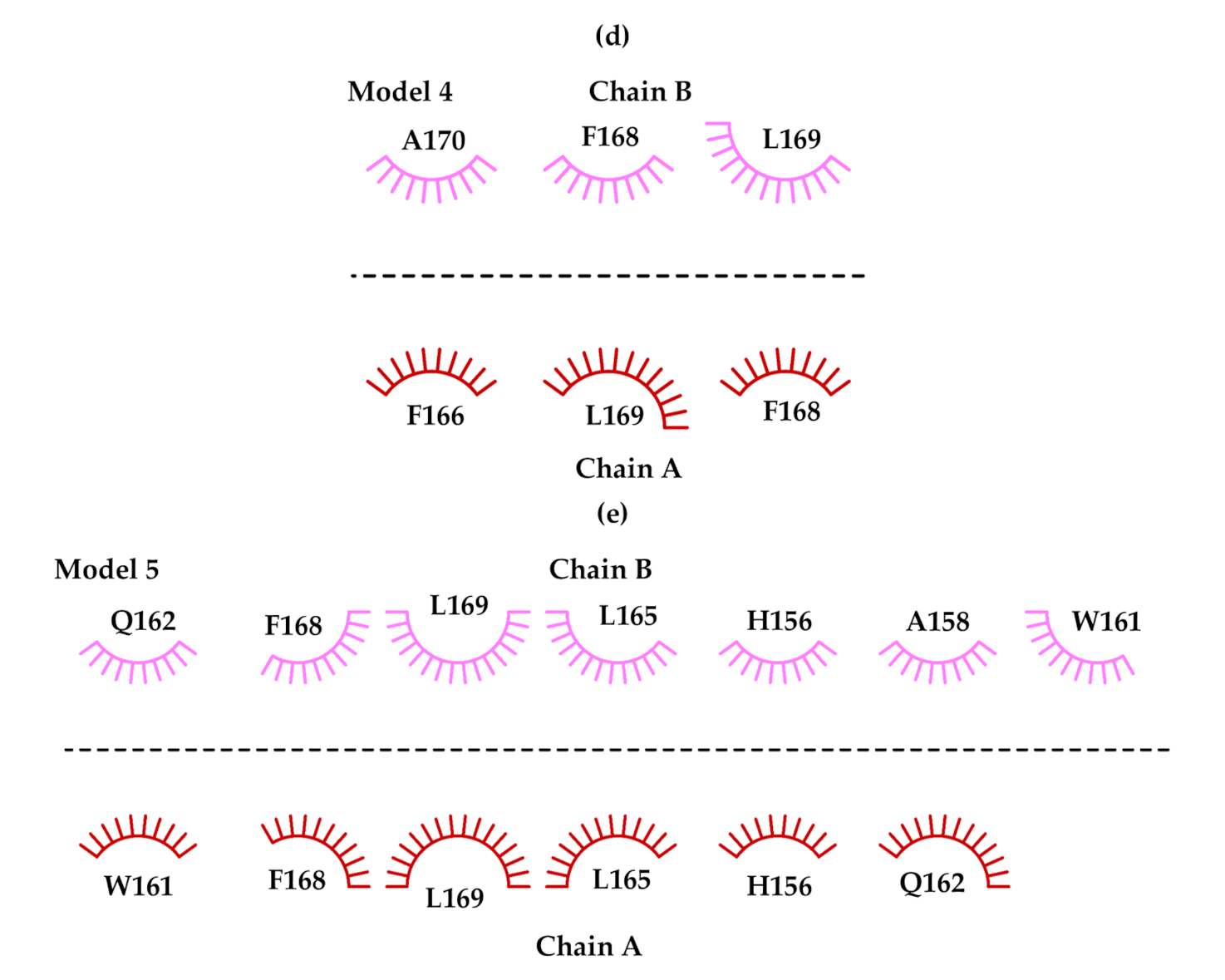


**Fig. S40.** 2D diagrams of intermolecular interactions generated by LigPlot+ [63,64] for the AF2 [49,55] homodimeric models of the RNF5-PEP3 peptide (residues D151-A170 from UniProt [57] entry Q99942 for human RNF5). (a-e) 1^st^ to 5^th^ ranked AF2 models.

**Table S12.** RNF5-PEP3 peptide homodimer: statistics of interchain H-bonds and non-bonded interactions. The global number of intermolecular interactions was derived by summing for each residue of the RNF5-PEP3 peptide (a.a. D151-A170 from UniProt [57] entry Q99942 for human RNF5) within all 5 AF2 models, the corresponding list of contacts generated by LigPlot+ [63,64].

| **RNF5-PEP3 residues** | **Number of**  **H-bonds** | | **Number of**  **non-bonded interactions** | |
| --- | --- | --- | --- | --- |
|  | **Chain A** | **Chain B** | **Chain A** | **Chain B** |
| H156 | 0 | 0 | 14 | 14 |
| A158 | 0 | 0 | 0 | 1 |
| W161 | 0 | 0 | 33 | 38 |
| Q162 | 0 | 0 | 45 | 39 |
| L165 | 0 | 0 | 10 | 10 |
| F166 | 0 | 0 | 1 | 0 |
| F168 | 0 | 0 | 3 | 4 |
| L169 | 0 | 0 | 62 | 61 |
| A170 | 0 | 0 | 0 | 1 |

**
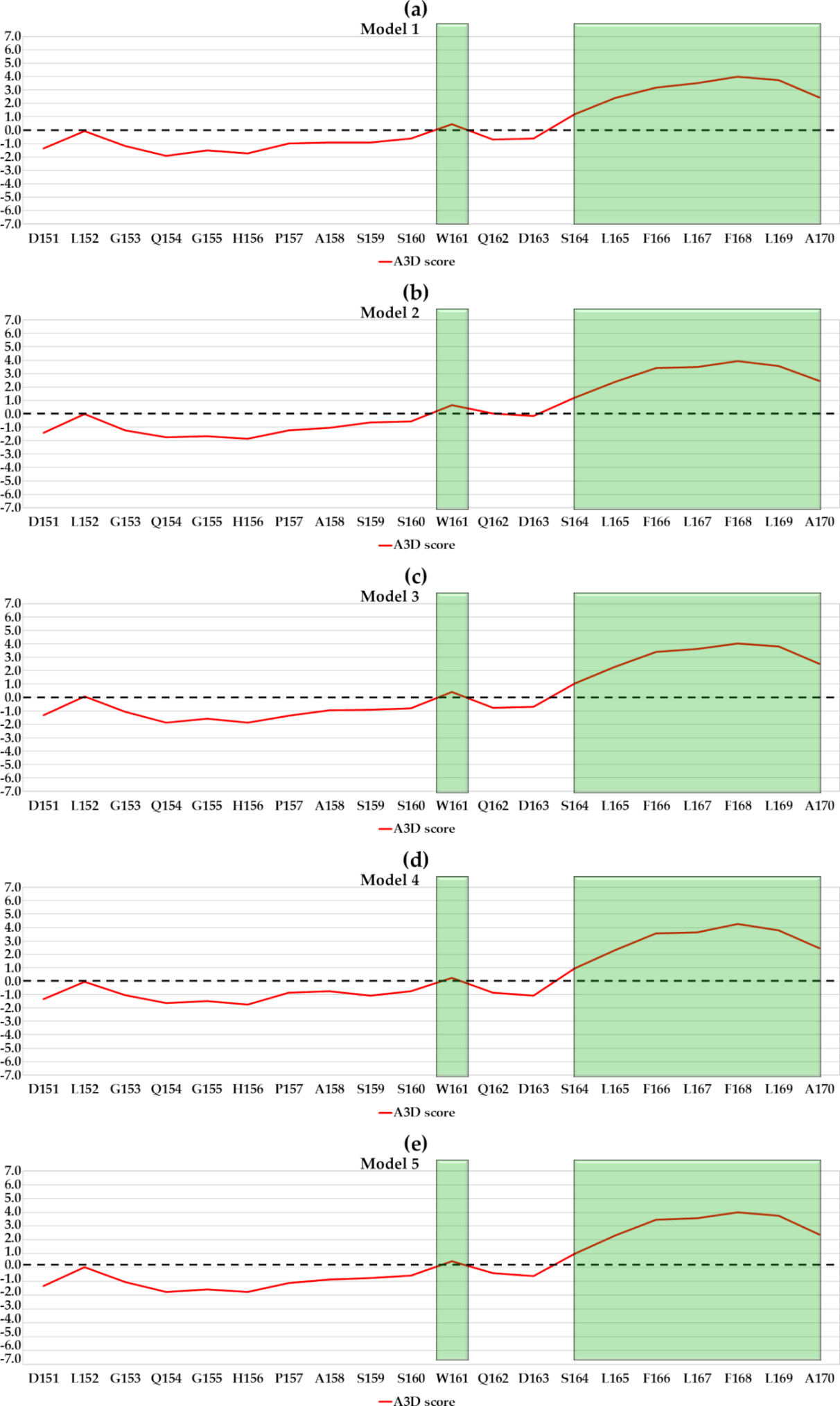
**

**Fig. S41.** Graphs reporting A3D scores (generated by the A3D 2.0 webserver) [78] for the five AF2 models of RNF5-PEP3 peptide. The black dashed lines highlight the thresholds (i.e., A3D score > 0) defining aggregation-prone residues.

**Table S13.** Aggregation propensity analysis of RNF5-PEP3 peptide by the A3D 2.0 webserver [78]. A3D scores pointing out aggregation-prone residues (i.e., A3D score > 0) have been highlighted in green.

| **Residue** | **A3D score** | | | | |
| --- | --- | --- | --- | --- | --- |
|  | **^#^1^st^** | **2^nd^** | **3^th^** | **4^th^** | **5^th^** |
| **W161** | **0.4** | **0.6** | **0.4** | **0.2** | **0.4** |
| **S164** | **1.2** | **1.2** | **1.0** | **0.9** | **1.0** |
| **L165** | **2.4** | **2.4** | **2.3** | **2.3** | **2.3** |
| **F166** | **3.2** | **3.4** | **3.4** | **3.5** | **3.5** |
| **L167** | **3.5** | **3.5** | **3.6** | **3.6** | **3.6** |
| **F168** | **4.0** | **3.9** | **4.0** | **4.3** | **4.0** |
| **L169** | **3.7** | **3.6** | **3.8** | **3.8** | **3.7** |
| **A170** | **2.4** | **2.4** | **2.5** | **2.5** | **2.4** |
| ^#^Results are shown for each of the five models representing the AF2 predicted RNF5-PEP3 structure | | | | | |

**
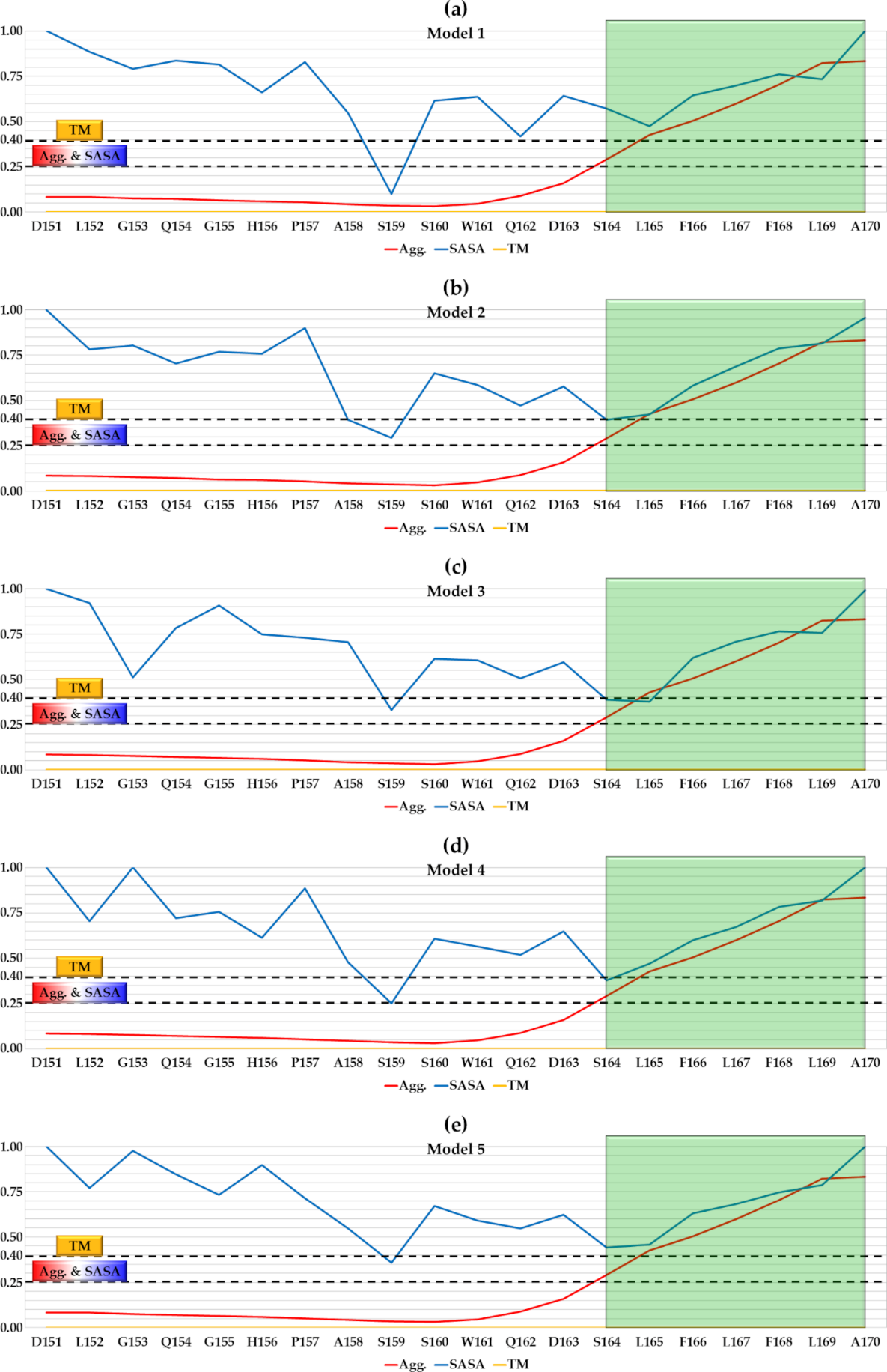
Fig. S42.** Results from the AggreProt webserver [79]: graphs showing for the best five AF2 predicted models of RNF5-PEP3 peptide (residues D151-A170 from UniProt entry Q99942 for human RNF5), the diverse AggreProt scores. In details, the “Agg.”, “SASA” and “TM” profiles are shown in red, blue and gold, respectively. The black dashed lines mark the thresholds (i.e., Agg. > 0.25 [79], SASA > 0.25 [141], TM > 0.4 [130]) defining aggregation- prone residues, the solvent accessible ones and those that could participate in transmembrane regions. In each panel red, cyan and blue shaded boxes include the RING, the TM1 and the TM2 domains, respectively.

**Table S14.** Aggregation propensity analysis of the RNF5-PEP3 peptide by the AggreProt [79] webserver. The “Agg.”, “SASA” and “TM” score values over the thresholds, that are indicative of prone to aggregate residues, the solvent-accessible ones and those with a tendency to contribute transmembrane regions (i.e., Agg. > 0.25 [79], SASA > 0.25 [141], TM > 0.4 [130]), respectively, are coloured green.

| **Residue** | **Agg.** | | | | | **SASA** | | | | | **TM** | | | | |
| --- | --- | --- | --- | --- | --- | --- | --- | --- | --- | --- | --- | --- | --- | --- | --- |
|  | **^#^1^st^** | **2^nd^** | **3^th^** | **4^th^** | **5^th^** | **^#^1^st^** | **2^nd^** | **3^th^** | **4^th^** | **5^th^** | **^#^1^st^** | **2^nd^** | **3^th^** | **4^th^** | **5^th^** |
| **S164** | **0.29** | **0.29** | **0.29** | **0.29** | **0.29** | **0.57** | **0.39** | **0.39** | **0.38** | **0.44** | 0.00 | 0.00 | 0.00 | 0.00 | 0.00 |
| **L165** | **0.43** | **0.43** | **0.43** | **0.43** | **0.43** | **0.47** | **0.42** | **0.38** | **0.47** | **0.46** | 0.00 | 0.00 | 0.00 | 0.00 | 0.00 |
| **F166** | **0.50** | **0.50** | **0.50** | **0.50** | **0.50** | **0.64** | **0.58** | **0.62** | **0.60** | **0.63** | 0.00 | 0.00 | 0.00 | 0.00 | 0.00 |
| **L167** | **0.60** | **0.60** | **0.60** | **0.60** | **0.60** | **0.70** | **0.69** | **0.71** | **0.67** | **0.68** | 0.00 | 0.00 | 0.00 | 0.00 | 0.00 |
| **F168** | **0.70** | **0.70** | **0.70** | **0.70** | **0.70** | **0.76** | **0.79** | **0.77** | **0.78** | **0.75** | 0.00 | 0.00 | 0.00 | 0.00 | 0.00 |
| **L169** | **0.82** | **0.82** | **0.82** | **0.82** | **0.82** | **0.73** | **0.81** | **0.76** | **0.82** | **0.79** | 0.00 | 0.00 | 0.00 | 0.00 | 0.00 |
| **A170** | **0.83** | **0.83** | **0.83** | **0.83** | **0.83** | **1.00** | **0.96** | **0.99** | **1.00** | **1.00** | 0.00 | 0.00 | 0.00 | 0.00 | 0.00 |
| ^#^ Results are shown for each of the best five models representing the AF2 predicted RNF5-PEP3 structure | | | | | | | | | | | | | | | |

**References**:

41. Lee HJ, Hota PK, Chugha P, Guo H, Miao H, Zhang LQ*, et al.* NMR Structure of a Heterodimeric SAM:SAM Complex: Characterization and Manipulation of EphA2 Binding Reveal New Cellular Functions of SHIP2. Structure 2012;20:41–55.DOI: 10.1016/j.str.2011.11.013.

57. UniProt Consortium. UniProt: the Universal Protein Knowledgebase in 2025. Nucleic Acids Res 2025;53:D609–D17.DOI: 10.1093/nar/gkae1010.

49. Jumper J, Evans R, Pritzel A, Green T, Figurnov M, Ronneberger O*, et al.* Highly accurate protein structure prediction with AlphaFold. Nature 2021;596:583–9.DOI: 10.1038/s41586-021-03819-2.

55. Varadi M, Anyango S, Deshpande M, Nair S, Natassia C, Yordanova G*, et al.* AlphaFold Protein Structure Database: massively expanding the structural coverage of protein-sequence space with high-accuracy models. Nucleic Acids Res 2022;50:D439–D44.DOI: 10.1093/nar/gkab1061.

51. Abramson J, Adler J, Dunger J, Evans R, Green T, Pritzel A*, et al.* Addendum: Accurate structure prediction of biomolecular interactions with AlphaFold 3. Nature 2024;636:E4.DOI: 10.1038/s41586-024-08416-7.

52. Abramson J, Adler J, Dunger J, Evans R, Green T, Pritzel A*, et al.* Accurate structure prediction of biomolecular interactions with AlphaFold 3. Nature 2024;630:493–500.DOI: 10.1038/s41586-024-07487-w.

137. Guo HB, Perminov A, Bekele S, Kedziora G, Farajollahi S, Varaljay V*, et al.* AlphaFold2 models indicate that protein sequence determines both structure and dynamics. Sci Rep 2022;12:10696.DOI: 10.1038/s41598-022-14382-9.

138. Yin R, Feng BY, Varshney A, Pierce BG. Benchmarking AlphaFold for protein complex modeling reveals accuracy determinants. Protein Sci 2022;31:e4379.DOI: 10.1002/pro.4379.

139. Zhang Y, Skolnick J. Scoring function for automated assessment of protein structure template quality. Proteins 2004;57:702–10.DOI: 10.1002/prot.20264.

107. 01/09/2025. Section 3: Interpreting results from AlphaFold Server. <<https://alphafoldserver.com/guides#section-3:-interpreting-results-from-alphafold-server> >. Accessed 2025 01/09/2025.

60. Meng EC, Goddard TD, Pettersen EF, Couch GS, Pearson ZJ, Morris JH*, et al.* UCSF ChimeraX: Tools for structure building and analysis. Protein Sci 2023;32:e4792.DOI: 10.1002/pro.4792.

102. 2024 03/02/2026. Google Deepmind and EMBL-EBI. How to assess the quality of AlphaFold 3 predictions. <<https://www.ebi.ac.uk/training/online/courses/alphafold/alphafold-3-and-alphafold-server/how-to-assess-the-quality-of-alphafold-3-predictions/>>. Accessed 2026 03/02/2026.

59. Kim G, Lee S, Levy Karin E, Kim H, Moriwaki Y, Ovchinnikov S*, et al.* Easy and accurate protein structure prediction using ColabFold. Nat Protoc 2025;20:620–42.DOI: 10.1038/s41596-024-01060-5.

108. EMBL’s European Bioinformatics Institute and Google Deepmind. Outputs from ColabFold. <<https://www.ebi.ac.uk/training/online/courses/alphafold/advanced-modeling-and-applications-of-predicted-protein-structures/customising-alphafold-structure-predictions/outputs-from-colabfold/>>.

110. Erdos G, Pajkos M, Dosztanyi Z. IUPred3: prediction of protein disorder enhanced with unambiguous experimental annotation and visualization of evolutionary conservation. Nucleic Acids Res 2021;49:W297–W303.DOI: 10.1093/nar/gkab408.

113. Hallgren J, Tsirigos KD, Pedersen MD, Almagro Armenteros JJ, Marcatili P, Nielsen H*, et al.* DeepTMHMM predicts alpha and beta transmembrane proteins using deep neural networks. bioRxiv 2022:2022.04.08.487609.DOI: 10.1101/2022.04.08.487609.

114. Hessa T, Meindl-Beinker NM, Bernsel A, Kim H, Sato Y, Lerch-Bader M*, et al.* Molecular code for transmembrane-helix recognition by the Sec61 translocon. Nature 2007;450:1026–30.DOI: 10.1038/nature06387.

112. Gutierrez S, Tyczynski WG, Boomsma W, Teufel F, Winther O. MembraneFold: Visualising transmembrane protein structure and topology. bioRxiv 2022:2022.12.06.518085.DOI: <https://doi.org/10.1101/2022.12.06.518085>.

63. Wallace AC, Laskowski RA, Thornton JM. LIGPLOT: a program to generate schematic diagrams of protein-ligand interactions. Protein Eng 1995;8:127–34.DOI: 10.1093/protein/8.2.127.

64. Laskowski RA, Swindells MB. LigPlot+: multiple ligand-protein interaction diagrams for drug discovery. J Chem Inf Model 2011;51:2778–86.DOI: 10.1021/ci200227u.

54. Honorato RV, Trellet ME, Jimenez-Garcia B, Schaarschmidt JJ, Giulini M, Reys V*, et al.* The HADDOCK2.4 web server for integrative modeling of biomolecular complexes. Nat Protoc 2024;19:3219–41.DOI: 10.1038/s41596-024-01011-0.

66. Micsonai A, Moussong E, Wien F, Boros E, Vadaszi H, Murvai N*, et al.* BeStSel: webserver for secondary structure and fold prediction for protein CD spectroscopy. Nucleic Acids Res 2022;50:W90–W8.DOI: 10.1093/nar/gkac345.

140. Vuister GW, Bax A. Resolution enhancement and spectral editing of uniformly 13C-enriched proteins by homonuclear broadband 13C decoupling. Journal of Magnetic Resonance (1969) 1992;98:428–35.DOI: <https://doi.org/10.1016/0022-2364(92)90144-V>.

126. Pettitt AJ, Shukla VK, Figueiredo AM, Newton LS, McCarthy S, Tabor AB*, et al.* An integrative characterization of proline cis and trans conformers in a disordered peptide. Biophys J 2024;123:3798–811.DOI: 10.1016/j.bpj.2024.09.028.

74. Wuthrich K. NMR of Proteins and Nucleic Acids. New YorK: Wiley; 1986.

78. Kuriata A, Iglesias V, Pujols J, Kurcinski M, Kmiecik S, Ventura S. Aggrescan3D (A3D) 2.0: prediction and engineering of protein solubility. Nucleic Acids Res 2019;47:W300–W7.DOI: 10.1093/nar/gkz321.

79. Planas-Iglesias J, Borko S, Swiatkowski J, Elias M, Havlasek M, Salamon O*, et al.* AggreProt: a web server for predicting and engineering aggregation prone regions in proteins. Nucleic Acids Res 2024;52:W159–W69.DOI: 10.1093/nar/gkae420.

141. Schmidt C, Macpherson JA, Lau AM, Tan KW, Fraternali F, Politis A. Surface Accessibility and Dynamics of Macromolecular Assemblies Probed by Covalent Labeling Mass Spectrometry and Integrative Modeling. Anal Chem 2017;89:1459–68.DOI: 10.1021/acs.analchem.6b02875.

130. Cserzo M, Eisenhaber F, Eisenhaber B, Simon I. TM or not TM: transmembrane protein prediction with low false positive rate using DAS-TMfilter. Bioinformatics 2004;20:136–7.DOI: 10.1093/bioinformatics/btg394.
